# Dose-response-defined physiological framework enables comparative analysis of transcriptional response dynamics across multiple herbicides in *Arabidopsis thaliana*

**DOI:** 10.3389/fpls.2026.1870671

**Published:** 2026-09-23

**Authors:** Jing Xi, Ameeta K. Agarwal, Julie A. Blessitt, Melanie M. Mask, Yongjian Qiu, Joanna Bajsa-Hirschel, Zhiqiang Pan, Charles L. Cantrell, Scott R. Baerson

**Affiliations:** 1 Natural Products Utilization Research Unit, Agricultural Research Service, United States Department of Agriculture, University, MS, United States; 2 National Center for Natural Products Research, School of Pharmacy, University of Mississippi, University, MS, United States; 3 Department of Biology, University of Mississippi, University, MS, United States

**Keywords:** *Arabidopsis thaliana*, dose- and time-related, dose-response, gene regulation, herbicide mode of action, physiological responses, RNA-sequencing

## Abstract

Herbicides elicit diverse physiological responses in plants largely due to distinct modes of action of individual compounds. However, it remains unclear how plants differentially respond to herbicides with distinct modes of action when applied at herbicide-specific treatment levels corresponding to comparable degrees of growth inhibition. In this study, we integrated physiological and transcriptomic analyses to characterize the responses of *Arabidopsis thaliana* exposed to seven herbicides (2,4-dichlorophenoxyacetic acid, acifluorfen, atrazine, glyphosate, imazethapyr, mesotrione, and paraquat) representing seven different modes of action. Dose-response experiments were first conducted to determine herbicide-specific inhibitory concentrations, establishing a framework of physiologically defined treatment levels based on growth inhibition for subsequent RNA sequencing. Comparative transcriptomic analysis under a pairwise design with two points (4 hour and 24 hour) and two dosage levels (half-maximal inhibitory concentration and 99% inhibitory concentration) at each time point revealed herbicide-specific differentially expressed genes. Temporal expression dynamics of these unique gene sets via hierarchical clustering further resolved distinct herbicide mode-of-action-associated and shared broader stress response-related, growth inhibition-related, and detoxification-related processes with limited overlaps at the gene level. Together, these results demonstrate that plants display divergent physiological and transcriptional responses to different herbicides under comparable levels of growth inhibition yet converge on common stress-response biological effects. These findings indicate how plants coordinate physiological and transcriptional responses to adapt to distinct chemical perturbations. The identified differentially expressed gene sets and their associated expression patterns provide a framework for herbicide mode-of-action inference, classification of novel compounds, and investigation of herbicide resistance mechanisms at the molecular level.

## 1 Introduction

Herbicides impose diverse physiological constraints on plants by acting through a wide range of modes of action (MOA), including inhibition of amino acid biosynthesis, disruption of photosynthesis, inhibition of fatty acid and lipid synthesis, interference with cell division and hormone signaling pathways (Duke and Dayan, 2022). These perturbations trigger complex physiological responses, including metabolic reprogramming and stress-related responses, which vary substantially among herbicides (Hasan et al., 2022). Understanding how different herbicides alter plant physiological states is fundamental to elucidating their biological effects and MOAs. Such understanding is also essential for sustainable weed management, which allows informed selection and rotation, resistance management, and effective diagnosis of crop responses (Dayan et al., 2026).

A key challenge in studying herbicide-induced physiological responses lies in establishing a quantitative framework for comparing multiple compounds with distinct modes of action. While herbicides are often applied at fixed rates, such approaches do not necessarily correspond to comparable levels of growth inhibition or treatment intensity across herbicides, limiting cross-treatment comparisons (Pfleeger et al., 2011; Metzger et al., 2019). Dose-response analysis provides a robust framework to quantify herbicide effects, enabling the estimation of inhibitory concentrations such as half-maximal inhibitory concentration (IC_50_) and 90-99% inhibitory concentration (IC_90-99_), which represent moderate and severe growth inhibition, respectively (Seefeldt et al., 1995). Defining herbicide-specific treatment concentrations based on these quantitative physiological endpoints facilitate establishing standardized treatment levels for comparable analyses. Although such treatment levels do not necessarily correspond to the same physiological states at individual sampling time points due to differences in herbicide mode of action, they provide a quantitative framework for downstream analyses, as it allows subsequent comparisons across herbicides under standardized treatment intensities.

Transcriptomic studies particularly using RNA-sequencing (RNA-seq), enable high throughput, comprehensive, and time-course monitoring of global gene expression changes following herbicide treatment, capturing both immediate and dynamic transcriptomic responses across diverse biological pathways (Wright et al., 2018; Cechin et al., 2020). Recently, the application of transcriptomics in herbicide research has significantly advanced our understanding of herbicide MOAs and resistance mechanisms by revealing the molecular pathways and networks perturbed by specific herbicides (Duke et al., 2013; Simões et al., 2018; Chen et al., 2024).

However, the interpretation of the transcriptomic data is highly dependent on experimental setup of herbicide doses and exposure duration, which determine the physiological state of the plant at the time of analysis (Serra et al., 2015; Giacomini et al., 2018; Markus et al., 2021). Using graded growth-inhibitory doses obtained from a well-defined physiological state and sampling at strategic time points in RNA-seq has been key to disentangling early MOA associated changes and detoxification from late non-specific damage responses, and to identifying possible resistance mechanisms such as enhanced metabolism (Duke et al., 2013; Sen et al., 2023; Chen et al., 2024). Treatment with IC_50_ at short-term exposure inhibits the primary molecular target and perturbs MOA-associated pathways (Sen et al., 2023). This target inhibition triggers a sublethal, adaptive stress response and induces transient transcriptomic shifts associated with stress perception, detoxification, and cellular defense. Genes involved in xenobiotic detoxification, such as cytochrome P450 (CYP450) monooxygenases, glutathione *S* transferases (GSTs), UDP-glucosyltransferases (UGT), and ATP-binding cassette (ABC) transporters, alongside genes associated with antioxidant production and hormone signaling, are often upregulated in this phase (Baerson et al., 2005; Ramel et al., 2012). These coordinated responses aim to mitigate herbicide-induced damage, enhance cellular repair, and maintain metabolic homeostasis under chemical stress. In contrast, long-term exposure at IC_90–99_ induces transcriptomic profiles related to severe cellular injury and programmed cell death. Key gene types and processes, including proteases and hydrolases, hormonal pathways, autophagy-related genes, and reactive oxygen species (ROS) signaling, have strong induction in this stage (Rowarth et al., 2021; Hopson et al., 2022; Traxler et al., 2023).

Recent transcriptomic studies have increasingly applied RNA-seq for investigating plant responses to herbicide treatments, both in model plant species such as *Arabidopsis thaliana* as well as in various weed species (Patterson et al., 2019; Sen et al., 2023; Chen et al., 2024). However, most of these studies have been focused on examining a single herbicide, which limits the ability to distinguish transcriptional changes directly associated with an herbicide’s primary MOA from broader defense or stress-related responses (Baerson et al., 2005; Duke et al., 2013). Comparative transcriptomic analysis across multiple herbicides with distinct MOAs provides a powerful framework to identify MOA-specific gene expression patterns, as well as conserved defense and detoxification pathways that are commonly activated across treatments (Das et al., 2010; Mesnage et al., 2018). One of the few previous studies on multi-herbicide comparisons was conducted by Das et al. (2010), who used microarrays to identify a set of 101 genes as a “composite transcriptional signature” responsive to five herbicides spanning two distinct MOAs.

Importantly, this gene set not only distinguished transcriptional responses between the two MOAs but also differentiated responses among individual herbicides sharing the same molecular target. While this work provided an early framework for multiple herbicide comparisons, it was performed using microarray technology, involved fewer herbicide MOAs, and focused on expression signatures rather than dose- and exposure time-related dynamics.

In the present study, we integrated physiological and transcriptomic analyses to systematically examine responses in *Arabidopsis* to seven herbicides [2,4-dichlorophenoxyacetic acid (2,4-D), acifluorfen, atrazine, glyphosate, imazethapyr, mesotrione, and paraquat] representing seven different MOAs (**Table 1**). We first conducted dose-response experiments to determine IC_50_ and IC_99_ values for each herbicide based on shoot fresh weight reduction, establishing standardized treatment levels for comparative transcriptomic analyses. We then performed high-resolution RNA-seq under a pairwise design incorporating both sublethal (IC_50_) and lethal (IC_99_) concentrations under short-term (4 h) and long-term (24 h) exposure. This experimental framework enabled systematic comparisons of transcriptional responses to standardized treatment levels across herbicides with different MOAs, while acknowledging that herbicides may not induce the same physiological states at each sampling time because of different MOAs. Our comparative transcriptome analyses across herbicides revealed herbicide-specific signatures, including distinct differentially expressed gene sets involved in specific MOA-associated and convergent broader stress response-related biological effects, all of which were regulated in a treatment- and time-related manner. To our knowledge, this represents the most comprehensive multi-herbicide comparative transcriptomic analysis to date. These findings provide insights into the adaptive and lethal phases of herbicide stress responses, establish a quantitative framework for linking herbicide-specific treatment intensity with underlying molecular responses, highlight transcriptomic signatures that may serve as diagnostic markers for specific herbicides, and facilitate the study of herbicide resistance at the molecular level.

**Table 1:** Herbicide mode of action.

Herbicide name	Herbicide group	Mode of action
2,4-D	4	Growth regulatorsTIR1 auxin receptors (synthetic auxin)
Acifluorfen	14	Cell membrane disrupters- protoporphyrinogen oxidase (PPO) inhibitors
Atrazine	5	Photosystem II inhibitors
Glyphosate	9	Amino acid synthesis inhibitors--EPSP synthase inhibitors (5-enolpyruvylshikimate- 3-phosphate)
Imazethapyr	2	Amino acid synthesis inhibitors--ALS inhibitors (acetolactate synthase)
Mesotrione	27	Pigment inhibitors-HPPD inhibitors (4hydroxyphenylpyruvate dioxygenase)
Paraquat	22	Cell membrane disrupters-- Photosystem I electron diverter

## 2 Materials and methods

### 2.1 Plant materials and growth conditions

All experiments in this study were conducted with wild-type *Arabidopsis thaliana* accession Columbia-0 (*Col-0*). One hundred seeds were uniformly distributed on the soil surface using a 100-pin seeding manifold and guide plate (V&P Scientific, San Diego, CA, USA) to minimize experimental variation. The pot had a soil surface area of approximately 212.5 cm^2^ (12.5 cm x 17 cm), corresponding to an approximate planting density of 0.47 seeds cm^-2^. Seeded trays were sub-irrigated, vernalized for 3 days in dark at 4 °C, then maintained in a growth chamber for ten days at 21 °C during the day and 16 °C during the night (16 h light/8 h dark), with a relative humidity 70-75% and a light intensity of ~150 µE during the light period, provided by cool-white fluorescent lamps (Philips F32T8/TL841/ALTO II, Signify N.V., Eindhoven, Netherlands).

### 2.2 Chemicals

Herbicide chemicals were purchased from the sources listed below and used without further purification. 2,4-dichlorophenoxyacetic acid (2,4-D, PESTANAL, MilliporeSigma Supelco, Supelco Inc., Bellefonte, PA, USA), acifluorfen (purity: 98.8%); atrazine; imazethapyr (purity: 99%); glyphosate; mesotrione (purity: 99.5%) and paraquat CL tetrahydrate (purity: 99.5%) were obtained from Chem Service Inc. (West Chester, PA, USA), now a part of AnalytiChem (Eschborn, Germany). Dimethyl sulfoxide (DMSO), 2-(N-Morpholino)ethanesulfonic acid hydrate (MES, purity ≥99.5%), ethanol and Tween-20 used for herbicide solution preparation were purchased from Sigma-Aldrich (St. Louis, MO, USA).

### 2.3 Herbicide dose-response analysis

Ten-day-old *Arabidopsis* seedlings grown in the growth chamber were subjected to herbicide treatments. To quantitatively characterize herbicide-induced physiological responses, a dose-response experiment was performed to determine the IC_50_ and IC_99_ values of each herbicide that was used for the RNA-seq experiment. For each herbicide, *Arabidopsis* seedlings were exposed to a gradient of herbicide concentrations spanning a broad dynamic range (**Supplementary Table S1**). Each concentration included three independent biological replicates, with 30 seedlings per replicate. Therefore, totally 90 seedlings were evaluated for each concentration. After four days of exposure, shoots (above-soil tissues) from the 30 seedlings per replicate were harvested, and total fresh weight (g) was recorded. IC_50_ and IC_99_ values were defined as the herbicide concentrations required to reduce total shoot fresh weight by 50% and 99% respectively, relative to the untreated control.

IC_50_ and IC_99_ values used for RNA-seq experiments were initially estimated using Xlfit (a Microsoft Excel add-in). To validate these estimates, the currently available raw dose-response data were reanalyzed using a four-parameter log-logistic model (LL.4) implemented in the R package *drc* (version 3.0-1) (Ritz et al., 2015). Dose-response curves were plotted with a logarithmic concentration scale against shoot fresh weight, and IC_50_ and IC_99_ were estimated from the fitted models. The IC_50_ estimates obtained from the original Xlfit analysis and the retrospective R-based *drc* reanalysis were generally consistent with only minor variation. In contrast, the IC_99_ values varied between the two fitting models, reflecting the greater uncertainty associated with estimating an extremely high dose. Nevertheless, the original Xlfit-estimated IC_99_ values were contained within the corresponding 95% confidence intervals estimated by the *drc* model (**Supplementary Table S2**).

For the RNA-seq experiments, treatment solutions corresponding to the Xlfit-estimated IC_50_ and IC_99_ concentrations were prepared individually. The herbicide treatment solutions were prepared using the following solvents: ethanol for 2,4-D and acifluorfen; water for glyphosate and paraquat; DMSO for imazethapyr and atrazine; and MES buffer for mesotrione. For each herbicide, the corresponding mock control was prepared without the active ingredient and contained the same solvent composition and 0.1% Tween-20 as the corresponding herbicide treatment solution. The specific stock concentrations, solvent volumes, and preparation procedures are provided in **Supplementary Table S3**. The herbicide mock control and treatment solutions were applied using an automated spray cabinet (Greenhouse Spray Chamber; Generation III) with an 8002E TeeJet spray nozzle and boom height of 41 cm, providing a diluent spray volume equivalent to 360 L ha^-1^.

### 2.4 RNA extraction, cDNA library construction, and sequencing

Three independent biological replicates of herbicide-treated *Arabidopsis* seedlings and mock-treated controls were harvested at 4 h and 24 h post-treatment. For example, for the 2,4-D experiment, ten-day-old plants treated with IC_50_ levels were harvested at 4 h and 24 h posttreatment. Plants were also treated with IC_99_ levels of 2,4-D and were harvested at 4 h and 24 h post-treatment identically. For each time point (4 h and 24 h), a corresponding set of mock treated control samples in biological triplicates was included and served as the control for both IC_50_ and IC_99_ treatments. Thus, there were a total of 18 samples generated per herbicide (2 concentrations x 2 time points x 3 biological replicates + 6 control samples). The only exception was mesotrione IC_50_ at 4 h treatment, which only included two biological replicates due to limited RNA availability. Consequently, there were 125 biological samples (18 biological samples x 7 herbicides – 1 mesotrione biological sample) in total for RNA-seq.

Total RNAs for use in RNA-seq experiments were isolated using the RNeasy Plant Mini Kit (Qiagen Inc., Valencia, CA, USA) following the manufacturer’s instructions. RNA concentration and purity were determined spectrophotometrically using the Nanodrop 2000 instrument (Thermo Fisher Scientific, Waltham, MA, USA). The integrity of RNA samples was evaluated on the Agilent 2100 Bioanalyzer (Agilent Technologies Inc., Santa Clara, CA, USA).

RNA-seq library preparation and sequencing were performed in our laboratory. Sequencing libraries were generated with 300 ng to 1 µg of total RNA using the TruSeq Stranded mRNA Sample Preparation Kit (Illumina, San Diego, CA, USA). Each of the two biological RNA samples of mesotrione IC_50_ at 4 h treatment was sequenced twice, resulting in a total of 127 sequencing libraries. The libraries were then assessed for size and purity using the Agilent 2100 Bioanalyzer and quantified by RT-qPCR with a KAPA Biosystems Library Quantitation kit (Roche, Pleasanton, CA, USA). Libraries were normalized, pooled, and diluted to a loading concentration of 1.8 pM. RNA sequencing was performed in our laboratory using an Illumina NextSeq 500 instrument, producing 76 bp paired-end reads, with approximately 11 million reads/per lane distributed evenly across the four flow cell lanes, and all run metrics, including Q-Score distributions, cluster densities, and total sequence yields, were within the recommended parameters.

### 2.5 Transcriptomic analysis

RNA-seq data were analyzed using the Qiagen CLC Genomics Workbench (CLCGWB) software (version 23.0.2, Qiagen, Hilden, Germany). Sequencing reads were mapped to the *Arabidopsis* reference genome (Araport11), using default parameters with a strand-specific alignment protocol. The mapping report indicated that all metrics fell within the recommended parameters, with > 90% of the reads per sample mapping to the reference genome. Detailed sequencing and mapping statistics for each RNA-seq library are provided in **Supplementary Table S4**.

Differentially expressed genes (DEGs) were identified using pairwise comparisons between each treatment and its corresponding mock control (e.g., IC_50__4 h vs CK_4 h, IC_50__24 h vs CK_24 h, IC_99__4 h vs CK_4 h, IC_99__24 h vs CK_24 h). No formal dose x time interaction model was applied. Consequently, temporal and dose-related transcriptional patterns described in this study were based on comparisons of individual treatment condition against its corresponding control rather than statistical interaction tests. DEGs were obtained using the “Differential Expression for RNA-Seq” tool in CLCGWB software. Genes with a fold-change of ≥ 2 or ≤ -2, a Bonferroni-corrected *p* value of < 0.01, and a Maximum Group Mean TPM (Transcripts Per Million) of > 5 were considered differentially expressed. The list of DEGs identified in each statistical comparison under each treatment is shown in **Supplementary Table S5**. Principal Component Analysis (PCA) plot (2D) was generated in CLCGWB using normalized log CPM values (Counts per Million) to assess the relatedness among the samples. UpSet plots were generated by inputting complete DEG sets from each herbicide treatment on the Intervene Shiny App (https://asntech.shinyapps.io/intervene/) to visualize the intersections and overlaps across herbicide treatments. Because the Intervene Shiny app does not support direct export of gene lists for each intersection, Venn diagrams (https://bioinformatics.psb.ugent.be/webtools/Venn/) were generated separately to extract herbicide unique gene sets (**Supplementary Table S6**) for each condition.

Gene Ontology (GO) enrichment analysis was performed using the R package *clusterProfiler* (Yu et al., 2012). DEGs list corresponding to different herbicide treatment were imported and analyzed separately for enrichment of Biological Process (BP), Molecular Function (MF), and Cellular Component (CC) categories. Gene identifiers were based on *Arabidopsis* TAIR locus IDs, and annotations were retrieved using the *org.At.tair.db* database. Enrichment analysis was conducted using an over-representation approach with a significance threshold of adjusted *p*-value < 0.05. Results were further compared and visualized across herbicides within the same condition using *compareCluster* function with *enrichGO* built in *clusterProfiler*, enabling identification of shared and herbicide-specific top functional GO terms. Comparative visualization focused on BP ontology. To reduce semantic redundancy, enriched BP terms were simplified independently for each herbicide using the *simplify()* function in *clusterProfiler*. Pairwise GO-term similarity was calculated using the Wang semantic similarity method (Wang et al., 2007), with a similarity cutoff of 0.60. The five top-ranked simplified terms for each herbicide were selected primarily by adjusted *p*-value, with fold enrichment used as a secondary ranking criterion. These selected terms of each herbicide were used for comparative dot-plot visualization (**Figures 1**–**4**), and each term was displayed for all herbicides in which it was significantly enriched. Dot size represents fold enrichment, calculated as the ratio of the observed gene proportion to the corresponding background proportion, whereas dot color represents the adjusted *p*-value. Complete unsimplified GO enrichment results are provided in **Supplementary Table S7**.

**Figure 1:**
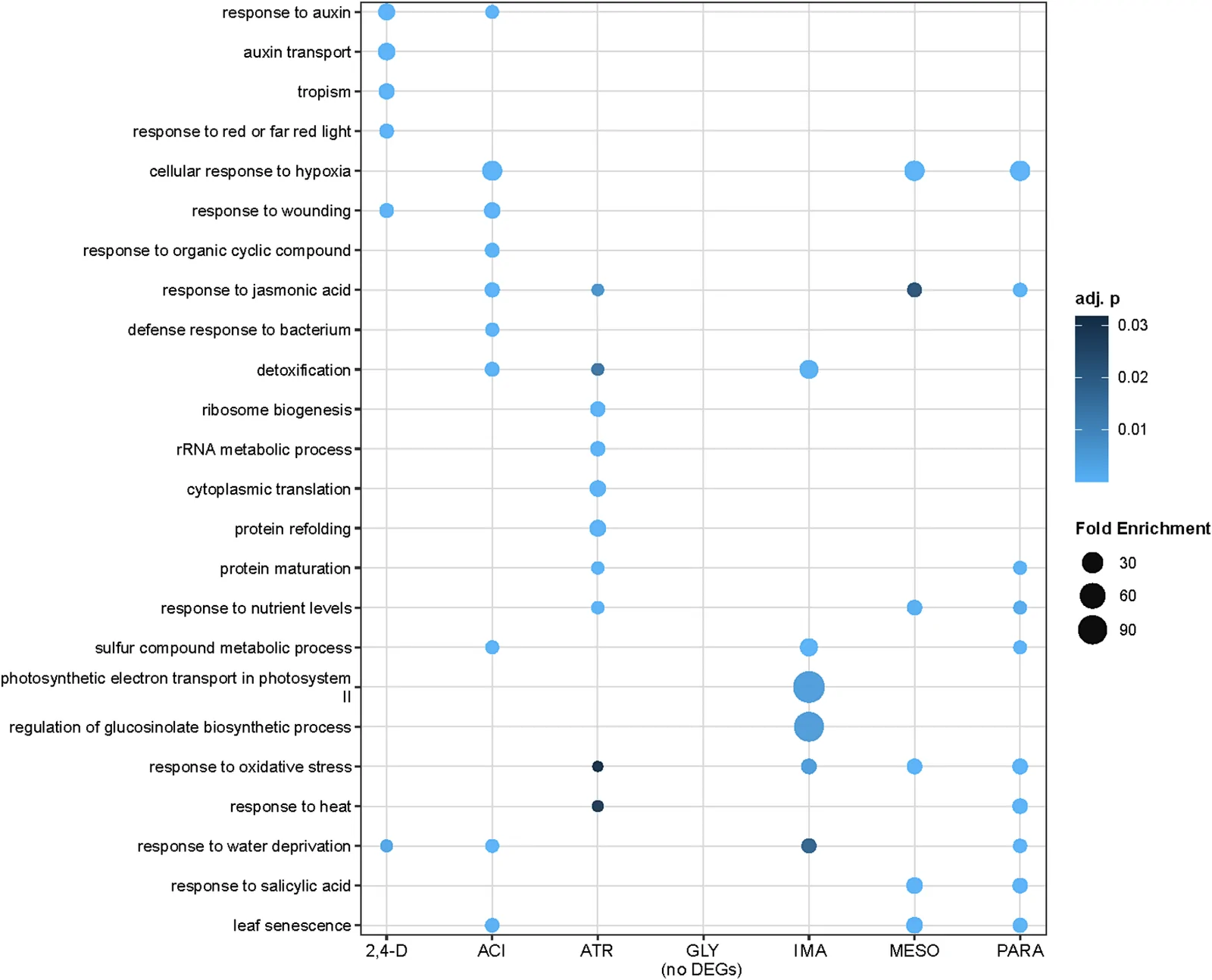
Comparative Gene Ontology (GO) enrichment analysis of differentially expressed genes across herbicide treatments at IC_50__4 h. Comparative visualization focused on the BP (biological process) ontology. Top five enriched GO terms per herbicide were selected after semantic simplication, compiled, and displayed together. Enrichment analysis was performed using R package *clusterProfiler*. The top GO terms were selected based on statistical significance. Color scale represents enrichment significance (adjusted *p* value), and dot size indicates the fold enrichment of genes associated with each term.2,4-D: 2,4-dichlorophenoxyacetic acid, ACI: acifluorfen, ATR: atrazine, GLY: glyphosate, IMA: imazethapyr, MESO: mesotrione, and PARA: paraquat. GLY was not analyzed because no DEGs were identified under our RNA-seq analysis parameters. The complete unsimplified GO terms for each herbicide are provided in **Supplementary Table S7**.

**Figure 2:**
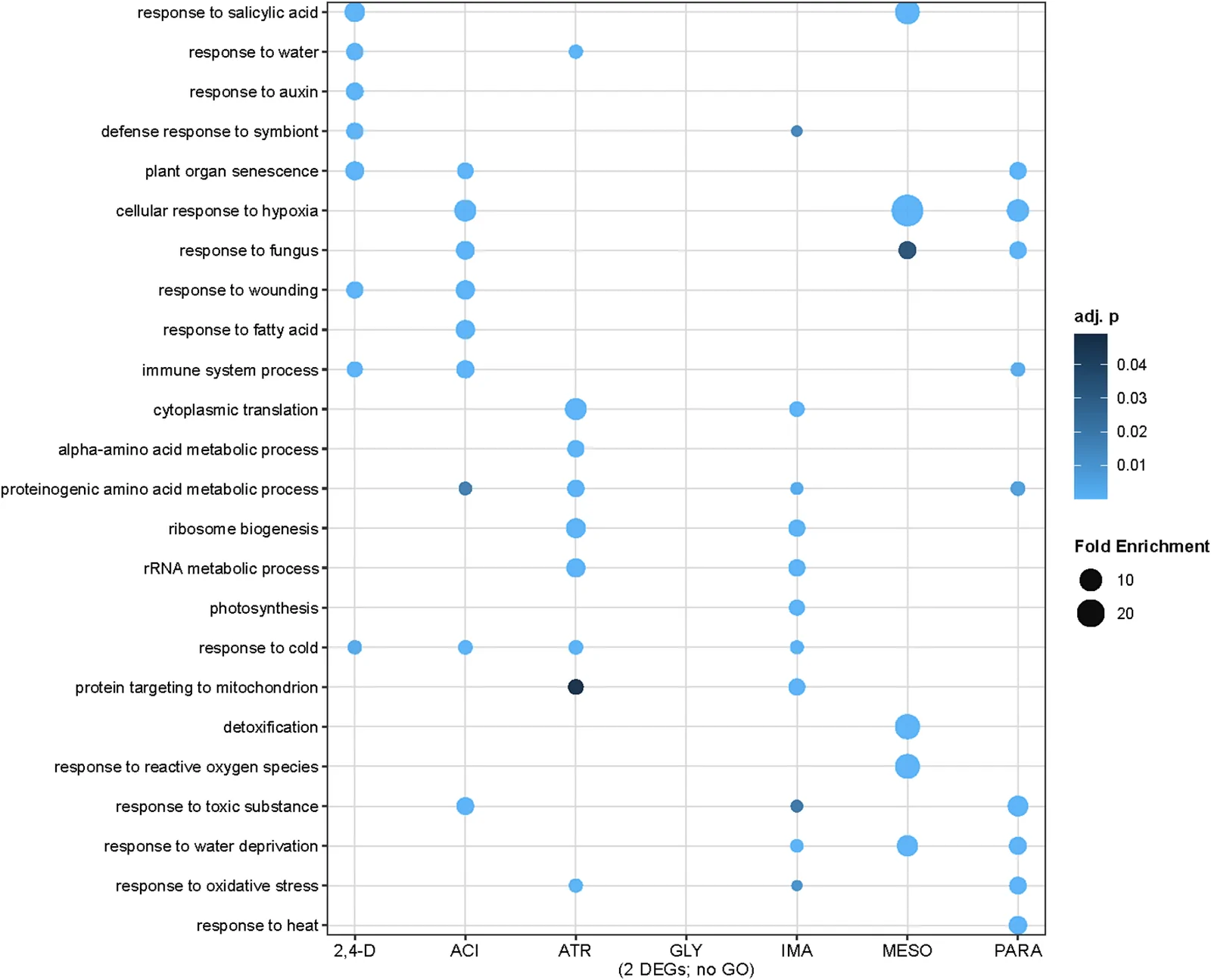
Comparative Gene Ontology (GO) enrichment analysis of differentially expressed genes across herbicide treatments at IC_50__24 h. Comparative visualization focused on the BP ontology. Top five enriched GO terms per herbicide are selected after semantic simplication, compiled, and displayed together. Enrichment analysis was performed using R package *clusterProfiler*. The top GO terms were selected based on statistical significance. Color scale represents enrichment significance (adjusted *p* value), and dot size indicates the fold enrichment of genes associated with each term. GLY was not analyzed because only two DEGs were identified under our RNA-seq analysis parameters. The complete unsimplified GO terms for each herbicide are provided in **Supplementary Table S7**.

**Figure 3:**
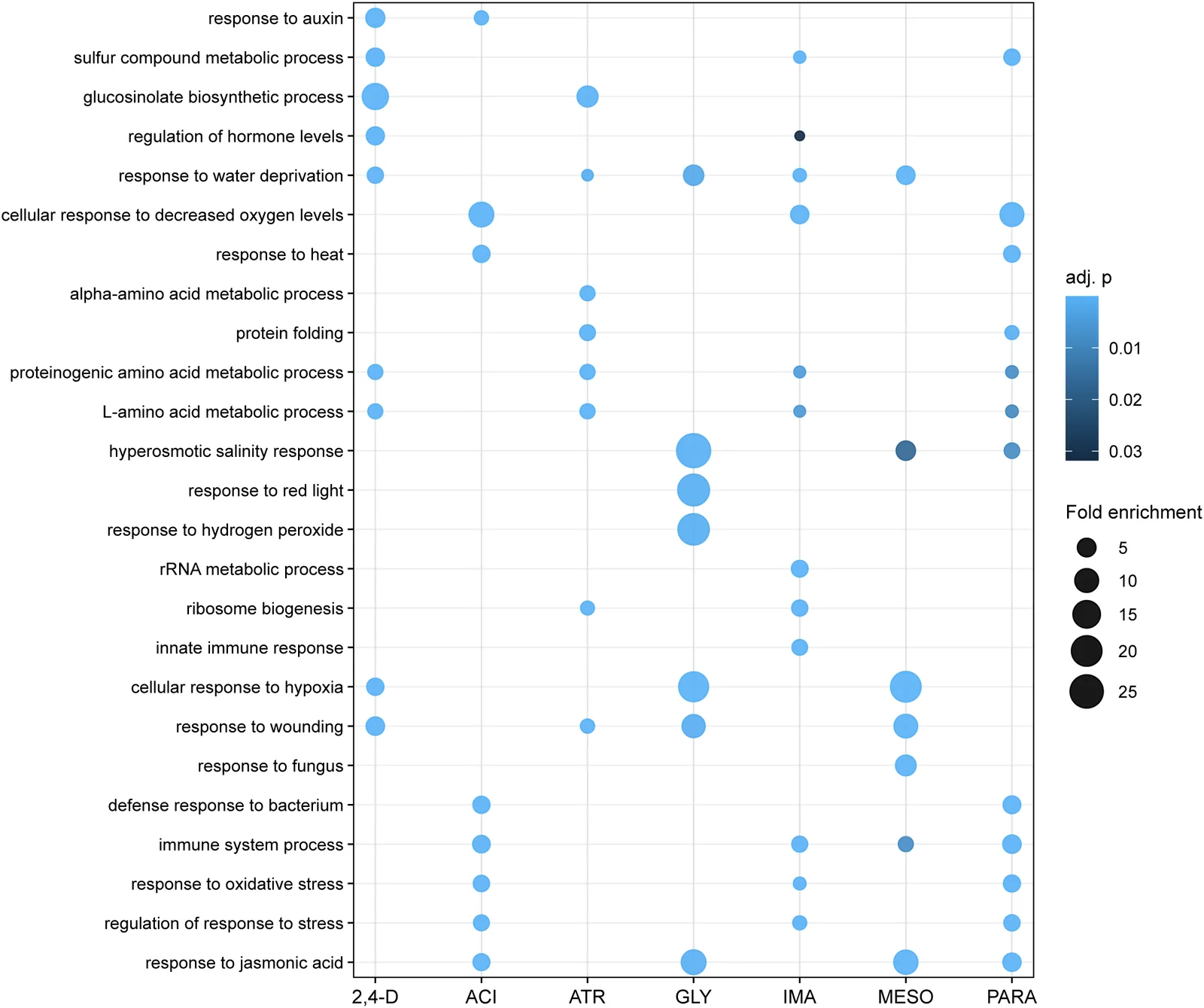
Comparative Gene Ontology (GO) enrichment analysis of differentially expressed genes across herbicide treatments at IC_99__4 h. Comparative visualization focused on the BP ontology. Top five enriched GO terms per herbicide are selected after semantic simplication, compiled, and displayed together. Enrichment analysis was performed using R package *clusterProfiler*. The top GO terms were selected based on statistical significance. Color scale represents enrichment significance (adjusted *p* value), and dot size indicates the fold enrichment of genes associated with each term. The complete unsimplified GO terms for each herbicide are provided in **Supplementary Table S7**.

**Figure 4:**
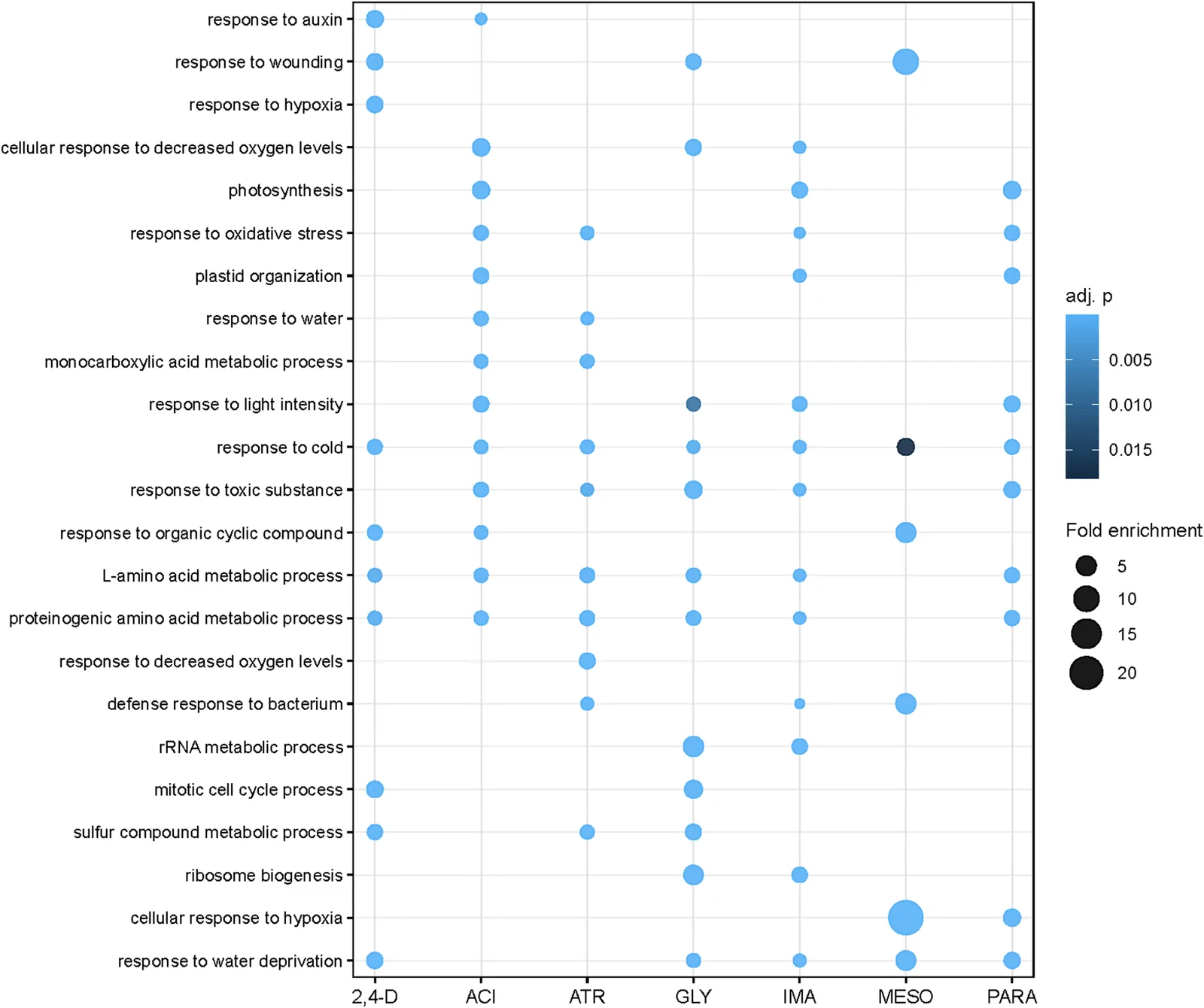
Comparative Gene Ontology (GO) enrichment analysis of differentially expressed genes across herbicide treatments at IC_99__24 h. Comparative visualization focused on the BP ontology. Top five enriched GO terms per herbicide are selected after semantic simplication, compiled, and displayed together. Enrichment analysis was performed using R package *clusterProfiler*. The top GO terms were selected based on statistical significance. Color scale represents enrichment significance (adjusted *p* value), and dot size indicates the fold enrichment of genes associated with each term. The complete unsimplified GO terms for each herbicide are provided in **Supplementary Table S7**.

Hierarchical clustering was performed on unique DEGs identified under each treatment condition. For each herbicide, the log_2_ fold change (log_2_FC) values of unique DEGs across four treatment conditions (IC_50__4 h, IC_50__24 h, IC_99__4 h and IC_99__24 h) were used as input. Data were organized into a matrix with genes listed as rows and treatment conditions listed as columns. Rows were subjected to clustering while the column order was fixed, and no row-wise scaling or *Z*-score transformation was applied. Genes with missing log_2_FC values in any treatment condition were excluded before clustering, which excluded 13 genes for acifluorfen and 3 genes for imazethapyr, while all genes were retained for the other herbicides (**Supplementary Table S8**). Hierarchical clustering was performed in R using Euclidean distance and Ward’s minimum variance method (ward.D2) to group genes (Murtagh and Legendre, 2014) based on similarity in expression patterns. The number of clusters was evaluated separately for each herbicide over *k* = 2-12. For each *k*, the total within-cluster sum of squares (WSS) and average silhouette width were calculated. Elbow plots were generated for *k* = 1-12, and the average silhouette widths were calculated for *k* = 2–12 using the same Euclidean-distance matrix. When the elbow and silhouette analysis identified different *k* values, cluster number was selected by jointly considering changes in WSS, average silhouette width, cluster size distribution and biological interpretability (e.g., whether additional cluster represented distinct expression profiles) (Handl et al., 2005; Henning, 2015; Batool and Henning, 2021). Based on these assessments, *k* = 3, 4, 2, 2, 3, 2 and 4 were selected for 2,4-D, acifluorfen, atrazine, glyphosate, imazethapyr, mesotrione, and paraquat, respectively. The cluster-selection plots are provided in **Supplementary Figures S1, S2** and the numerical results to support those plots are provided in **Supplementary Table S9**. **Supplementary Table S8** summarizes the cluster number selection process for each herbicide, including number of genes analyzed, elbow *k*, maximum silhouette *k*, final selected *k* and selection rationale.

Cluster-specific GO-BP enrichment was analyzed separately for each hierarchical gene cluster using *enrichGO()* in *clusterProfiler*, with the same TAIR annotation and statistical thresholds described above. Complete unsimplified GO-BP enrichment results are provided in **Supplementary Table S10**. For visualization, redundant terms were reduced within each cluster using *simplify()* function, a cutoff of 0.60, and the term with the smallest adjusted *p* value retained as the representative. The top ten significant simplified terms per cluster were displayed for each herbicide in **Supplementary Figures S3–S9**. Dot position, size, and color represent -log_10_ (adjusted *p* value), gene count, and fold enrichment, respectively. Cluster numbers correspond to the top-to-bottom position in the heatmaps.

The RNA-seq raw data and processed data described in this study are accessible through accession NO. GSE327091 at NCBI’s Gene Expression Omnibus database.

### 2.6 RT-qPCR validation of RNA-seq data

Real-time quantitative PCR (RT-qPCR) was performed to validate gene expression patterns identified by RNA-seq. Representative genes were selected for each herbicide treatment, including MOA-associated genes, cluster-representative genes reflecting expression dynamics, and genes involved in shared functional categories such as CYP450s, UGTs and ABC transporters (**Supplementary Table S11**). Total RNA samples used for RNA-seq were also used for RT-qPCR analysis. First-strand cDNA was synthesized from DNase-treated RNA using LunaScript^®^ RT SuperMix Kit (New England Biolabs, Ipswich, MA, USA) following manufacturer’s instructions. Custom DNA primers were synthesized by Sigma-Aldrich (St. Louis, MO, USA) (**Supplementary Table S12**) and used for amplification with SYBR^™^ Green Universal Master Mix on an Applied Biosystems real-time PCR system (Thermo Fisher Scientific, Waltham, MA, USA). *Arabidopsis PP2A* gene (AT1G13320) was used as the internal reference gene (Czechowski et al., 2005). RT-qPCR validation was performed using three biological replicates for most herbicides under each treatment condition (CK_4 h, IC_50__4 h, IC_99__4 h, CK_24 h, IC_50__24 h, and IC_99__24 h), with three technical replicates per biological replicate. Due to limited RNA sample availability, paraquat and mesotrione treatments were validated in single biological replicate at 4 h at both dosages, whereas both treatments at 24 h were analyzed in biological triplicate. Ct values were exported and analyzed manually. Technical replicates were averaged prior to the downstream analysis and relative expression levels were calculated using the 2^-ΔΔCt^ method. ΔCt values were obtained by normalizing target gene Ct values to the internal reference gene *PP2A*, and ΔΔCt values were calculated relative to the corresponding control within each time point (4 h or 24 h). Statistical analyses were performed using GraphPad Prism version 11.0.0 (GraphPad Software, Boston, MA, USA) on 2^-ΔΔCt^ values from three technical replicates from each biological replicate using one-way or two-way ANOVA as appropriate. Expression levels are presented as mean ± standard deviation (SD). Statistical significance was defined as *p* < 0.05. Significance levels are indicated in figures as follows: **** *p* < 0.0001, *** *p* < 0.001, ** *p* < 0.01, * *p* < 0.05, ns (not significant): *p* > 0.05.

## 3 Results

### 3.1 Dose-response analysis revealed divergent physiological sensitivity to herbicides

Herbicides act through different MOAs, and a single plant species can display different injury symptoms depending on the compound applied. The seven herbicides used in this study represent different herbicide groups (Global Herbicide Classification: https://hracglobal.com) with well-characterized MOAs. These modes of action span distinct mechanisms, including growth regulator (2,4-D), cell membrane disrupters (acifluorfen and paraquat), photosystem inhibitor (atrazine), amino acid synthesis inhibitors (glyphosate and imazethapyr), and pigment inhibitor (mesotrione) (**Table 1**).

To quantitatively determine the different responses of seedlings to each herbicide, dose-response experiments were performed by spraying a range of herbicide concentrations on ten-day-old *Arabidopsis* seedlings grown in pots. Growth inhibition was measured at four days post-treatment as detailed in the Methods section. Representative dose-response phenotypes under the treatment of a serial concentrations of imazethapyr are shown in **Figure 5A**, illustrating the progressive effects of increasing concentrations on plant growth.

**Figure 5:**
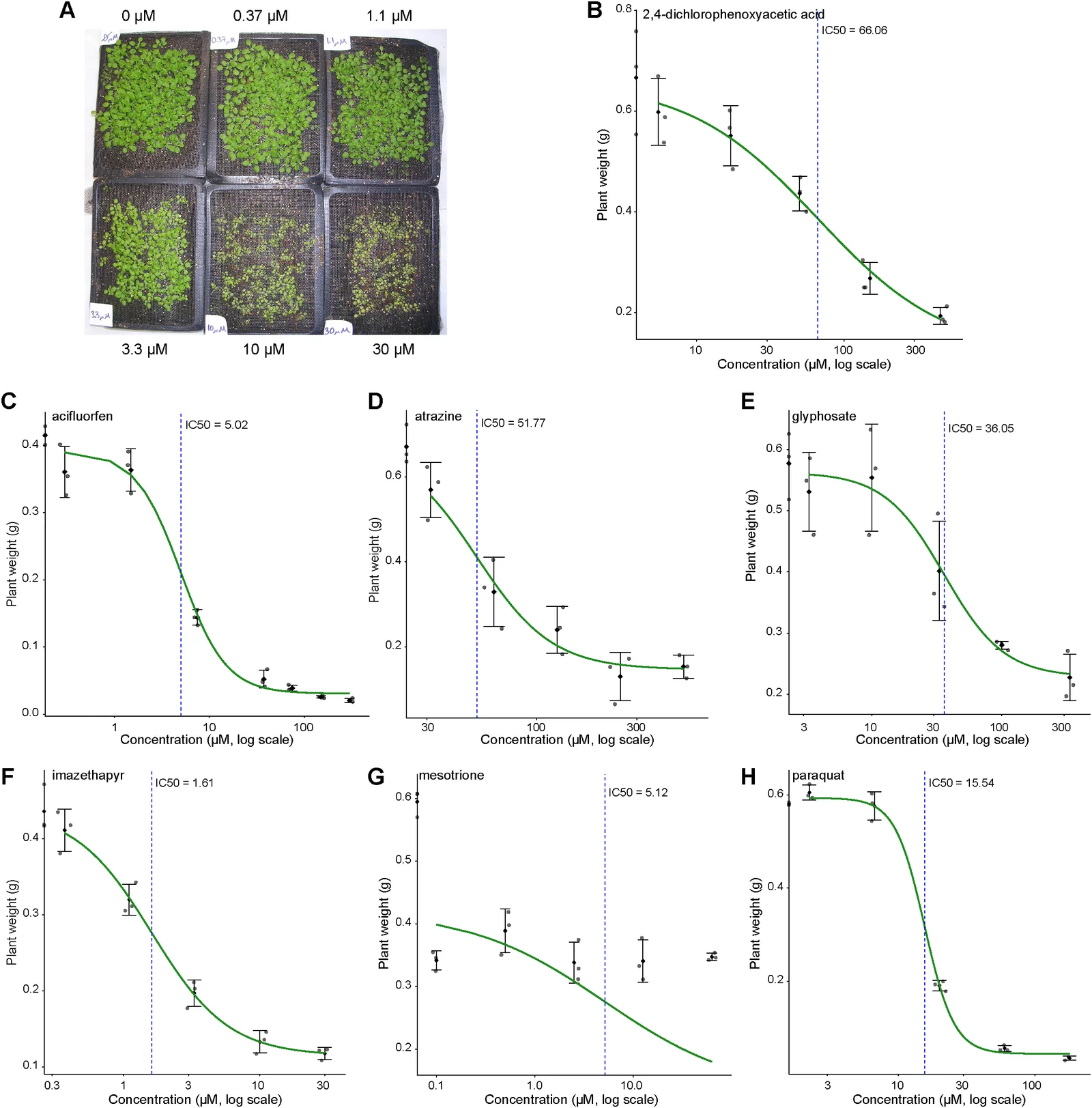
Representative physiological effects of herbicide treatments and dose-response retrospective reanalysis. **(A)** Representative phenotypic responses of 10-day-old plants treated with a series of concentrations of imazethapyr (0; 0.37; 1.1; 3.3; 10 and 30 μM), showing concentration dependent growth inhibition. **(B–H)** Dose-response curves for the seven herbicides generated by retrospective reanalysis of the currently available dose-response data using the R package *drc*. Curves were fitted based on shoot fresh weight (g) of 10-day-old *Arabidopsis* seedlings in pots four days after treatment. Fresh weights were measured from 30 shoots per biological replicate across three independent biological replicates, for a total of 90 shoots per concentration. The original herbicide concentrations used for the RNA-seq experiments were established previously using Xlfit, whereas the *drc*-based analysis is performed in this study to confirm a reproducible statistical framework for the Xlfit-determined IC_50_ and IC_99_ values and is not to redefine the treatment concentrations used in the RNA-seq experiment. The comparison between the Xlfit-determined IC_50_ and IC_99_ values and the estimates obtained from retrospective reanalysis using *drc* is summarized in **Supplementary Table S2**. Dashed lines indicate the model-derived IC_50_ estimates from the retrospective reanalysis using *drc*.

The dose-response experiments described above were performed to establish the herbicide treatment conditions for the transcriptomic study. The resulting data were previously analyzed using Xlfit to determine the IC_50_ and IC_99_ values ultimately used for the RNA-seq experiments. Because the original Xlfit fitting models were no longer available when preparing the present study, the currently available previous raw dose-response data were retrospectively reanalyzed using the R package *drc*. The retrospective reanalysis using *drc* aimed to confirm a reproducible statistical framework for the Xlfit-determined IC_50_ and IC_99_ values and should not be interpreted as redefining the treatment concentrations used in the RNA-seq experiment. The dose-response curves generated using *drc* for all seven herbicides are presented in **Figures 5B–H**. The comparison between the Xlfit-determined IC_50_ and IC_99_ values and the estimates obtained from retrospective reanalysis using *drc* is summarized in **Supplementary Table S2**. Overall, the IC_50_ values obtained from both fitting approaches were generally consistent while greater differences were observed for the IC_99_ values. This difference was particularly evident for 2,4-D and mesotrione due to the tested concentration ranges did not fully capture the upper asymptote of the dose-response curves, requiring extrapolation of the IC_99_ estimates beyond the observed data (**Figures 5B,G**). Nevertheless, the Xlfit-determined IC_99_ values fell within the corresponding 95% confidence intervals estimated by the *drc* model for all seven herbicides, suggesting that the previous Xlfit-determined treatment concentrations were statistically consistent with the uncertainty associated with *drc* reanalysis, although the IC_99_ 95% confidence intervals exhibited broad range, indicating a limited precision of estimates, particularly for 2,4-D and mesotrione (**Supplementary Table S2**).

In addition, the herbicide treatment concentrations used for RNA-seq experiment were estimated by dose-response analyses based on shoot fresh weight reduction, and the IC_50_ and IC_99_ represented approximately 50% and 99% growth inhibition, respectively. Thus, the comparative transcriptomic framework was designed at the physiologically defined treatment level representing moderate (IC_50_) and severe (IC_99_) herbicide effects (**Supplementary Figure S10**) rather than the exact precision of IC_50_ and IC_99_ estimates. Although the IC_99_ estimates for 2,4-D and mesotrione are associated with greater uncertainty due to the upper asymptote being not fully captured, the estimates were within the severe herbicide effect region predicted from the dose-response curve. Thus, transcriptomic responses at these concentrations for 2,4-D and mesotrione should be interpreted as responses to a model-derived severe herbicide treatment instead of being interpreted as responses to an exact empirical IC_99_ concentration.

Notably, the seven herbicides exhibited significant variations in potency, as reflected by differences in their dose-response profiles. The steepness of the dose-response curve reflected the rate at which physiological effects increased with concentrations (Milosevic and Knezevic, 2024). Steeper slopes for acifluorfen and paraquat (**Figures 5C, H**) indicated a faster transition from low to high inhibition effects across a narrow concentration range. In contrast, the shallow slopes implied a broader concentration range separating IC_50_ and IC_99_, such as 2,4-D, glyphosate and mesotrione (**Figures 5B, E, G**) in this study. Representative physiological effects are shown in **Supplementary Figure S10**, where *Arabidopsis* seedlings treated at estimated IC_50_ and IC_99_ concentrations display varying degrees of herbicide effect, consistent with moderate and severe growth inhibition, respectively. Imazethapyr and atrazine were able to inhibit the growth of nearly half seedlings at micromolar concentrations (**Supplementary Figures S10B, C**; **Supplementary Table S2**), whereas 2,4-D required notably higher concentrations to achieve similar levels of inhibition (**Supplementary Figure S10A**; **Supplementary Table S2**). Such variations highlighted the selected herbicides differed markedly in their physiological impact on *Arabidopsis*. Therefore, it is essential to empirically determine IC values for each compound rather than relying on nominal application rates, as this enables the establishment of standardized herbicide treatment intensities that were determined from moderate and severe growth inhibition, and provides a robust foundation for downstream comparative transcriptomic analyses.

### 3.2 Herbicide treatments triggered distinct global transcriptional profiles under defined physiological conditions

Based on these physiologically defined inhibitory concentrations, RNA-seq was performed to investigate the transcriptional responses of *Arabidopsis* to herbicide treatments under comparable levels of treatment intensity. Ten-day-old seedlings were treated with seven herbicides under a pairwise experimental design (**Figure 6A**). For each herbicide, treatments were applied at two concentrations (IC_50_ and IC_99_), and seedlings were harvested at two time points (4 h and 24 h) for each concentration. In parallel, mock-treated control samples were collected at both time points. This design enabled a comprehensive comparison of dose- and time-related transcriptional responses across the seven herbicides with different MOAs.

**Figure 6:**
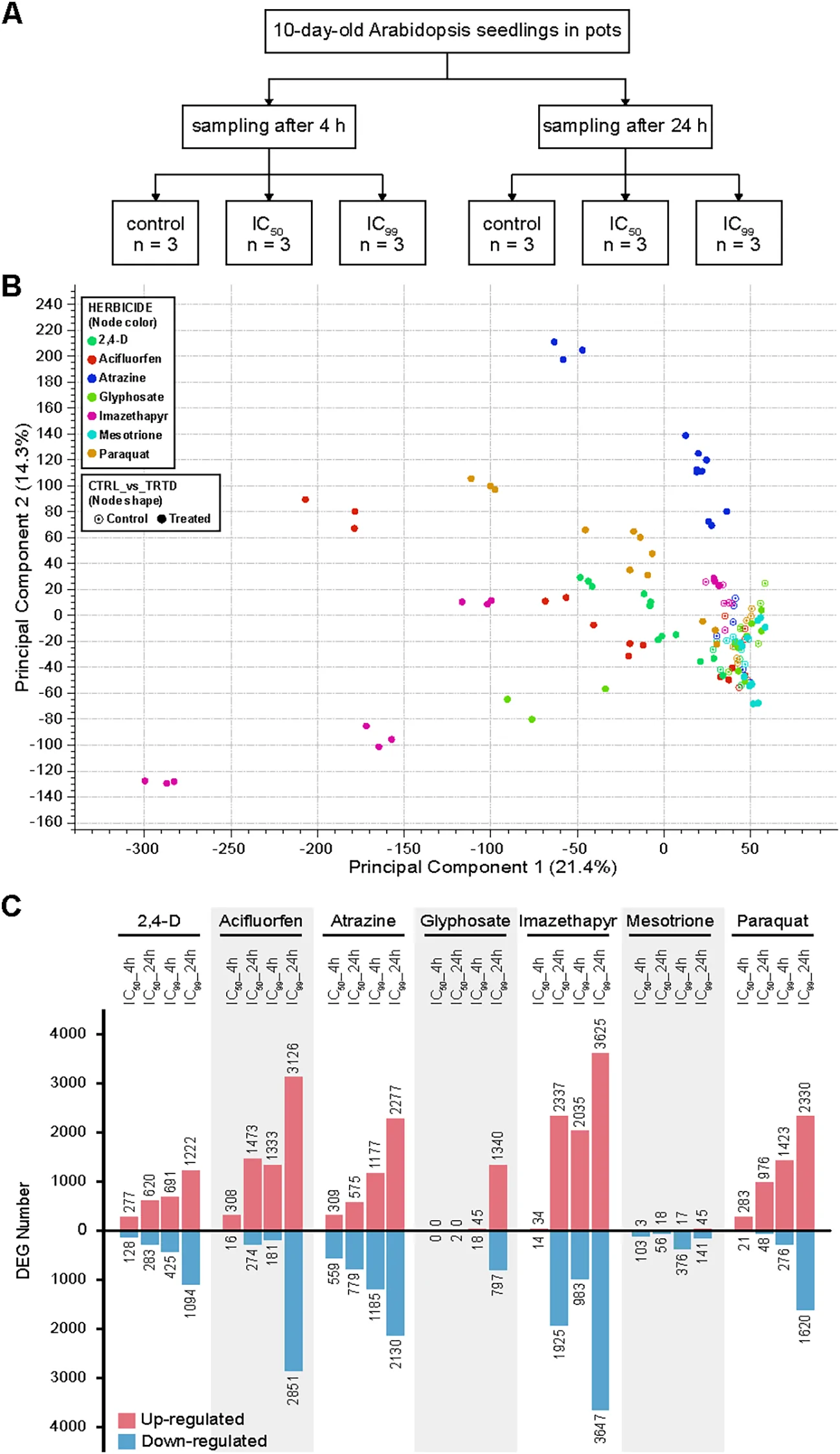
Experimental design and global transcriptomic responses to herbicide treatments. **(A)** Schematic representation of experimental design. *Arabidopsis* seedlings were treated with seven herbicides at two time points (4 h and 24 h) and two dosages (IC_50_ and IC_99_), with three biological replicates per treatment and control at each time point, followed by RNA-seq analysis. **(B)** Principal component analysis showing the variations and clustering of samples across seven herbicide treatments, time points and dosages. Samples are color-coded by herbicide treatment and shaped by group (control vs. treated). **(C)** Summary of total differentially expressed genes identified for each herbicide under the indicated conditions. Bars represent the number of upregulated (red) and downregulated (blue) genes based on the defined statistical thresholds described in Methods section.

The RNA-seq experiment generated a high-quality dataset, with each library yielding robust average read counts and higher than 90% of the reads uniquely mapping to the reference genome (Araport11) (**Supplementary Table S4**). Principal Component Analysis (PCA) performed on normalized gene expression data using CLC Genomics Workbench showed global transcriptional variations across treatments (**Figure 6B**). The clear separation between herbicide-treated and control samples suggested strong transcriptional responses to herbicide exposure. Within each herbicide, gene expression changes from different treatments (IC_50__4 h, IC_50__24 h, IC_99__4 h, IC_99__24 h) separate from each other, with IC_99_ at 24 h exposure showing the greatest divergence from other conditions. Notably, some herbicides, such as imazethapyr, induced distinct transcriptional profiles across all treatments, while others, such as mesotrione, did not trigger massive gene expression changes under all treatments, suggesting variations in transcriptional plasticity in early response to different herbicides (Stern et al., 2007; Bei et al., 2024).

Having an overview of the global variation in transcriptomic responses across all treatments, we next quantified the differentially expressed genes (DEGs) for each herbicide treatment compared to its corresponding control. Generally, the number of DEGs increased with both herbicide dose and exposure duration (**Figure 6C**; **Supplementary Table S5**; **Supplementary Table S13A**). IC_99_ treatments generally induced a larger number of transcriptional changes compared to IC_50_ treatments for each herbicide, and 24 h exposure typically triggered more DEGs than the 4 h exposure under certain concentration treatment. At the individual herbicide level, distinct trends emerged. For 2,4-D, atrazine, and paraquat, an overall trend of increasing DEG numbers was observed with increasing treatment concentration and longer exposure time, while for acifluorfen and imazethapyr, especially imazethapyr, relatively limited transcriptional changes were induced at IC_50__4 h, but widespread transcriptomic changes were observed at IC_50__24 h, IC_99__4 h and IC_99__24 h. Interestingly, another two herbicides showed noticeably different transcriptional responses. Mesotrione exhibited relatively limited DEGs across all four treatment conditions, indicating comparatively limited transcriptional responses under the time frame and concentration examined. Glyphosate elicited almost no transcriptional changes at IC_50_ under either 4 h or 24 h exposure, and a small number of genes were induced at IC_99_ under 4 h exposure. However, a relatively more pronounced transcriptional response was observed at IC_99_ at 24 h exposure (**Figure 6C**). These observations indicated that different herbicides exhibited distinct early transcriptional response patterns under comparable levels of growth inhibition.

In addition, to evaluate potential transcriptional drift between the sampling period, mock-treated control samples collected at 4 h and 24 h were compared for each herbicide. Using the same RNA-seq analysis method and the same statistical criteria applied in the herbicide-responsive DEGs analyses, RNA-seq differential expression analysis revealed temporal transcriptional changes between the two mock controls (**Supplementary Table S14**), with top GO enrichment terms primarily associated with circadian rhythm and photosynthesis (**Supplementary Figures S11A–G**), which was consistent with previous study on temporal regulation in *Arabidopsis* (Yang et al., 2020). To further determine whether these temporal changes confounded our interpretation of herbicide responses, we compared the mock control DEGs with those identified in the corresponding IC_99__24 h versus mock_24 h comparisons and overlapped DEGs numbers were revealed for each herbicide. Six of the seven herbicides only had fewer than 13% of the herbicide-responsive DEGs overlapped with the mock control DEGs (**Supplementary Table S14**). Although mesotrione exhibited a higher overlap (19.4%), most of its herbicide responsive DEGs (80.6%) at IC_99__24 h were associated with stress-responsive processes instead of circadian, light or photosynthesis regulation (**Supplementary Figure S11H**). Collectively, this analysis indicated that although temporal transcriptional changes happened between mock control at 4 h and 24 h, they primarily reflected normal circadian and diurnal regulation and did not significantly confound the herbicide-induced transcriptional responses.

### 3.3 Comparative functional analysis identified treatment-associated biological responses

To systematically compare functional responses underlying the observed transcriptomic changes across the seven herbicides, Gene Ontology (GO) enrichment analysis on complete DEGs from each herbicide treatment was performed. This analysis identified different response patterns across treatment conditions.

#### 3.3.1 Early low-dose treatment reflected herbicide primary-target-associated biological pathways

At IC_50__4 h, the top functional responses were largely herbicide-specific with minimal overlaps across treatments as shown in **Figure 1**. As a synthetic auxin, 2,4-D mimics the natural plant hormone indole-3-acetic acid (IAA). It binds to auxin receptor complexes TIR1/AFB, leading to deregulated auxin signaling and uncontrolled gene expression, which results in abnormal cell elongation and ultimately plant death. At IC_50__4 h, 2,4-D was predominantly enriched in multiple auxin-related processes, with additional top-ranked GO terms related to tropism, responses to light, wounding and water deprivation. Besides, it induced ethylene and brassinosteroid signaling pathways as well as regulation of hormone level and growth (**Supplementary Table S7**), which was consistent with the fact that 2,4-D could not be metabolized as quickly as natural auxins, leading to its accumulation that triggered the rapid overproduction of other hormones such as ethylene and unregulated growth (Hansen and Grossmann, 2000; Pazmiño et al., 2012; Xu et al., 2015). Acifluorfen is a protoporphyrinogen oxidase (PPO) inhibitor that targets the PPO enzyme involved in chlorophyll and heme biosynthesis. It binds to PPO active site, leading to accumulation of protoporphyrin IX, a photoreactive compound that generates ROS under light. This causes lipid peroxidation, membrane damage, and rapid cell death (Duke et al., 1991).

Acifluorfen acted rapidly, and this fast action was clearly reflected in the transcriptome response. Numerous genes involved in cellular response to hypoxia and oxidative stress were strongly induced at early exposure, indicating that abundant ROS were produced resulting from acifluorfen’s treatment. In addition, diverse signaling pathways, such as response to wounding, jasmonic acid (JA), bacterium as well as detoxification processes were activated at this stage, suggesting ROS-mediated activation of plant defense mechanisms (Glawischnig, 2007; Fatma et al., 2022; Saberi Riseh et al., 2024).

Atrazine is a photosystem II (PSII) inhibitor that binds to D1 protein at the Quinone B (*Q_B_*)-binding site in the thylakoid membranes. This interaction blocks electron transport from *Q_A_* to *Q_B_*, disrupting photosynthetic electron flow and leading to excessive ROS production and plant death (Zhu et al., 2009). Interestingly, at this early treatment stage, the most enriched biological pathways were related to ribosome biogenesis, including rRNA processing and ribosomal subunit assembly, which was consistent with previous RNA-seq studies (Qu et al., 2021; da Roza et al., 2025; Li et al., 2025b). The differential regulation of ribosomal pathways associated genes likely suggested a cellular stress response aimed at counteracting the herbicide’s effects. In addition, pathways associated with metabolic processes such as *L*-amino acids and alpha-amino acids were also affected (**Supplementary Table S7**), indicating adjustments in plant metabolism during the early phase of exposure (Zhang et al., 2021; Sun et al., 2022; Li et al., 2025b).

Imazethapyr is an acetolactate synthase (ALS) inhibitor that targets a key enzyme in the biosynthesis of branched-chain amino acids (valine, leucine, and isoleucine) in plants. It binds to ALS enzyme, preventing substrate conversion and leading to depletion of essential amino acids, which inhibits protein synthesis and plant growth. Under this treatment, imazethapyr did not trigger large transcriptomic change, but primarily affected genes associated with photosynthetic activities, including those involved in electron transport in PSII and the cyclic electron transport pathway. This is consistent with previous findings that ALS inhibitors negatively impact photosynthesis efficiency in plants (Qian et al., 2011; Liu et al., 2019). Specifically, earlier studies reported that ALS inhibition induced activity of the cyclic electron transport pathway by upregulating the NDH complex and downregulating the PGR5 pathway (Qian et al., 2013; Sun et al., 2016). In addition, defense pathways such as the glucosinolate biosynthetic pathway and detoxification-related genes, such as ABC transporters and UGTs, were up-regulated at this stage, reflecting an early xenobiotic stress response.

Mesotrione is a 4-hydroxyphenylpyruvate dioxygenase (HPPD) inhibitor that interferes with carotenoid biosynthesis. It binds to the HPPD enzyme, blocking the production of plastoquinone (PQ). PQ is an essential cofactor for phytoene desaturase, a key enzyme in carotenoid synthesis. As a result, chlorophyll becomes unprotected from photooxidation. This leads to excessive ROS accumulation and widespread oxidative stress, resulting in bleaching symptoms and plant death (Mitchell et al., 2001; Sun et al., 2023). Consistent with this mechanism, transcriptomic responses to mesotrione treatment showed strong induction of genes associated with hypoxia response and oxidative stress. Besides, genes involved in trehalose biosynthesis were also induced (**Supplementary Table S7**), reflecting the role of trehalose in mitigating osmotic and oxidative stress by stabilizing cellular structure and supporting stress tolerance (Avonce et al., 2006; Eh et al., 2025).

Paraquat, another fast-acting herbicide, is a photosystem I (PSI) electron diverter that accepts electrons from PSI and transfers them to molecular oxygen, resulting in the rapid generation of ROS, including superoxide and hydroxyl radicals. This redox cycling causes oxidative damage to cellular membranes and leads to rapid desiccation and cell death (Nazish et al., 2022). GO enrichment analysis at the early treatment stage revealed prominent induction of genes associated with hypoxia response, reflecting the oxygen depletion caused by excessive ROS production. In addition, defense-related pathways were strongly enriched, particularly genes involved in phenylpropanoid/flavonol signaling pathways (**Supplementary Table S7**) (Lee et al., 2003). These results suggested that herbicides engaged distinct regulatory pathways that were reflective of their primary biochemical targets at low doses and early exposure.

#### 3.3.2 Prolonged low-dose treatment shifted responses from primary-target-associated pathways to defense signaling pathways

At IC_50__24 h (**Figure 2**), the top enriched pathways across herbicides remained largely associated with primary target processes, while starting to shift toward defense signaling pathways. Under prolonged 2,4-D treatment, plant responses expanded beyond the predominantly auxin-related pathways observed earlier. Although auxin response pathways were still detected, the transcriptional profile became increasingly highlighted with defense signaling pathways such as salicylic acid (SA) response, and broader regulation of stress responses. Acifluorfen remained centered on oxidative stress responses, but additional defense signaling pathways (fungus and wounding responses) and immune response were also enriched. In contrast, atrazine exhibited relatively limited transcriptional changes compared to earlier treatment, with enrichment still largely confined to ribosome biogenesis and *L*- and alpha-aminooxy acid biosynthetic/metabolic processes. Imazethapyr induced a broader transcriptomic response at this stage, with strong induction of genes associated with ribosomal biogenesis, such as ribosomal RNA (rRNA) transcription, rRNA processing, and ribosome assembly. This was likely due to the disruption of branched-chain amino acids biosynthesis triggered by ALS inhibition, which in turn activated ribosomal stress pathways. As a compensatory mechanism, plants upregulated genes involved in ribosome assembly and rRNA processing to restore translational capacity and synthesize stress-responsive proteins (Ohbayashi and Sugiyama, 2017). Besides, processes related to photosynthesis and protein localization to mitochondria were triggered. Mesotrione remained enriched for oxidative stress-related processes, while additional stress responses, including SA signaling, water deprivation, and detoxification pathways, were subsequently activated. Paraquat, like mesotrione, triggered the induction of numerous genes associated with hypoxia response and defense signaling involved in responses to fungus, immune system process, toxic substances and heat. Collectively, these results indicated that at this stage, plant responses started to transition from processes directly tied to the herbicide’s primary target to diverse defense, immunity and detoxification mechanisms.

Glyphosate is a non-selective herbicide that inhibits the enzyme 5-enolpyruvylshikimate-3-phosphate synthase (EPSPS). This enzyme plays a crucial role in the shikimate pathway, which is essential to produce aromatic amino acids (phenylalanine, tyrosine, and tryptophan) in plants. Glyphosate binds to the active site of EPSPS, blocking the synthesis of aromatic amino acids and leading to metabolic imbalance, growth inhibition, and ultimately plant death (Duke, 2021). Under our RNA-seq analysis parameters, limited DEGs reached the stringency threshold for IC_50_ treatments. No genes were regulated at IC_50__4 h, while only two were downregulated at IC_50__24 h, identified as AT1G55673 that encodes a hypothetical protein and AT5G16080 that encodes carboxyesterase 17. This observation was consistent with previous reports suggesting that glyphosate triggers relatively weak or slower transcriptional responses at sublethal concentrations, leading to a limited number of genes being differentially regulated compared to other herbicides tested here (Das et al., 2010; da Roza et al., 2025). In addition, glyphosate is a slow-acting herbicide. Its complete transcriptional responses may not be fully captured at the 4 h and 24 h sampling intervals. Consequently, comparisons of transcriptional responses to glyphosate and other herbicides require cautious interpretation.

#### 3.3.3 Early high-dose treatment revealed both divergent and convergent transcriptional responses across herbicides

Under IC_99__4 h treatment conditions, the transcriptomic responses to several herbicides resembled those observed at IC_50__24 h, while others diverged substantially (**Figure 3**). For 2,4-D, acifluorfen, mesotrione, and paraquat, although IC_50__24 h and IC_99__4 h represented distinct exposure regimes, the top enriched biological pathways in both treatments were predominantly associated with stress responses and defense-related pathways. This indicated that acute lethal stress and prolonged sublethal exposure could converge on similar downstream transcriptional programs in *Arabidopsis* when comparable physiological thresholds were reached (Rasmussen et al., 2013; Rurek and Smolibowski, 2024). In contrast, atrazine and imazethapyr displayed a different shift in their transcriptomic reprogramming at IC_99__4 h. In addition to the RNA biogenesis or *L*- and alpha- amino acid biosynthetic/metabolic processes observed at IC_50__24 h, both herbicides at IC_99__4 h majorly induced diverse stress responsive pathways, suggesting that *Arabidopsis* prioritized metabolic and biosynthetic reprogramming under long-term sublethal stress but rapidly redirected transcription toward stress response under short-term lethal conditions (Witwicka et al., 2025).

Among these, glyphosate is particularly distinct. Despite eliciting little transcriptomic changes at both IC_50_ time points, at IC_99__4 h, it triggered a robust induction of stress responsive pathways, including responses to water deprivation, hyperosmotic salinity, hydrogen peroxide, hypoxia, wounding and JA. In addition, this treatment condition enriched the mitochondria-nucleus signaling pathway (**Supplementary Table S7**), possibly reflecting a retrograde signaling resulting from mitochondria dysfunction. This is consistent with previous studies which report that mitochondria function was disrupted under glyphosate exposure due to oxidative stress through H_2_O_2_ accumulation (Gomes and Juneau, 2016; Strilbyska et al., 2022).

#### 3.3.4 Prolonged high-dose treatment revealed convergence on common stress response-related pathways

The top biological pathways for most herbicide treatments overlapped (**Figure 4**) at IC_99__24 h and were mainly enriched in processes associated with (cellular) responses to oxidative stress, reflecting increasing physiological stress. Oxidative stress-related GO terms appeared across most herbicide treatments, and the top enriched pathways consistently centered on cellular responses to oxidative stress. This pattern aligned with previous findings showing that diverse herbicides, regardless of mode of action, often converged on secondary oxidative stress through excessive ROS accumulation once the primary target was blocked (Caverzan et al., 2019; Kaur, 2019; Traxler et al., 2023). At this lethal exposure level, the strong induction of oxidative stress pathways suggested that cells may initiate programmed cell death (PCD) processes as part of the terminal stress response (Van Breusegem and Dat, 2006; Petrov et al., 2015). Besides, individual herbicides also activated additional defense-related pathways, including SA and JA signaling, glucosinolate biosynthesis, and detoxification-related processes.

### 3.4 Herbicide-specific gene expression patterns revealed both MOA-associated and broader responses

While functional analysis of the complete set of DEGs revealed both divergent and convergent biological pathways under the four treatment conditions, it may obscure herbicide specific transcriptional signatures as numerous regulated genes are involved in general stress response pathways triggered by herbicide exposure. To address this, we next focused on distinct gene sets representing herbicide-specific responses under each treatment condition, defined here as “unique DEGs” regulated by a single herbicide and absent from the responses to other herbicides under the same condition. UpSet plots were generated using the complete DEG sets from each herbicide treatment to visualize both the number of unique genes and the intersections shared among herbicides (**Figure 7**). The unique DEGs list, number of unique DEGs and their percentage relative to the complete DEGs for each treatment are summarized in **Supplementary Table S6** and **Supplementary Table S13B**, respectively. Overall, the percentage was higher under short term (4 h) exposure than under long-term (24 h) exposure for both IC_50_ and IC_99_ treatments. When comparing concentration levels, herbicides generally induced a greater proportion of unique genes at IC_50_ than at IC_99_. This pattern was consistent with the GO enrichment analysis of the complete DEGs discussed above. The top GO terms identified for each herbicide at IC_50__4 h were largely distinct from each other, indicating inductions of a large number of MOA-associated genes (**Figure 1**). In contrast, under higher concentrations and longer exposure times, the dominant GO terms became more convergent across herbicides, suggesting fewer responses of MOA associated genes and more induction of similar biological processes such as defense-related pathways under severe stress conditions (**Figure 4**).

**Figure 7:**
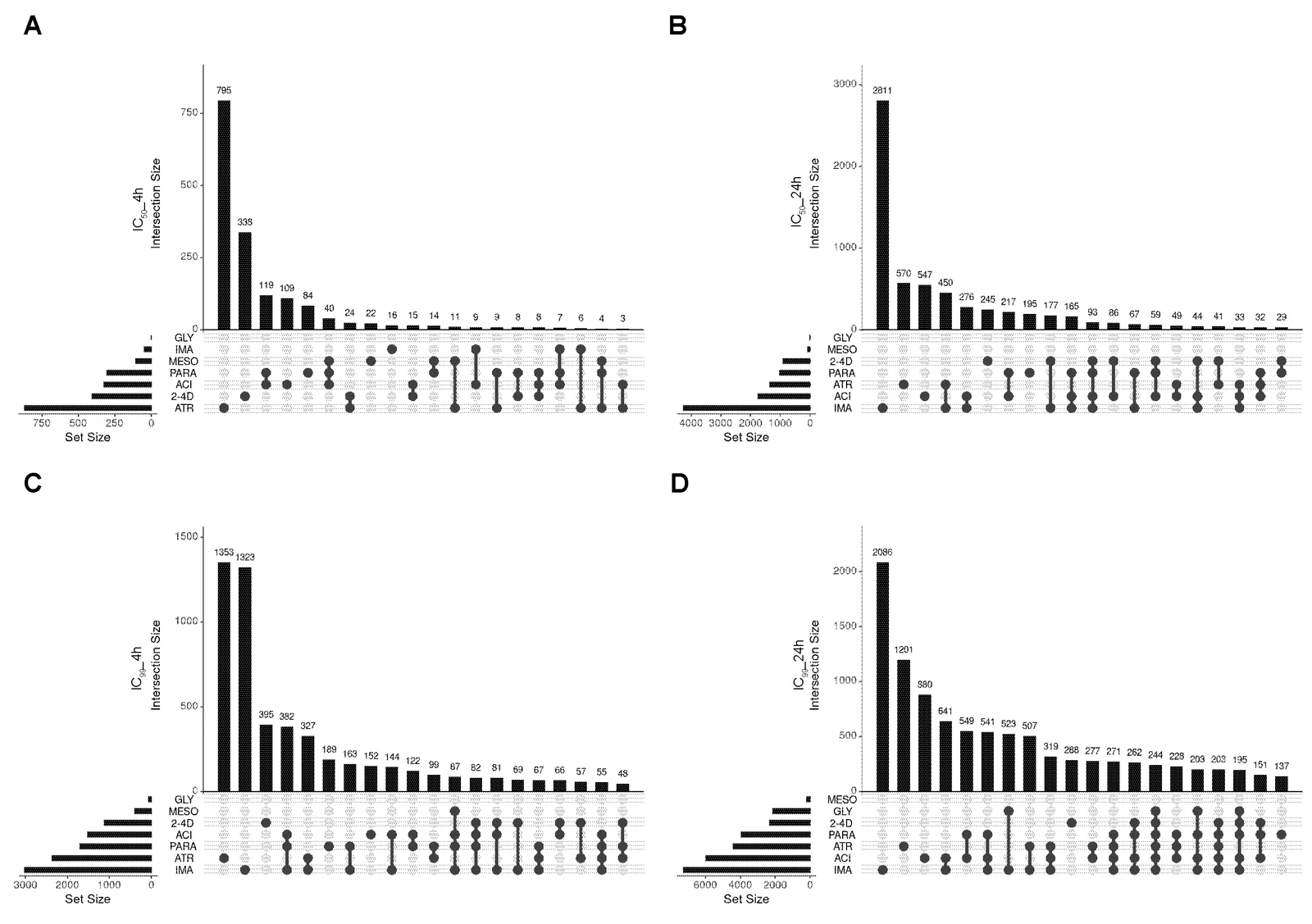
Unique and shared differentially expressed genes across herbicide treatments. Visualization of unique and shared differentially expressed genes among seven herbicides under four treatment conditions: **(A)** IC_50__4 h; **(B)** IC_50__24 h; **(C)** IC_99__4 h; **(D)** IC_99__24 **(h)** UpSet plots were generated using the Intervene Shiny App, with a maximum display limit of 20 intersections per plot. Thus, unique gene sets for some herbicides are not shown here because they fall outside the top 20 intersections. Full unique DEGs numbers for each herbicide are listed in **Supplementary Table S13A**. Unique gene lists were extracted by running Venn Diagram on https://bioinformatics.psb.ugent.be/webtools/Venn/ and are provided in **Supplementary Table S6**.

#### 3.4.1 Clustering resolved herbicide-specific gene expression patterns and associated functional pathways

To investigate herbicide-specific gene expression patterns, we combined all unique DEGs identified under the four treatment conditions and removed duplicate genes to generate a single non-redundant set of unique DEGs for each herbicide. We then performed hierarchical clustering based on the expression profiles (raw log_2_ fold change) of these gene sets and analyzed GO enrichment on the resulting data. The clustering approach enabled the identification of coexpressed gene modules with similar expression profiles across the four treatment conditions (Eisen et al., 1998). Based on the expression patterns, gene clusters were descriptively grouped as early responsive, late responsive, time-related, progressive responsive and consistent responsive to facilitate the interpretation of observed transcriptional trends. In this study, the gene clusters represent observed expression patterns and were not derived from formal statistical tests of dose, time or dose x time interactions analyses. The early-responsive gene cluster was defined as genes showing strong induction/repression at 4 h but weaker change at 24 h. Late-responsive genes had minimal changes at 4 h but strong regulation at 24 h. Time-related genes were defined as genes whose expression profiles differed between the 4 h and 24 h treatment conditions while showing relatively similar responses at IC_50_ and IC_99_. Progressive-responsive genes were defined as genes whose expression changes generally increased across the four treatment conditions, with the strongest response observed at IC_99__24 h, while consistent-responsive gene sets displayed similar regulation across all four treatment conditions. The purpose of grouping these gene clusters was to illustrate representative expression patterns across the four treatment conditions. Consequently, it should be interpreted as descriptive classifications rather than statistical analyses.

Based on the above defined transcriptional modules and their associated functional enrichment analysis, we next summarized the major expression patterns observed for each herbicide, highlighting representative clusters and their biological interpretations. The hierarchical clustering heatmap for each herbicide and one representative functional GO term from each cluster are presented in **Figure 8**, key characteristics of individual cluster and representative genes are summarized in **Supplementary Table S15**, and the detailed cluster membership information (such as gene list, RNA-seq expression data and brief gene descriptions) along with their simplified GO dot plots are compiled together in **Supplementary Table S16** and **Supplementary Figures S3–S9**, respectively.

**Figure 8:**
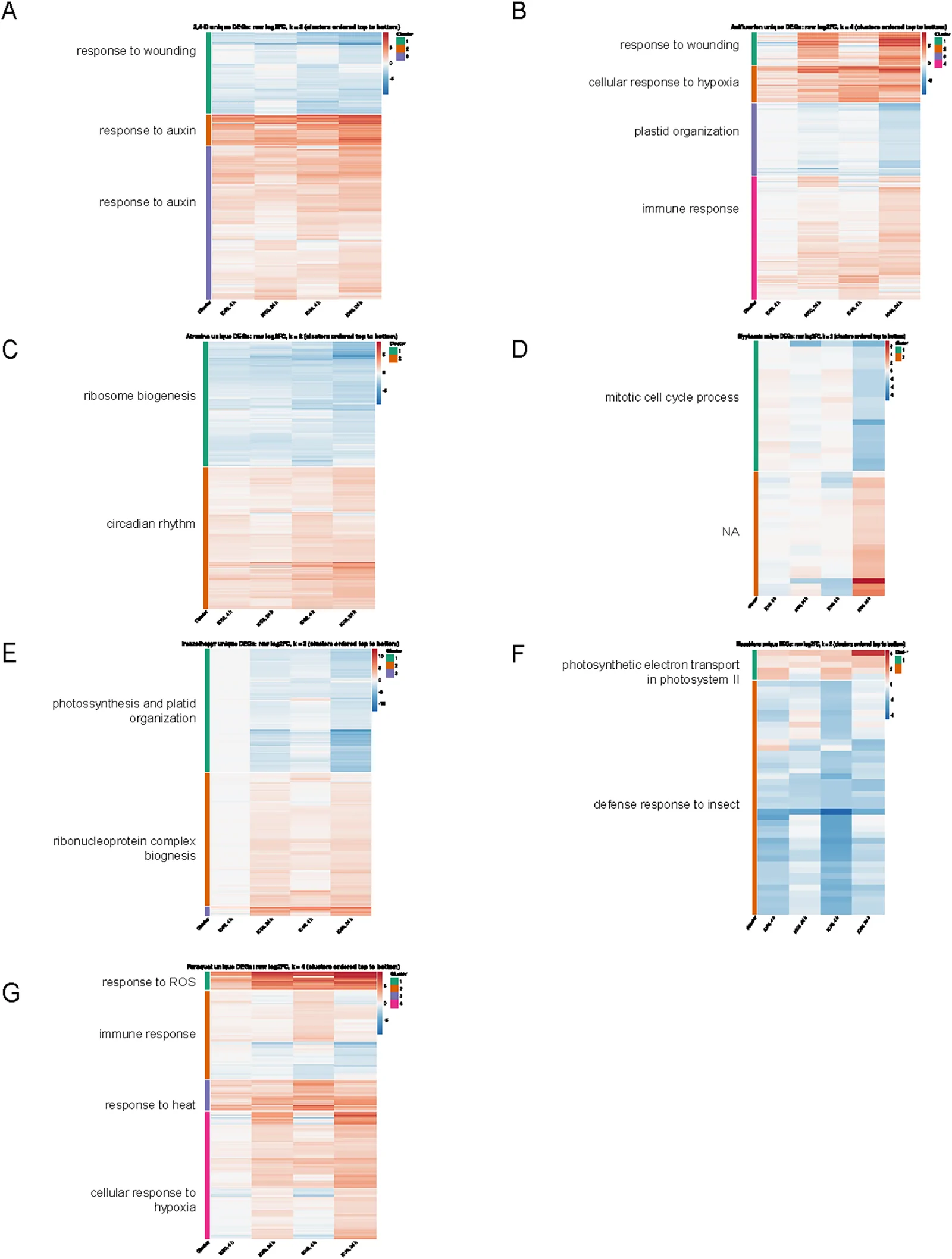
Hierarchical clustering analysis on unique differentially expressed genes across four treatment conditions. Heatmaps show raw log_2_ fold change values for unique DEGs under treatment with **(A)** 2,4-D; **(B)** acifluorfen; **(C)** atrazine; **(D)** glyphosate; **(E)** imazethapyr; **(F)** mesotrione; and **(G)** paraquat. Genes were clustered using Euclidean distance and Ward.D2 linkage. Treatment conditions were maintained in the fixed order: IC_50__4 h; IC_50__24 h; IC_99__4 h; IC_99__24 (h) Based on elbow and silhouette analyses, genes were divided into 3, 4, 2, 2, 3, 2, and 4 clusters for A-G, respectively. Cluster numbers correspond to the top-to-bottom positions in each heatmap. Representative GO functional theme for each cluster is the top term ranked by adjusted *p* value from simplified cluster-specific GO-BP enrichment analyses. The complete unsimplified cluster-specific GO enrichment analysis results are provided in **Supplementary Table S10**.

For 2,4-D, hierarchical clustering partitioned the unique DEGs into three clusters (**Figure 8A**). Cluster 1 comprised genes that were consistently repressed across treatments, whereas clusters 2 and 3 exhibited consistently induced patterns. Notably, cluster 2 showed a strong, progressive increase in expression from IC_50_ to IC_99_ treatments, while cluster 3 displayed a similar but lower induction magnitude. Functional enrichment analysis revealed that clusters 1 was predominantly enriched for stress-related biological processes, including responses to wounding, responses to jasmonic acid stimulus and glucosinolate biosynthesis, whereas cluster 2 and 3 were primarily enriched for auxin-related processes. The two auxin enriched clusters included representative auxin-responsive genes such as SAUR and GH3 family members (**Supplementary Table S15**; **Supplementary Figure S3**, and **Supplementary Table S16**).

There were four distinct clusters for acifluorfen unique DEGs (**Figure 8B**). Cluster 1 exhibited a time-related induction pattern, with strong upregulation at 24 h, while cluster 2 showed a progressive induction pattern associated with both treatment intensity and exposure time. Both clusters were enriched for stress-related biological processes. Additionally, cluster 2 enriched in the regulation of stress responses and included many stress-responsive transcription factors. Cluster 3 represented a late repression pattern and was enriched for plastid organization and protein quality control for misfolded or incomplete synthesized proteins, suggesting suppression of photosynthetic and protein synthesis activities under prolonged severe treatment. Cluster 4 exhibited a weaker time-related induction pattern at 24 h and was enriched for immune response, regulation of defense responses, and programmed cell death. This cluster also contained genes involved in vesicle-mediated transport and detoxification, including genes from the CYP450, UGT, and ABC transporter families (**Supplementary Table S15**; **Supplementary Figure S4**, and **Supplementary Table S16**).

Unique DEGs for atrazine were clustered into two contrasting transcriptional groups (**Figure 8C**). Cluster 1 contained 1296 repressed genes, with repression generally becoming stronger as treatment intensity and duration increased and reached its maximum at IC_99__24 h. This cluster was strongly enriched for ribosome biogenesis, cytoplasmic translation, protein folding and maturation, amino acid biosynthetic process and DNA replication (**Supplementary Figure S5**). Many genes in this cluster were annotated as encoding ribosomal proteins, supporting a broad repression of ribosome-related functions and suggesting that atrazine may induce a global slowdown in protein synthesis. The enrichment of DNA replication among these repressed genes likely reflected a coordinated suppression of cell proliferation-associated activities under severe chemical stress. In contrast, cluster 2 contained 1472 induced genes, with the strongest induction generally occurring at IC_99__24 h. Enriched biological processes included circadian rhythm, response to red or far-red light, *L*-amino acid and small molecule catabolism, programmed cell death, regulation of post-embryonic development. The enrichment of *L*-amino acid and other catabolic processes may reflect enhanced metabolic flexibility and stress adaptation through nutrient recycling and energy management when primary metabolic processes are disrupted by herbicide exposure (Hildebrandt et al., 2015). Together, these enriched processes indicated that activation of light- and circadian-associated regulatory networks, catabolic metabolism, and broader stress response programs under atrazine treatment (**Supplementary Table S15**; **Supplementary Figure S5**; **Supplementary Table S16**).

Within glyphosate, hierarchical clustering produced a simple two-cluster structure with a small size, indicating the limited number of unique DEGs detected at the fixed sample times (4 h and 24 h) in this study. Because glyphosate is a slow-acting herbicide, these sampling intervals may not fully capture the complete temporal progression of its transcriptional responses (**Figure 8D**). Transcriptional responses were minimal at both time points under IC_50_, with most differential expression starting at IC_99__24 h, representing a late-responsive pattern. Cluster 1 included late repressed genes and was enriched in processes related to nuclear division, organelle organization, mitotic cell cycle regulation, and chromosome organization, likely suggesting that severe EPSPS inhibition ultimately led to cell-cycle arrest and suppression of growth-associated functions, consistent with metabolic starvation caused by disrupted aromatic amino acid biosynthesis. In contrast, cluster 2 exhibited relatively strong induction primarily under the IC_99__24 h. However, no significantly enriched GO terms were identified for this cluster (**Supplementary Table S15**, **Supplementary Figure S6**, and **Supplementary Table S16**).

Hierarchical clustering of DEGs in response to imazethapyr identified three major expression patterns (**Figure 8E**). Cluster 1 and 2 consisted of time-related regulated genes, which both had stronger regulations at 24 h under both dosage treatments. GO enrichment revealed that cluster 1 was dominated by processes associated with photosynthesis, microtubule-based movement, and actin cytoskeleton organization (**Supplementary Figure S7**). Stronger downregulation of these pathways under prolonged imazethapyr exposure was consistent with previous reports that ALS inhibitors restricted branched-chain amino acids (leucine, isoleucine, and valine) availability, thus limiting protein synthesis, suppressing cell division and growth, and reducing photosynthetic capacity through chlorophyll loss and chloroplast damage. These changes matched the growth-arresting effects typically observed when branched-chain amino acid biosynthesis was blocked in susceptible plants (Zabalza et al., 2013; Dor et al., 2017; Liu et al., 2019). Cluster 2 contained upregulated genes that triggered stronger induction under IC_50__24 h and IC_99__24 h. GO analysis of this gene set showed strong enrichment in ribosomal biogenesis. About 130 genes in this cluster encoded ribosomal proteins and rRNA processing proteins (**Supplementary Table S16**). Upregulation of these processes could reflect cellular attempts to rebalance amino acid homeostasis or maintain translational capacity under sustained ALS inhibition (Casey et al., 2023). This interpretation is consistent with the well-established coupling between amino acid/nutrient status and ribosome biogenesis, whereby cells dynamically adjust ribosome production and composition to match metabolic conditions and preserve protein homeostasis during stress (Hang et al., 2018; Dias-Fields and Adamala, 2022; Ni and Buszczak, 2023). Cluster 3 consisted of a relatively small gene set moderately induced at IC_50__4 h but consistently stronger induced in the subsequent three treatments. This cluster was predominantly enriched in processes associated with mitochondrial protein import and ribosome assembly, indicating a coordinated adjustment of organellar function and cellular translational capacity under imazethapyr treatment (**Supplementary Table S15**; **Supplementary Figure S7**; and **Supplementary Table S16**).

For mesotrione, hierarchical clustering simply generated two clusters (**Figure 8F**). Cluster 1 comprised a small set of genes that were consistently and moderately upregulated across treatments and enriched in photosynthetic energy metabolism, redox homeostasis, environmental acclimation, and protein glycosylation. Cluster 2 exhibited an early responsive pattern with relatively strong repression at 4 h followed by weaker repression at 24 h under both dosages. This cluster was enriched in biological processes related to responses to external stimuli and general biological regulation (**Supplementary Figure S8**), including several stress-associated transcription factors (**Supplementary Table S15**). This temporal pattern may suggest an early transcriptional suppression of stimulus-responsive pathways under acute herbicide exposure followed by a partial acclimation or transcriptional stabilization under sustained exposure.

Hierarchical clustering of paraquat unique DEGs revealed four distinct transcriptional response patterns (**Figure 8G**). Clusters 1 and 4 exhibited similar time-related induction at 24 h under both dosage treatments, with cluster 1 displaying a higher induction magnitude than cluster 4. Functional enrichment revealed that cluster 1 was associated with oxidative stress responses and protein-containing complex organization and assembly, suggesting that prolonged exposure to paraquat progressively activated oxidative stress defense mechanisms and protein complex remodeling, consistent with sustained cellular damage caused by membrane destabilization (Nazish et al., 2022). In contrast, cluster 2 represented a group of early-responsive genes that exhibited complex transcriptional expression patterns. These genes overall showed weak induction at IC_50__4 h, attenuated regulation at IC_50__24 h, followed by stronger induction at IC_99__4 h, and subsequent attenuation again at IC_99__24 h. Notably, multiple photosystem components, such as chloroplast ribosomal protein, PSI proteins, PSII reaction center protein B and photosynthetic electron transfer B, were also present in this cluster (**Supplementary Table S16**), indicating a transcriptional response closely associated with paraquat’s mode of action. Cluster 3 represented a smaller gene set that showed consistent induction across four treatments and enriched in processes associated with responses to different stresses and protein folding and autoubiquitination (**Supplementary Table S15**, **Supplementary Figure S9**, and **Supplementary Table S16**).

#### 3.4.2 Comparative analysis of herbicide-specific expression patterns resolved both MOA associated responses and convergent biological outcomes

Across herbicides, enriched functional pathways exhibited diverse temporal transcriptional patterns (**Supplementary Table S15**). To evaluate whether distinct transcriptional dynamics reflected herbicide MOA-associated responses, we compared the biological functions and expression patterns of transcriptional modules described above across herbicides (**Table 2**).

**Table 2:** Comparison of dominant functional biological effects and their expression patterns across herbicides.

Functional biological effects		2,4-D	ACI	ATR	GLY	IMA	MESO	PARA
Auxin response		C2&C3 consistent induction						
Photosynthesis/chloroplast repression			C3 late repression			C1 time-related repression		
Ribosome biogenesis				C1 consistent repression		C2 time-related induction		
*L*-and α-amino acid catabolic processes				C2 consistent induction				
Stress response-related processes	Hormone signaling	C2&C3 consistent induction						
	Oxidative stress		C2 dose- and time-related progressive induction					C1 time-related induction
	Response to different stimulus	C1 consistent repression	C1 time-related induction	C2 consistent induction			C2 early response, time-related repression	C4 time-related induction
	Immune/defense response		C4 time-related induction					C2 early responsive
	Adaptive stress response such as protein autoubiquitination							C3 consistent induction
Cell cycle/growth inhibition-related processes	Suppression of DNA replication/repair			C1 consistent repression (strongest repression at IC_99_ _24h)				
	Suppression of nuclear division/mitotic cell cycle regulation				C1 late repression			
	Suppression of cytoskeleton organization/microtubule-based process					C1 time-related repression (strong repression at 24h)		

C, cluster; 2,4-D, 2,4-dichlorophenoxyacetic acid; ACI, acifluorfen; ATR, atrazine; GLY, glyphosate; IMA, imazethapyr; MESO, mesotrione; PARA, paraquat.

##### 3.4.2.1 Distinct transcriptional dynamics reflected herbicide MOA-associated responses

For 2,4-D, responses linked to its known MOA-associated pathways, auxin responses, were primarily enriched in Clusters 2 and 3, which were characterized by consistent induction across doses and time points (**Supplementary Figure S3**). In contrast, for acifluorfen, a PPO inhibitor, photosynthesis-related pathways are not the primary molecular targets but represent MOA-associated secondary damage resulting from excessive ROS generated following PPO inhibition. Accordingly, genes associated with photosynthesis were predominantly assigned to late-responsive clusters and exhibited pronounced repression at 24 h (**Figure 8B**), a transcriptional pattern that is biologically consistent with ROS-mediated impairment of photosynthetic machinery following PPO inhibition.

Similar to acifluorfen, imazethapyr, an ALS inhibitor, also exhibited a cluster of genes involved in photosynthesis-related pathways that were downregulated in a late, time-related manner (**Supplementary Figure S7**), with pronounced repression observed at 24 h under both doses. As photosynthesis is not the primary molecular target of ALS inhibitors, this response also likely represents a secondary MOA-associated effect resulting from impaired chloroplast maintenance caused by reduced branched-chain amino acid synthesis. In addition to photosynthesis-related repression, imazethapyr treatment also exhibited a distinct cluster enriched for ribosome biogenesis and translation-related processes. Genes in this pathway displayed a time-related induction pattern, with strong upregulation observed at 24 h under both doses (**Supplementary Figure S7**). This response likely represented another secondary MOA-associated effect driven by branched-chain amino acid starvation. The induction of ribosome-related genes may reflect an adaptive transcriptional response aimed at maintaining translational capacity under limited conditions of amino acid synthesis.

In contrast, atrazine, a PSII inhibitor, also affected ribosome biogenesis-related genes, but these genes were consistently repressed across the four treatments (**Supplementary Figure S5**). This opposite transcriptional pattern possibly reflected fundamental differences in the physiological stress imposed by PSII inhibition, where rapid disruption of photosynthetic electron transport led to severe energy limitation and oxidative stress. Under such conditions, energetically costly processes such as ribosome biogenesis were suppressed, resulting in transcriptional downregulation of translation-related pathways. In addition, plants under atrazine treatment also exhibited a distinct cluster characterized by consistent induction of *L*-and alpha-amino acid catabolic processes (**Supplementary Figure S5**), representing another secondary MOA-associated pathway and possibly reflecting a metabolic adaptation to energy limitation caused by impaired photosynthetic electron transport. Under PSII inhibition, reduced ATP and NADPH production necessitated the mobilization of alternative energy sources, and amino acid catabolism substantially contributed carbon skeletons and respiratory substrates to support cellular energy demands when carbohydrate supply was restricted. Induction of amino acid degradation under energy-deficient conditions has been previously observed in plants and is considered part of a broader metabolic response (Hildebrandt et al., 2015; Heinemann and Hildebrandt, 2021).

Notably, mesotrione and paraquat were the only two herbicides that exhibited early responsive transcriptional clusters (mesotrione cluster 2, paraquat cluster 2) in this study (**Figures 8F, G**). For paraquat, this cluster (**Figure 8G**) also contained a small subset of genes encoding photosystem components, including PSI PsaA/B, PSII reaction center proteins, and photosynthetic electron transfer proteins (**Supplementary Table S15**, **Supplementary Table S16**). Interestingly, although these genes were clustered together with generally upregulated stress-responsive regulators based on their similar temporal expression dynamics (Hestilow and Huang, 2009), they displayed overall repression rather than induction (**Figure 8G**). This pattern is consistent with rapid, transient transcriptional suppression of photosynthetic machinery after acute PSI disruption following paraquat exposure, reflecting early functional damage of MOA-associated pathways (Imadi et al., 2015).

##### 3.4.2.2 Diverse functional pathways converged into common physiological responses

Beyond the MOA-associated functional pathways described above, there were other enriched functional pathways identified across herbicides. These included hormone signaling notably for 2,4-D treatment, oxidative stress responses and immune/defense response under acifluorfen and paraquat treatments, adaptive stress response such as protein autoubiquitination under paraquat treatment. Additionally, general responses, including responses to different stimuli, were observed across most herbicide treatments (**Table 2**). Despite differences in gene identity and expression dynamics, the annotated GO terms consistently converged on a unified biological outcome, stress response related processes. This indicates that plants cope with herbicide exposure at comparable levels of growth inhibition through distinct transcriptional regulatory programs, which ultimately converge on common stress response-related processes, reflecting a common physiological response to chemical perturbation.

Several herbicide treatments exhibited enrichment of pathways associated with cell cycle- and growth-related processes, including suppression of DNA replication/repair, suppression of nuclear division or mitotic cell cycle regulation, and suppression of cytoskeleton organization (**Table 2**). Notably, all these pathways displayed late or progressive expression dynamics, consistent with cumulative physiological constraints under prolonged or lethal herbicide exposure.

In addition, genes involved in xenobiotic metabolism and transport, including CYP450s, ABC transporters, UGTs and GSTs, were recurrently represented across herbicide-responsive clusters, indicating that detoxification constitutes a shared response to the different herbicide treatments (**Supplementary Table S16**).

Collectively, these comparative analyses indicated that although each herbicide elicited a largely distinct set of responsive genes, plants ultimately engaged in conserved stress response related, detoxification-related, and growth-regulatory-related processes with distinct temporal dynamics during herbicide exposure, consistent with previous studies (Wang et al., 2021; Markus et al., 2021; Chen et al., 2023; Yang et al., 2025).

### 3.5 RT-qPCR validation confirmed RNA-seq expression patterns

To validate the RNA-seq results, a subset of representative genes (**Supplementary Table S12**) was selected for RT-qPCR analysis, including MOA-associated genes, cluster-representative genes and genes involved in common functional detoxification pathways such as CYP450, UGT and ABC transporters. Detailed information for these representative genes, including gene names and corresponding RNA-seq data, is provided in **Supplementary Table S11**. Gene expression levels under IC_50_ and IC_99_ treatments were compared against the respective controls within each time point (4 h or 24 h). Due to limited RNA samples availability, the 4 h RT-qPCR analyses for paraquat and mesotrione were performed using a single biological sample with three technical replicates (**Supplementary Figures S17, S18**), all other RT-qPCR measurements represented three biological replicates. Therefore, the corresponding 4 h RT-qPCR results for paraquat and mesotrione should be interpreted as qualitative validation of the RNA-seq expression pattern but do not assess biological variations. Overall, RT-qPCR results showed expression patterns generally consistent with those obtained from RNA-seq, with most genes displaying similar directions of regulation across treatments (**Figure 9**, **Supplementary Figures S12–S18**). Technical replicates showed high consistency, while variation was observed among biological replicates, which was expected due to inherent biological variability in plant response to herbicide treatment. Despite this variation, the general expression trends were consistent across biological replicates and aligned well with the expression patterns identified from RNA-seq data (**Figure 9**, **Supplementary Figures S12–S18**, **Supplementary Table S11**), confirming the reliability of the transcriptomic analysis.

**Figure 9:**
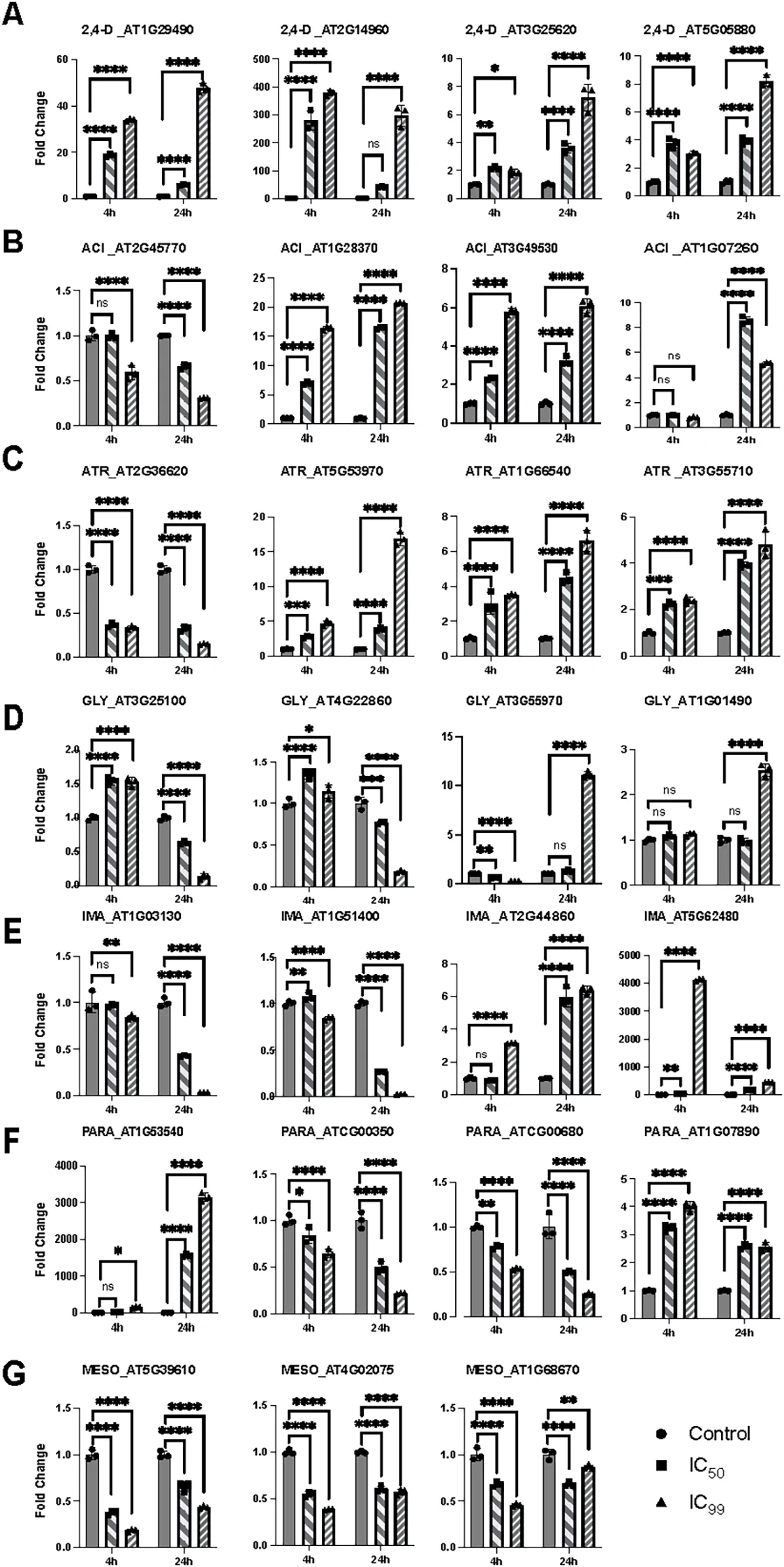
Validation of RNA-seq results by RT-qPCR. Relative expression levels of selected representative genes for each herbicide **(A–G)** were measured by RT-qPCR under four treatment conditions. Tested gene IDs are labeled in the graphs. Three independent biological replicates were analyzed, with three technical replicates performed per biological replicate. RT-qPCR data from one representative biological replicate is shown, as similar expression trends were observed across all biological replicates. Full RT-qPCR datasets, including all three biological replicate for each gene, are provided in **Supplementary Figures S12–S18**. Gene expression levels were normalized to reference gene *PP2A* and calculated relative to the control at each time point using the 2^-ΔΔCt^ method. The expression patterns observed by RT-qPCR are consistent with the corresponding RNA-seq results.

## 4 Discussion

### 4.1 A physiologically defined framework enables comparative transcriptional analysis across herbicides

In this study, we integrated physiological and transcriptomic analysis to systematically characterize plant responses to seven herbicides with distinct modes of action. Dose-response analyses were first performed to determine herbicide IC_50_ and IC_99_ values based on shoot fresh weight reduction four days after treatment, establishing a framework of physiologically defined treatment levels that standardized herbicide treatment intensity. This framework enabled comparative transcriptomic analyses under treatment conditions representing moderate (IC_50_) and severe (IC_99_) growth inhibition. However, although IC_50_ and IC_99_ treatment standardized herbicide treatment according to their ultimate growth inhibitory effects, they did not necessarily correspond to the same physiological states at the 4 h and 24 h RNA-seq sampling time points because herbicides with different modes of action differ in uptake, translocation, and rates of action. Consequently, the transcriptomic responses presented in this study should be interpreted as comparisons of herbicide responses under standardized treatment intensities and time points rather than the same physiological states at each sampling time.

Using this framework, transcriptomic profiling across multiple herbicides, doses, and time points revealed unique DEGs for each herbicide. These unique gene sets were further grouped through temporal clustering, and their distinct transcriptional expression types, including early/late, time-related, consistent and progressive response patterns, were revealed. Dominant GO terms were subsequently assigned to each cluster, and comparative analysis of functional enrichment together with cluster-specific expression dynamics uncovered herbicide-specific MOA-associated pathways across herbicides. Additionally, broader functional themes, such as stress responses-related, growth inhibition-related and detoxification-related processes, converged across herbicides despite minimal overlap at the individual gene level, indicating conserved physiological outcomes arising from distinct transcriptional regulations. Collectively, these results provide a comprehensive view of how herbicide-specific treatment levels, defined by growth inhibition, are associated with coordinated transcriptional reprogramming across distinct modes of action. The identified expression signatures provide a structured view of how different herbicides elicited unique yet dynamic molecular responses, and these transcriptional signatures have potential utility as molecular markers for identifying unknown herbicides or compounds with a similar chemical backbone or a common primary target, and they may also offer mechanistic clues for understanding herbicide detoxification responses and herbicide resistance evolution.

### 4.2 Variations in dose-response profiles underlies divergent global transcriptional reprogramming

Dose-response analysis revealed notable differences in the dynamic range and shape of herbicide responses. Some herbicides, such as acifluorfen, imazethapyr and paraquat, exhibited a rapid transition from sublethal to severe physiological effects. The full inhibition effect was completely captured within the tested concentrations and thus resulted in well-defined sigmoidal curves (**Figures 5C, F, H**). In contrast, other herbicides, including 2,4-D and mesotrione, displayed a broader dynamic range, and required a wider concentration range to capture the full range of herbicidal effects. As a result, the tested concentrations in a single experimental series did not always encompass the concentration required to induce severe physiological effect, leading to partial sigmoidal curves and less precisely constrained IC_99_ estimates (**Figures 5B, G**). Although the IC_99_ estimates for 2,4-D and mesotrione exhibited greater uncertainty, we do not consider this limitation to undermine the rationale of the comparative transcriptomic framework. The treatment concentrations were originally determined from dose-response experiments based on shoot fresh weight reduction to represent moderate (IC_50_) and severe (IC_99_) physiological inhibition (**Supplementary Figure S10**) under each herbicide treatment rather than to define exact IC_50_ and IC_99_ values. Therefore, the comparisons in this study should be interpreted as representing transcriptional responses under comparable moderate/severe physiological stress rather than exact equivalence of IC_50_/IC_99_ estimates. Nevertheless, because the IC_99_ estimates for 2,4-D and mesotrione extrapolated beyond the dose-response curves, comparisons related to precise quantitative differences at their IC_99_ treatments should be interpreted with caution while broader comparisons related to transcriptional response patterns in this study remain appropriate.

Differences in dose-response profiles were reflected in global transcriptional variations. Several herbicides triggered globally extensive transcriptional reprogramming, characterized by large numbers of DEGs, especially at severe conditions (IC_99__24 h). Previous transcriptomic studies in *Arabidopsis* have demonstrated that exposure to high light or excessive ROS due to strong singlet oxygen or PSII photoinhibition, induced hundreds of ROS-marker genes and broad defense-like transcriptional responses. These findings underscored that once a chloroplast oxidative stress threshold was exceeded, transcriptomic reprogramming became extensive (Rasmussen et al., 2013). This mechanism likely explains the pronounced transcriptional responses observed for acifluorfen, atrazine, imazethapyr and paraquat in our study (**Figure 6C**, **Supplementary Table S13A**), as these compounds imposed strong secondary effects on photosynthetic processes and redox homeostasis. Notably, the transcriptional changes detected here were consistent with previously reported RNA-seq profiles for these herbicides, supporting the biological relevance and reproducibility of our findings (An et al., 2014; Liu et al., 2024; Li et al., 2025a; da Roza et al., 2025).

In contrast, glyphosate and mesotrione elicited relatively limited global transcriptional changes under the 4 h and 24 h sampling intervals used in this study. As discussed above, the observations of limited transcriptional responses may reflect methodological limitations and biological property of the two herbicides and should be interpreted accordingly. Glyphosate induced limited DEGs at IC_50_ under both time points, only modest transcriptional changes at IC_99__4 h and broader regulation of general stress response genes observed only under the most severe condition (IC_99__24 h) (**Figure 6C**, **Supplementary Table S13A**). One possible explanation is the relatively slow-acting nature of glyphosate. Following application, glyphosate is translocated from target tissue to metabolic sinks, such as young, developing leaves and meristems via phloem and xylem before exerting its phytotoxic effects. As a result, herbicide requires time to accumulate in target tissue, leading to a delayed onset of physiological damage (Duke, 2021). This mode of action contributes to the slower development of transcriptional responses compared with herbicides mentioned above that affect photosynthesis or redox homeostasis. Additionally, the fixed sampling times (4 h and 24 h) employed in this comparative study may not fully capture the complete transcriptional responses to glyphosate. Collectively, the limited number of DEGs observed under glyphosate treatment may reflect both the biological property of its MOA and the sampling intervals employed in this study. Similarly, mesotrione triggered relatively weak transcriptional changes across all treatments (**Figures 6B, C**, **Supplementary Table S13A**), consistent with previous transcriptomic studies (Mesnage et al., 2018). One possible explanation could be that this compound was rapidly detoxified in many dicot species through CYP450-mediated metabolism (Ma et al., 2013), resulting in transcriptional responses that remain below our RNA-seq detection thresholds. Another reason could be that insufficient accumulation of chloroplast oxidative stress might limit activation of ROS-responsive transcriptional programs. Supporting this interpretation, classical ROS marker genes, including respiratory burst oxidase homologues (RBOHs) and ascorbate peroxidase (APX), were not differentially expressed across all mesotrione treatments under our RNA-seq analysis parameters (**Supplementary Table S5**), indicating that strong oxidative stress signaling possibly was not induced. Additionally, the greater uncertainty of IC_99_ estimate was a possible methodological limitation that should be considered. As discussed above, the dose-response curve for mesotrione did not fully capture the upper asymptote, resulting in greater uncertainty in the model-derived IC_99_ estimate. Consequently, it cannot be excluded that uncertain IC_99_ concentration contributed, at least in part, to the comparatively weak transcriptional response.

### 4.3 Transcriptomic profiling reflects downstream biological responses instead of primary molecular targets

While RNA-seq enables comprehensive profiling of global gene expression changes following herbicide treatment, it does not uniformly capture the primary molecular targets of all herbicides (Duke et al., 2013). Instead, transcriptomic analyses of herbicide responses predominantly reflect the activation of stress, detoxification, and metabolic pathways that are downstream or secondary to the primary molecular targets. This is expected because many herbicides act through direct biochemical inhibition of specific enzymes or protein complexes, rather than through transcriptional regulation of their target genes (Gaines et al., 2020; Kang et al., 2023). As a result, the primary mode of action may not be directly captured at the transcriptional level (Jugulam and Shyam, 2019; Sen et al., 2023). In this study, the primary molecular target of 2,4-D, the transport inhibitor response 1 (TIR1) and auxin signaling F-box gene family (AFBs) (Dharmasiri et al., 2005), were minimally detected, whereas extensive regulation of numerous genes encoding auxin responsive pathway components such as SAUR-like auxin-responsive protein family and GH3 family proteins was observed (**Supplementary Table S5**, **Supplementary Table S15**). Similarly, for PPO inhibitors, PSII inhibitors, and ALS inhibitors, our RNA-seq primarily captured transcriptional changes associated with secondary impacts on photosynthesis, ribosome biogenesis, and cellular metabolism rather than regulation of the primary target genes (**Figure 1**, **Supplementary Table S5**). Thus, RNA-seq provides a powerful framework for identifying MOA-associated and secondary response pathways, which can be used as a contextual MOA inference tool rather than a good direct primary target detector.

### 4.4 Integration of pairwise experimental design, herbicide unique DEGs, and temporal clustering analysis reveals distinct transcriptional dynamics

As RNA-seq primarily captures transcriptional responses from MOA-associated pathways and broader downstream stress processes, experimental design becomes critical for extracting biologically meaningful insights from transcriptomic analyses (Duke et al., 2013; Giacomini et al., 2018). In this study, a pairwise RNA-seq design was used with two herbicide concentrations (IC_50_ and IC_99_) and two exposure durations (4 h and 24 h) (**Figure 6A**), allowing evaluation of dose-related and temporal transcriptional responses. This design enabled distinction between early/transient responses and progressive or late responses as well as consistent regulatory changes, and also helped separate adaptive stress responses from irreversible, damage-associated transcriptional programs.

To investigate herbicide specificity, analyses were limited to the unique herbicide DEG sets rather than the complete DEG set across treatments (**Figure 7**, **Supplementary Table S6**). By focusing on unique DEGs of each herbicide, it minimized effects from general stress responses and highlighted transcriptional programs that closely reflected herbicide-specific physiological and biochemical perturbations. This strategy enabled clearer identification of MOA-associated and herbicide-specific response pathways, even when the primary molecular targets showed little transcriptional regulation.

Hierarchical clustering was then applied to herbicide unique DEG sets to reveal distinct transcriptional response dynamics across treatments. Clustering was performed using log_2_ fold change values, and gene grouping was based on similarity in their response trajectories across four treatments rather than absolute expression magnitude or direction (up-regulation versus down-regulation) (**Figure 8**, **Supplementary Table S16**). As a result, the color gradients in the cluster heatmaps directly reflected up- or down-regulation of a gene compared to its control (**Figure 8**). This clustering approach revealed recurrent transcriptional response types across treatments, including early/late responsive, progressive, time-related and consistent expression patterns, which could not be detected from single time-point analyses or DEG lists alone. Importantly, integration of functional enrichment analysis with these expression clusters revealed dominant biological pathways associated with specific transcriptional response patterns (**Figure 8**, **Supplementary Figures S3–S9**). For example, auxin response was predominantly enriched within consistently induced cluster 2 and 3 under 2,4-D treatment (**Figure 8A**, **Supplementary Figure S3**), indicating a characteristic temporal transcriptional signature linked to 2,4-D. Moreover, similar biological themes sometimes exhibited distinct or conserved transcriptional expression patterns across different herbicide treatments. For example, ribosome biogenesis-related pathways displayed divergent expression dynamics in atrazine and imazethapyr, while photosynthesis repression-associated pathways showed similar expression patterns in acifluorfen and imazethapyr (**Figure 8**, **Table 2**, **Supplementary Table S15**, and **Supplementary Figures S4, S5, S7**). These similarities and differences may reflect variations in herbicide chemistry, primary molecular targets, and the downstream physiological consequences resulting from target inhibition. Comparative analysis of these dominant functional themes across herbicides further revealed that herbicides converged on similar broader biological effects, such as stress response-, growth/cell-cycle inhibition-, and detoxification-related processes despite largely nonoverlapping gene sets and varied significant functional terms (**Table 2**). Together, these results demonstrated that hierarchical clustering-based analysis of unique DEGs provided a powerful framework for linking transcriptional dynamics to herbicide-specific and shared biological effects even when the underlying genes shared little overlap.

### 4.5 Limitations and future perspectives

While this study provides comprehensive insights into herbicide-induced physiological and transcriptional responses, several limitations should be considered. First, this analysis of molecular responses was based exclusively on transcriptomic data. Integration of additional omics approaches, such as metabolomics and proteomics, would provide deeper insights into metabolic and protein-level changes underlying the comparable levels of growth inhibition, particularly for herbicides that elicit relatively subtle transcriptional changes or delayed transcriptional responses. For example, previous studies on glyphosate (Mesnage et al., 2018) demonstrated that metabolomic profiling following transcriptomic analysis revealed a decrease in the level of free long chain fatty acids and polyunsaturated fatty acids, which were not detected at the transcriptional level. Second, this work focused on a single model species, *Arabidopsis thaliana*. Extending similar analyses to multiple plant species would allow evaluation of the conservation and divergence of herbicide response signatures across species (Das et al., 2010). Third, this study was limited by the collection of samples at only two time points post-treatment. Herbicides differ in the rate at which they exert their physiological effects, with some acting fast, such as paraquat and acifluorfen, while others, such as glyphosate in this study, act more slowly. Thus, fixed sampling times may not represent equal stages of responses across herbicides in comparative transcriptomic analysis, even if the IC_50_ and IC_99_ concentration effects are compared. This limitation may partially account for differences in transcriptional profiles, as glyphosate induced limited transcriptional changes at early time point while the same timepoint for fast acting herbicides already reflected secondary stress related responses. Future research using tailored time-course sampling will allow for a more detailed comparison of individual herbicide modes of action. Fourth, this study investigated a single herbicide for each mode of action class, future studies incorporating multiple herbicides within the same MOA class would allow identification of transcriptomic signatures arising from compound specificity (Das et al., 2010). Finally, parallel transcriptomic analyses of resistant and susceptible genotypes would provide a powerful approach for directly linking transcriptional responses to resistance mechanisms (Chen et al., 2024). Collectively, these future directions would further enhance the utility of transcriptomic profiling for herbicide MOA characterization and resistance research.

## Data Availability

The original contributions presented in the study are publicly available. The RNA-seq raw data are publicly available in the NCBI Gene Expression Omnibus (GEO) database under accession number GSE327091.
