# Supplementary material for: Dose-response-defined physiological framework enables comparative analysis of transcriptional response dynamics across multiple herbicides in Arabidopsis thaliana
Source: Front Plant Sci. 2026 Sep 23;17:1870671. doi: 10.3389/fpls.2026.1870671 (PMC13642330; doi:10.3389/fpls.2026.1870671)
Supplement: Supplementary file 1 [file DataSheet1.pdf]

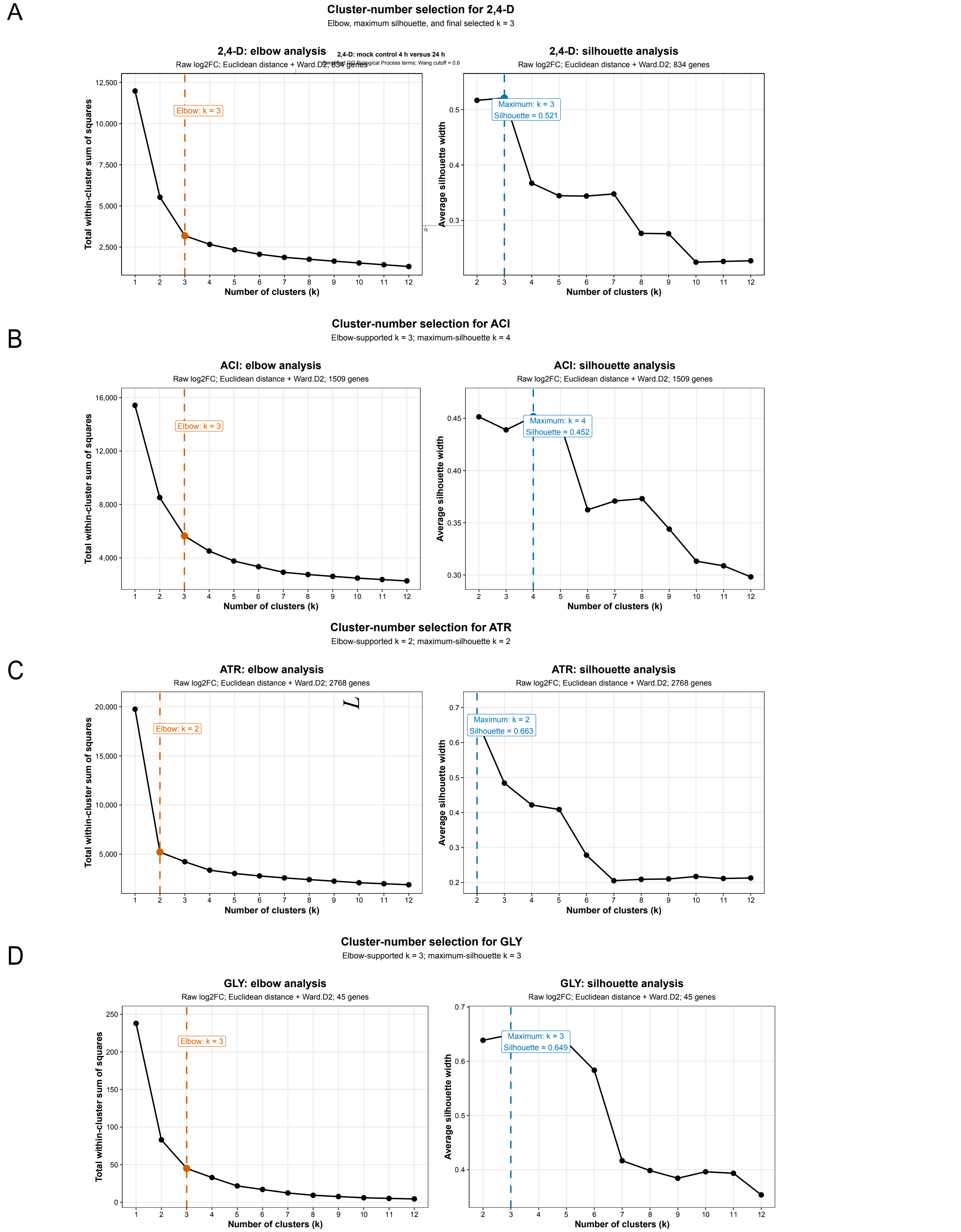

Supplementary Figure S1. Selection of cluster numbers (*k*) for hierarchical clustering of herbicide unique DEGs. Cluster number was evaluated separately for (A) 2,4-D, (B) ACI: acifluorfen, (C) ATR: atrazine, (D) GLY: glyphosate. For each herbicide, hierarchical clustering was performed using Euclidean distance and Ward.D2 linkage, and the resulting dendrogram was evaluated over candidate cluster numbers. Total within-cluster sum of squares (WSS) was calculated for $k = 1\text{-}12$, and average silhouette width was calculated for $k = 2\text{-}12$ using the same Euclidean-distance matrix. When the elbow and maximum-silhouette criteria indicated different values, the final cluster number was determined by jointly considering changes in WSS, average silhouette width, cluster-size distribution, and whether additional clusters represented distinct expression profiles. Based on these criteria, $k = 3$, 4, 2, and 2 were selected for 2,4-D, acifluorfen, atrazine, and glyphosate, respectively.

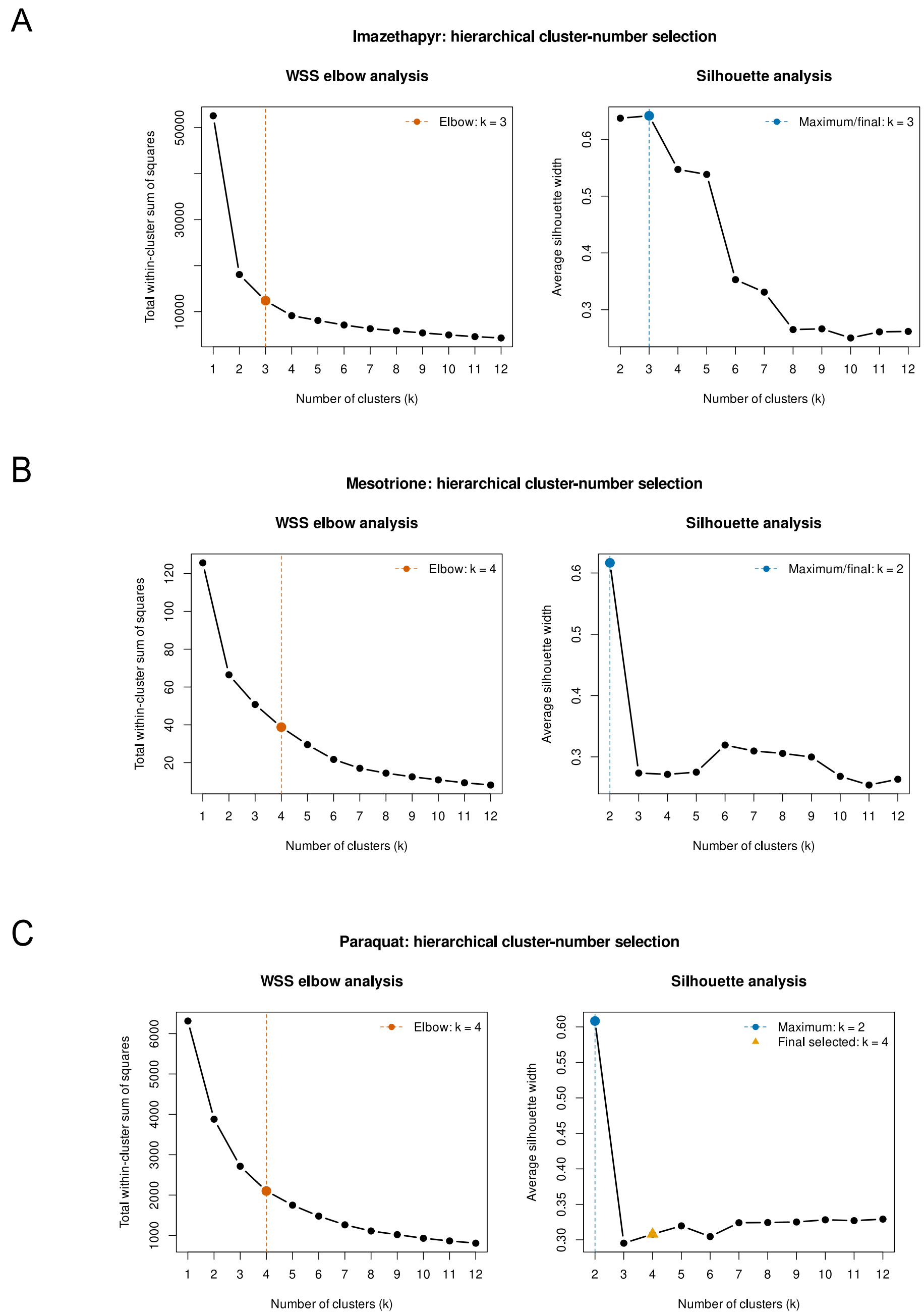

Supplementary Figure S2. Selection of cluster numbers (*k*) for hierarchical clustering of herbicide unique DEGs. Cluster number was evaluated separately for (A) imazethapyr, (B) mesotrione, (C) paraquat. For each herbicide, hierarchical clustering was performed using Euclidean distance and Ward.D2 linkage, and the resulting dendrogram was evaluated over candidate cluster numbers. Total within-cluster sum of squares (WSS) was calculated for k = 1-12, and average silhouette width was calculated for k = 2-12 using the same Euclidean-distance matrix. When the elbow and maximum-silhouette criteria indicated different values, the final cluster number was determined by jointly considering changes in WSS, average silhouette width, cluster-size distribution, and whether additional clusters represented distinct expression profiles. Based on these criteria, k = 3, 2, and 4 were selected for imazethapyr, mesotrione, paraquat, respectively.

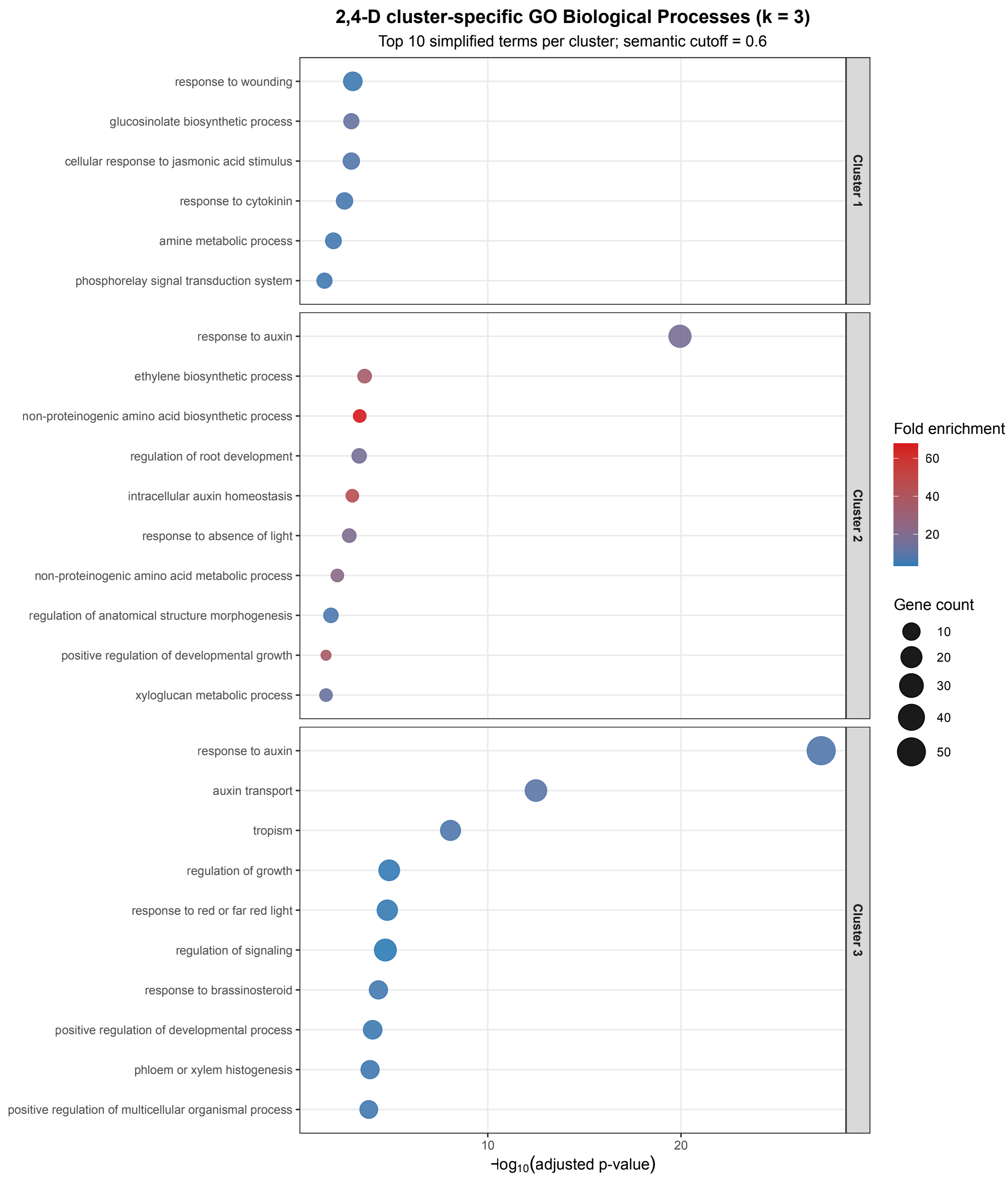

Supplementary Figure S3. Cluster-specific GO-BP enrichment analysis of 2,4-D unique DEGs. GO-BP enrichment was performed separately for each hierarchical gene cluster as described in Methods section. For visualization, semantically redundant terms were reduced within each cluster, and the terms with the smallest adjusted *p* value was retained as the representatives. Up to 10 of the most significant simplified terms per cluster were displayed. The *x*-axis represents $-\log_{10}$(adjusted *p* value), dot size indicates gene count, and dot color represents fold enrichment. Cluster numbers correspond to their top-to-bottom positions in the heatmap in Figure 8A. Complete unsimplified enrichment results are provided in Supplementary Table S10.

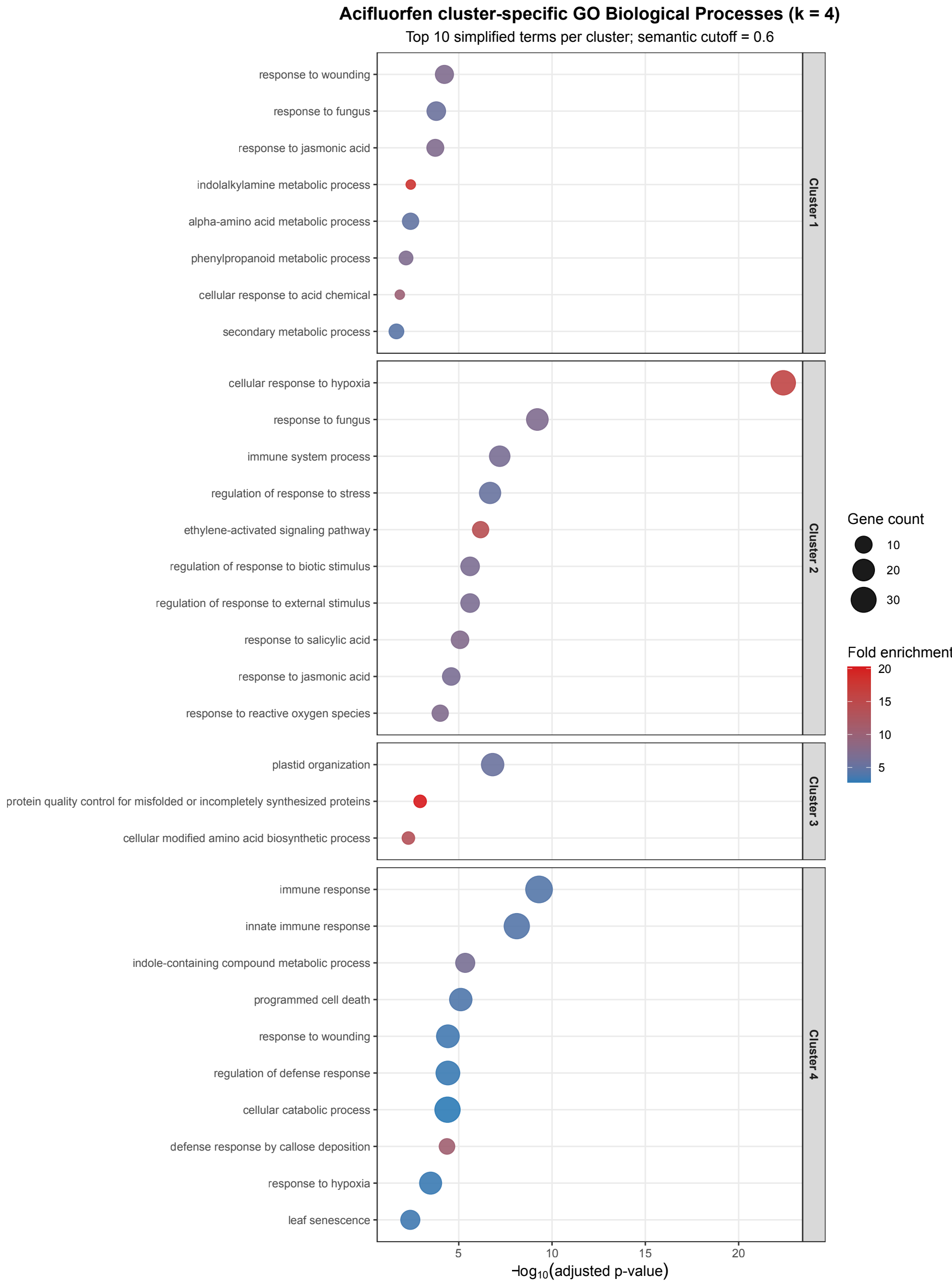

Supplementary Figure S4. Cluster-specific GO-BP enrichment analysis of Acifluorfen unique DEGs. GO-BP enrichment was performed separately for each hierarchical gene cluster as described in Methods section. For visualization, semantically redundant terms were reduced within each cluster, and the terms with the smallest adjusted *p* value was retained as the representatives. Up to 10 of the most significant simplified terms per cluster were displayed. The *x*-axis represents $-\log_{10}$(adjusted *p* value), dot size indicates gene count, and dot color represents fold enrichment. Cluster numbers correspond to their top-to-bottom positions in the corresponding heatmap in Figure 8B. Complete unsimplified enrichment results are provided in Supplementary Table S10.

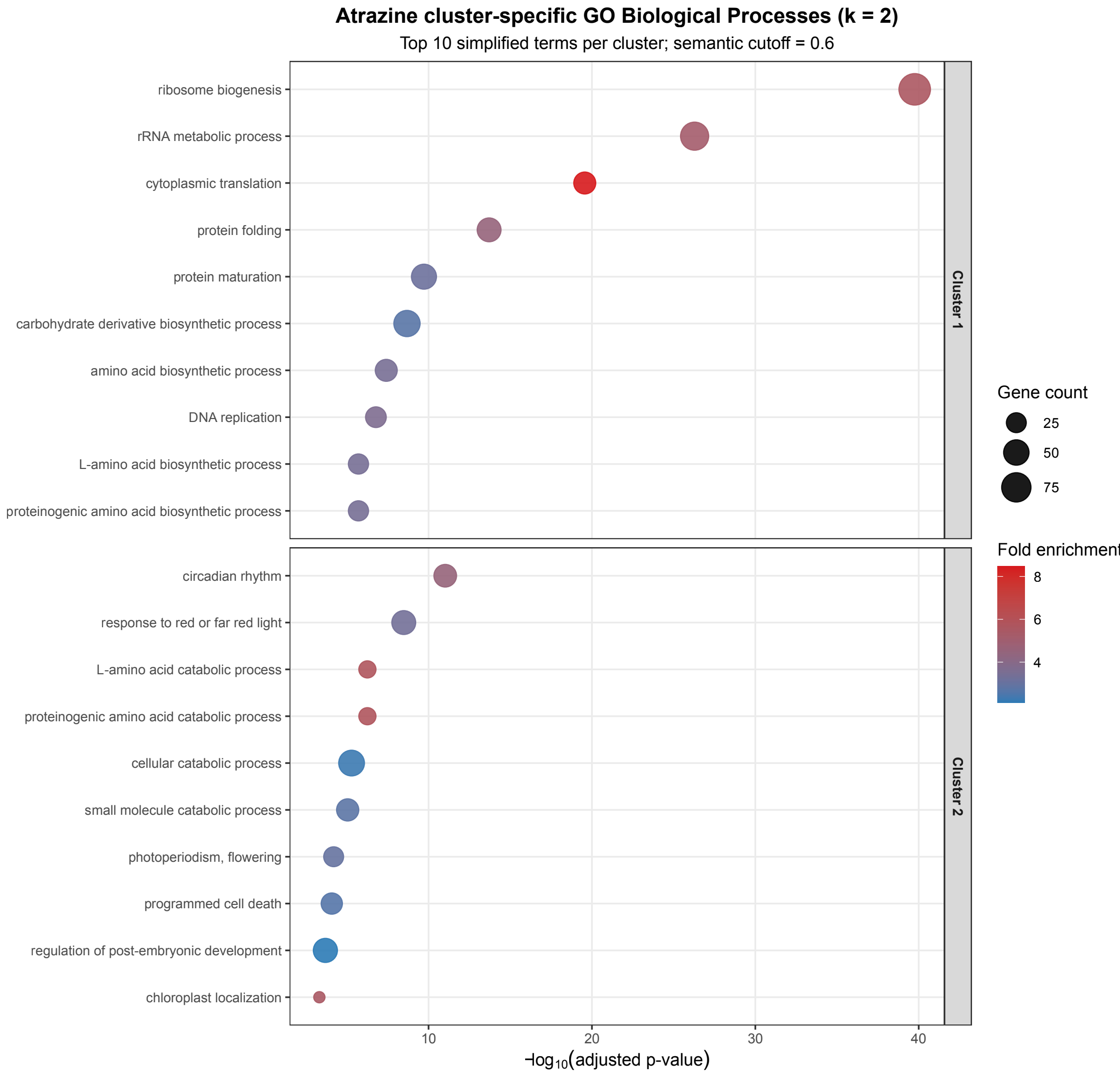

Supplementary Figure S5. Cluster-specific GO-BP enrichment analysis of Atrazine unique DEGs. GO-BP enrichment was performed separately for each hierarchical gene cluster as described in Methods section. For visualization, semantically redundant terms were reduced within each cluster, and the terms with the smallest adjusted *p* value was retained as the representatives. Up to 10 of the most significant simplified terms per cluster were displayed. The *x*-axis represents $-\log_{10}$(adjusted *p* value), dot size indicates gene count, and dot color represents fold enrichment. Cluster numbers correspond to their top-to-bottom positions in the corresponding heatmap in Figure 8C. Complete unsimplified enrichment results are provided in Supplementary Table S10.

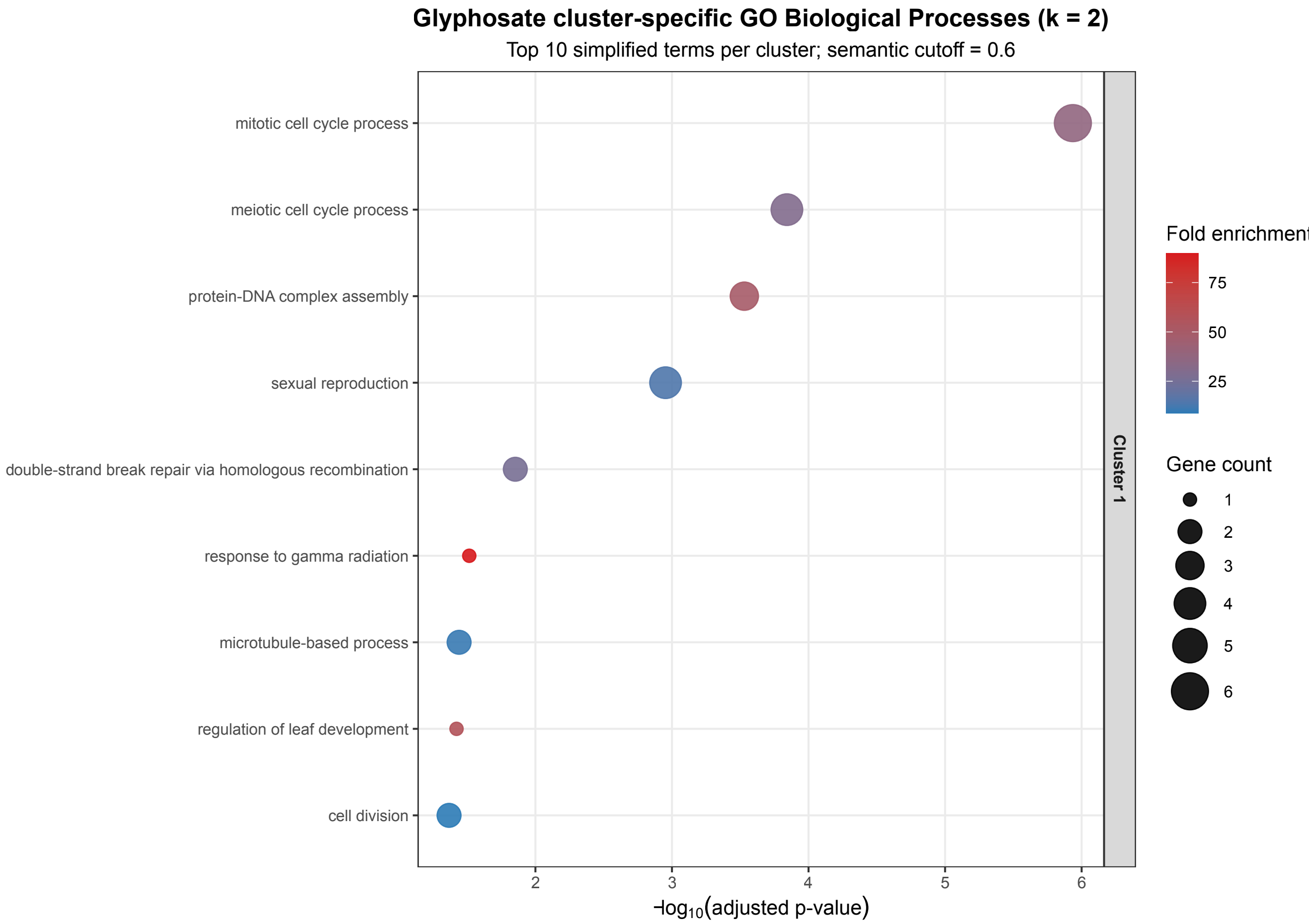

Supplementary Figure S6. Cluster-specific GO-BP enrichment analysis of Glyphosate unique DEGs. GO-BP enrichment was performed separately for each hierarchical gene cluster as described in Methods section. For visualization, semantically redundant terms were reduced within each cluster, and the terms with the smallest adjusted *p* value was retained as the representatives. Up to 10 of the most significant simplified terms per cluster were displayed. The *x*-axis represents $-\log_{10}$(adjusted *p* value), dot size indicates gene count, and dot color represents fold enrichment. Cluster numbers correspond to their top-to-bottom positions in the corresponding heatmap in Figure 8D. Complete unsimplified enrichment results are provided in Supplementary Table S10.

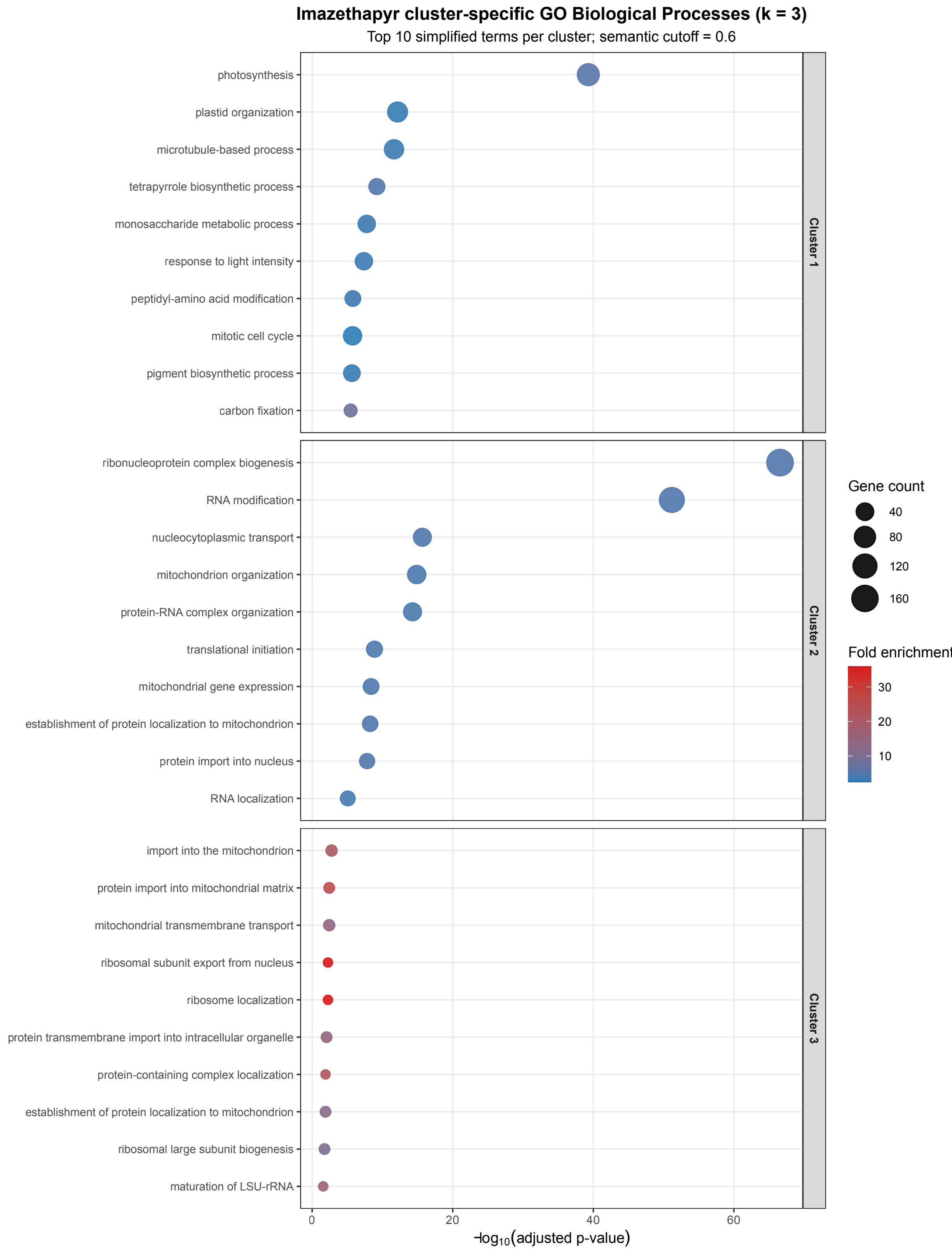

Supplementary Figure S7. Cluster-specific GO-BP enrichment analysis of Imazethapyr unique DEGs. GO-BP enrichment was performed separately for each hierarchical gene cluster as described in Methods section. For visualization, semantically redundant terms were reduced within each cluster, and the terms with the smallest adjusted *p* value was retained as the representatives. Up to 10 of the most significant simplified terms per cluster were displayed. The *x*-axis represents $-\log_{10}$(adjusted *p* value), dot size indicates gene count, and dot color represents fold enrichment. Cluster numbers correspond to their top-to-bottom positions in the corresponding heatmap in Figure 8E. Complete unsimplified enrichment results are provided in Supplementary Table S10.

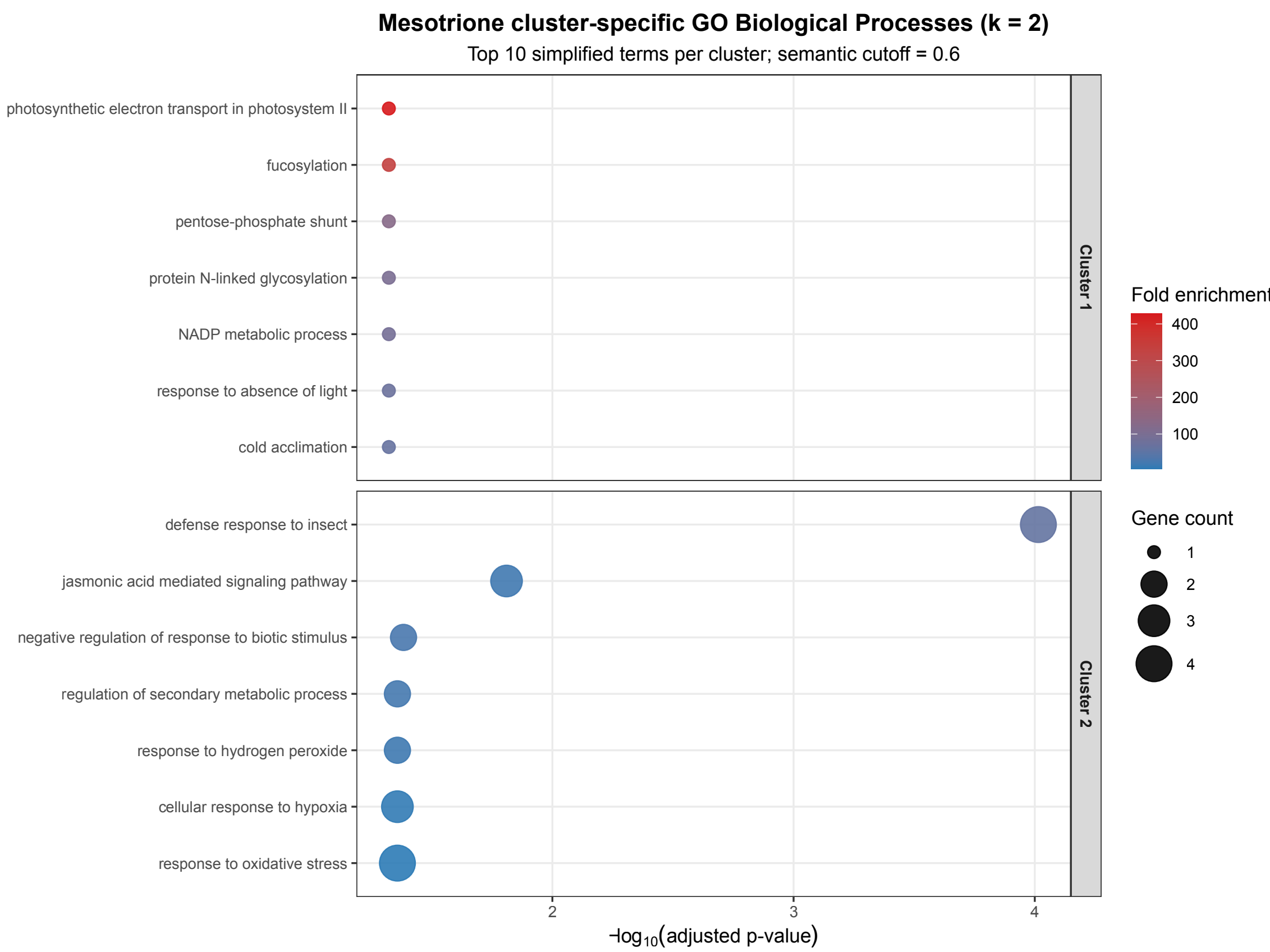

Supplementary Figure S8. Cluster-specific GO-BP enrichment analysis of Mesotrione unique DEGs. GO-BP enrichment was performed separately for each hierarchical gene cluster as described in Methods section. For visualization, semantically redundant terms were reduced within each cluster, and the terms with the smallest adjusted *p* value was retained as the representatives. Up to 10 of the most significant simplified terms per cluster were displayed. The *x*-axis represents $-\log_{10}$(adjusted *p* value), dot size indicates gene count, and dot color represents fold enrichment. Cluster numbers correspond to their top-to-bottom positions in the corresponding heatmap in Figure 8F. Complete unsimplified enrichment results are provided in Supplementary Table S10.

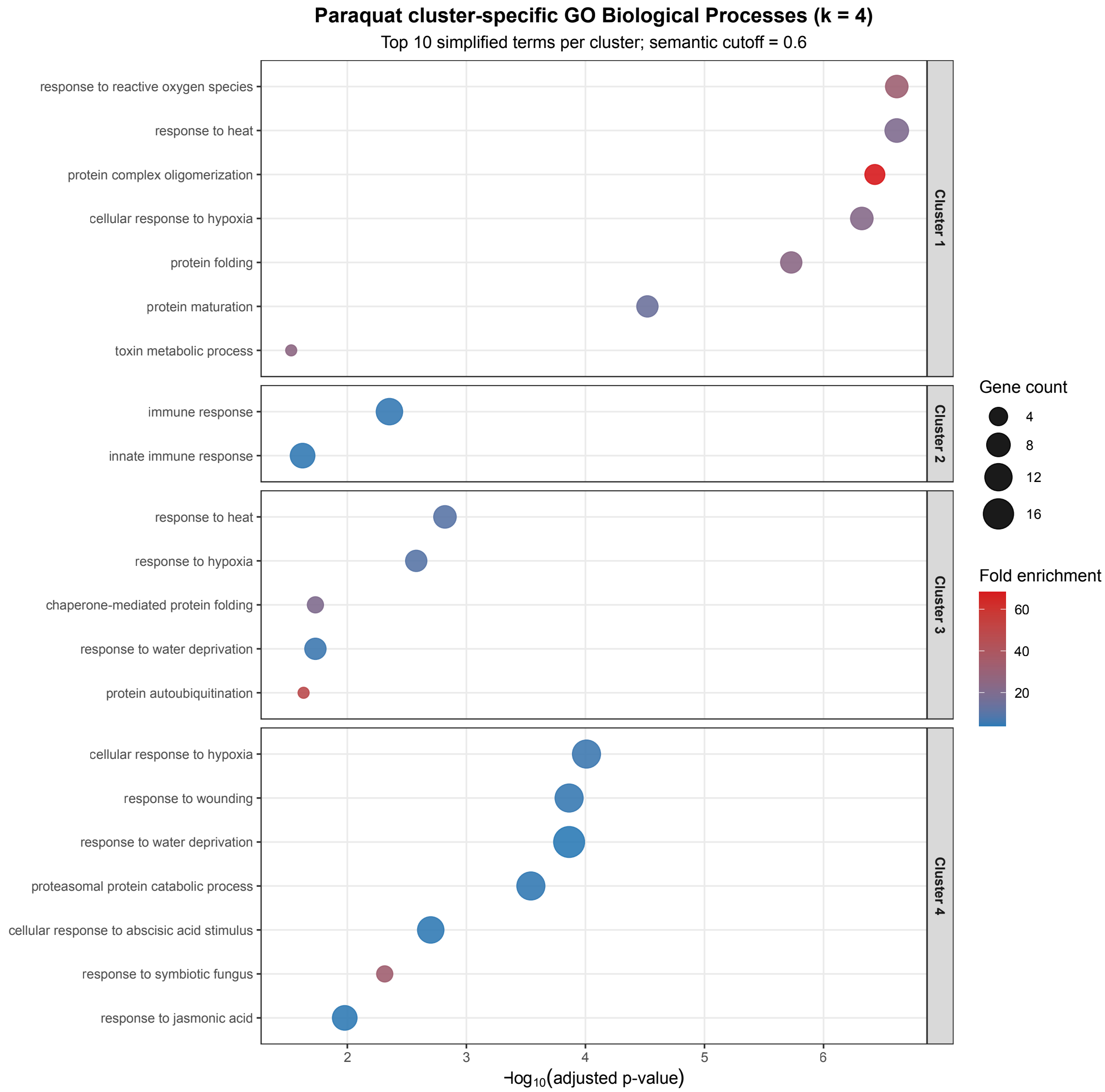

Supplementary Figure S9. Cluster-specific GO-BP enrichment analysis of Paraquat unique DEGs. GO-BP enrichment was performed separately for each hierarchical gene cluster as described in Methods section. For visualization, semantically redundant terms were reduced within each cluster, and the terms with the smallest adjusted *p* value was retained as the representatives. Up to 10 of the most significant simplified terms per cluster were displayed. The *x*-axis represents $-\log_{10}$(adjusted *p* value), dot size indicates gene count, and dot color represents fold enrichment. Cluster numbers correspond to their top-to-bottom positions in the corresponding heatmap in Figure 8G. Complete unsimplified enrichment results are provided in Supplementary Table S10.

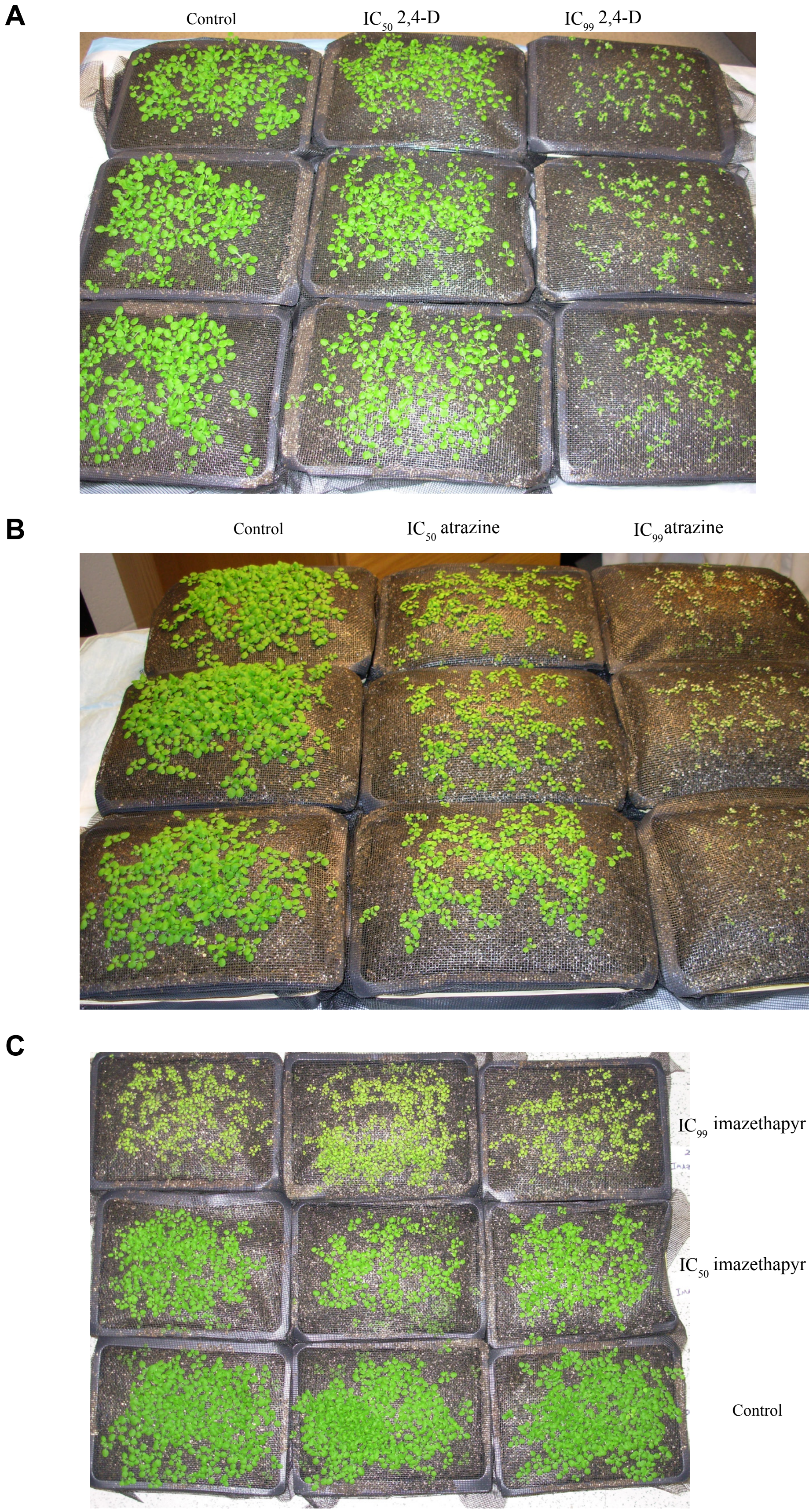

Supplementary Figure S10. Physiological effects of representative herbicide treatments at estimated IC_{50} and IC_{99}. Phenotypic comparison in growth inhibition across three representative herbicides: (A) 2,4-D; (B) atrazine; and (C) imazethapyr, at moderate (IC_{50}) and severe (IC_{99}) levels of physiological perturbations. Images were taken four days after treatment, showing distinct herbicide-specific phenotypic responses.

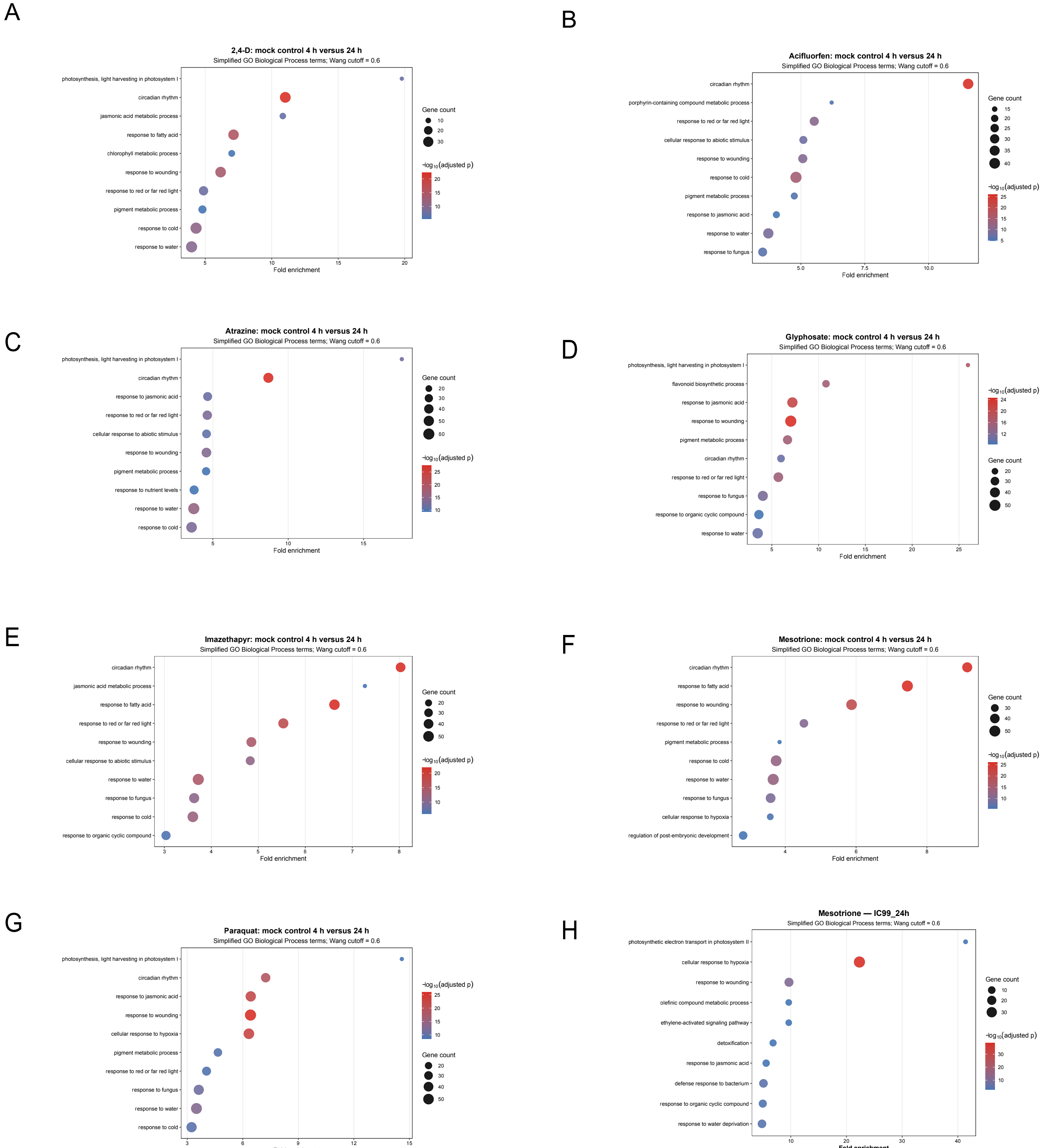

Supplementary Figure S11. GO enrichment analysis of temporal changes between mock-treated controls and comparison with the mesotrione treatment response. Biological Process GO enrichment was performed using DEGs identified between mock-treated control samples collected at 4 h and 24 h. Following semantic simplification, top 10 of the most significantly enriched terms were selected based on the lowest adjusted *p* values. Panels show results for (A) 2,4-D, (B) acifluorfen, (C) atrazine, (D) glyphosate, (E) imazethapyr, (F) mesotrione, and (G) paraquat treatments. The top enriched terms are predominantly associated with circadian rhythm and light-related processes. For comparison, (H) shows GO-BP enrichment of mesotrione-responsive DEGs at IC_{99}_24 h, which is dominated by stress-response processes rather than the circadian-rhythm terms observed in the corresponding mesotrione mock-control comparison in (F). The *x*-axis represents fold enrichment, dot size indicates gene count, and color represents $-\log_{10}$(adjusted *p* value).

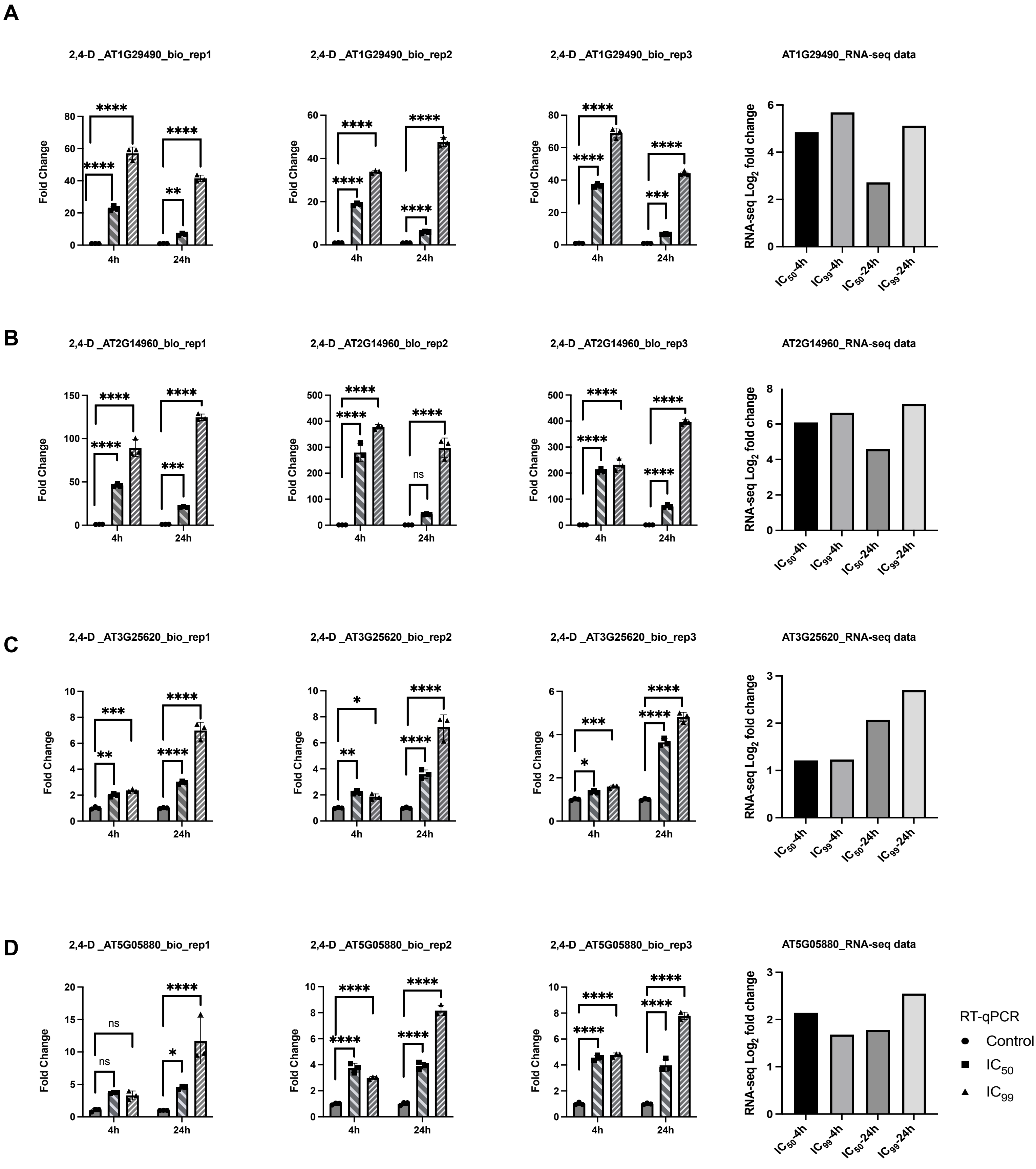

Supplementary Figure S12. RT-qPCR validation across all biological replicates under 2,4-D treatment. Relative expression levels of four representative genes (A-D) under 2,4-D treatment across four conditions are shown for three independent biological replicates. Last column shows RNA-seq $\log_2$ fold change of each representative gene across four conditions. Overall similar expression patterns are observed across biological replicates, and is consistent with the RNA-seq results.

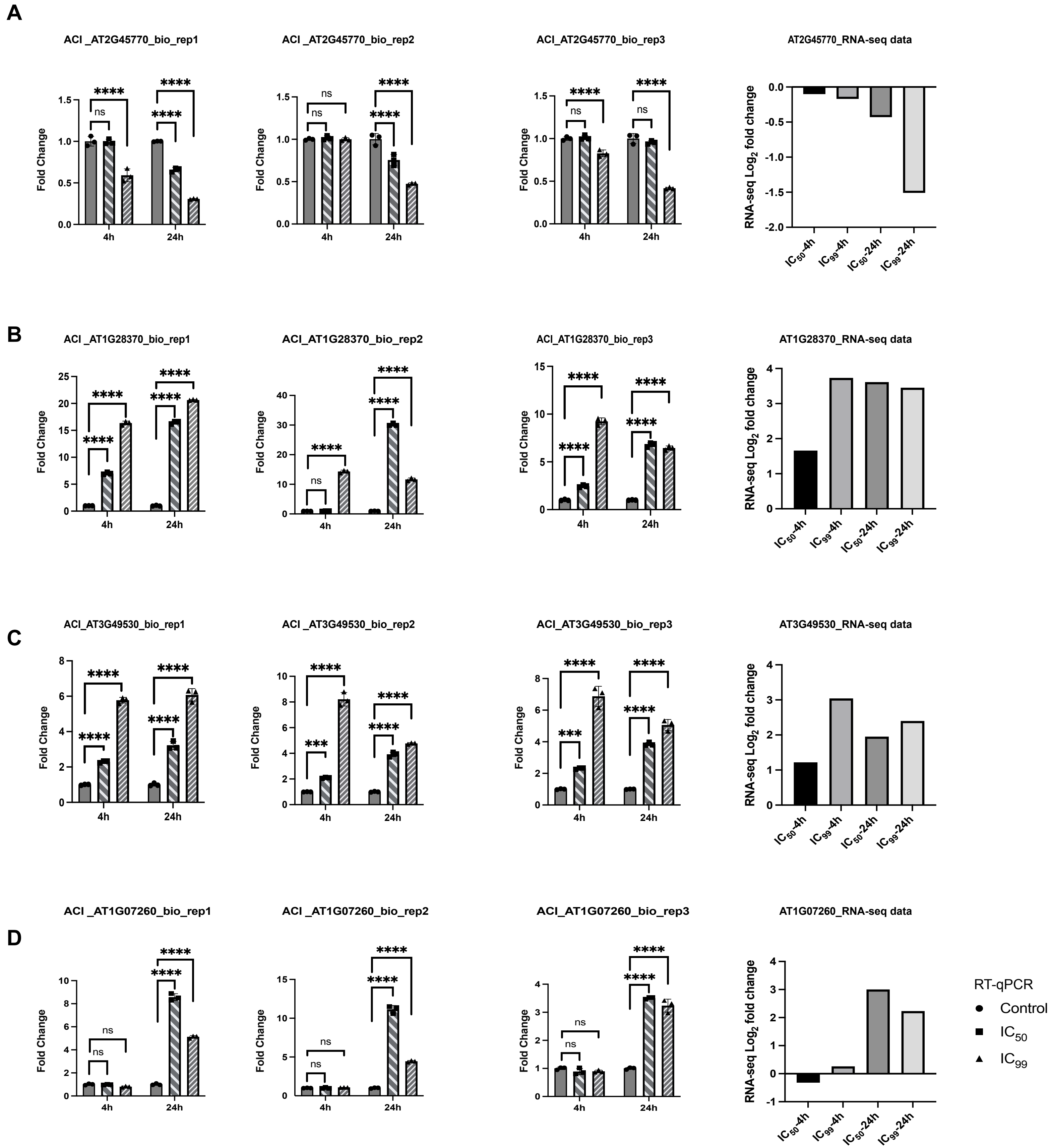

Supplementary Figure S13. RT-qPCR validation across all biological replicates under acifluorfen (ACI) treatment. Relative expression levels of four representative genes (A-D) under acifluorfen treatment across four conditions are shown for three independent biological replicates. Last column shows RNA-seq log_2 fold change of each representative gene across four conditions. Overall similar expression patterns are observed across biological replicates, and is consistent with the RNA-seq results.

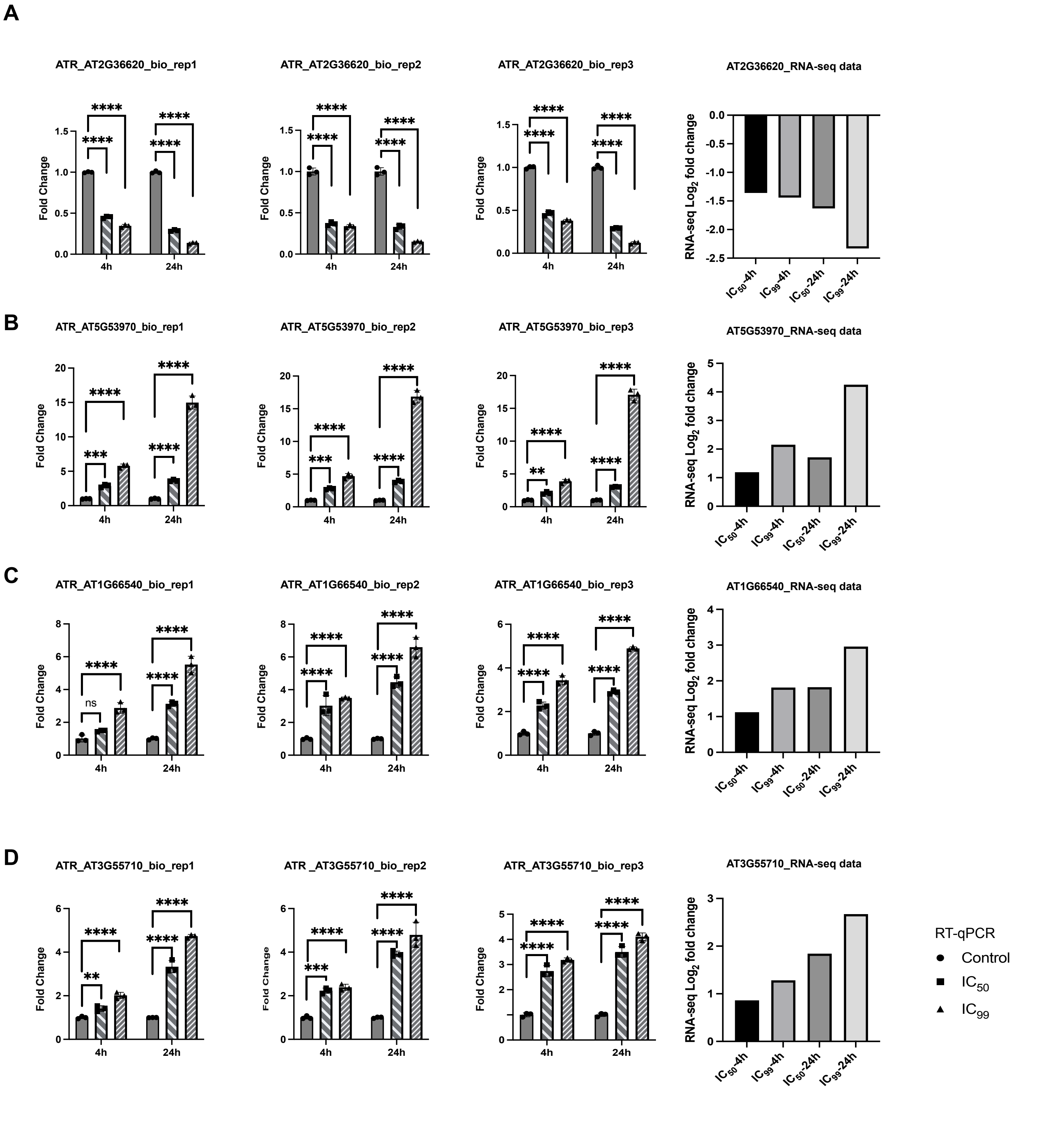

Supplementary Figure S14. RT-qPCR validation across all biological replicates under atrazine (ATR) treatment. Relative expression levels of four representative genes (A-D) under atrazine treatment across four conditions are shown for three independent biological replicates. Last column shows RNA-seq $\log_2$ fold change of each representative gene across four conditions. Overall similar expression patterns are observed across biological replicates, and is consistent with the RNA-seq results.

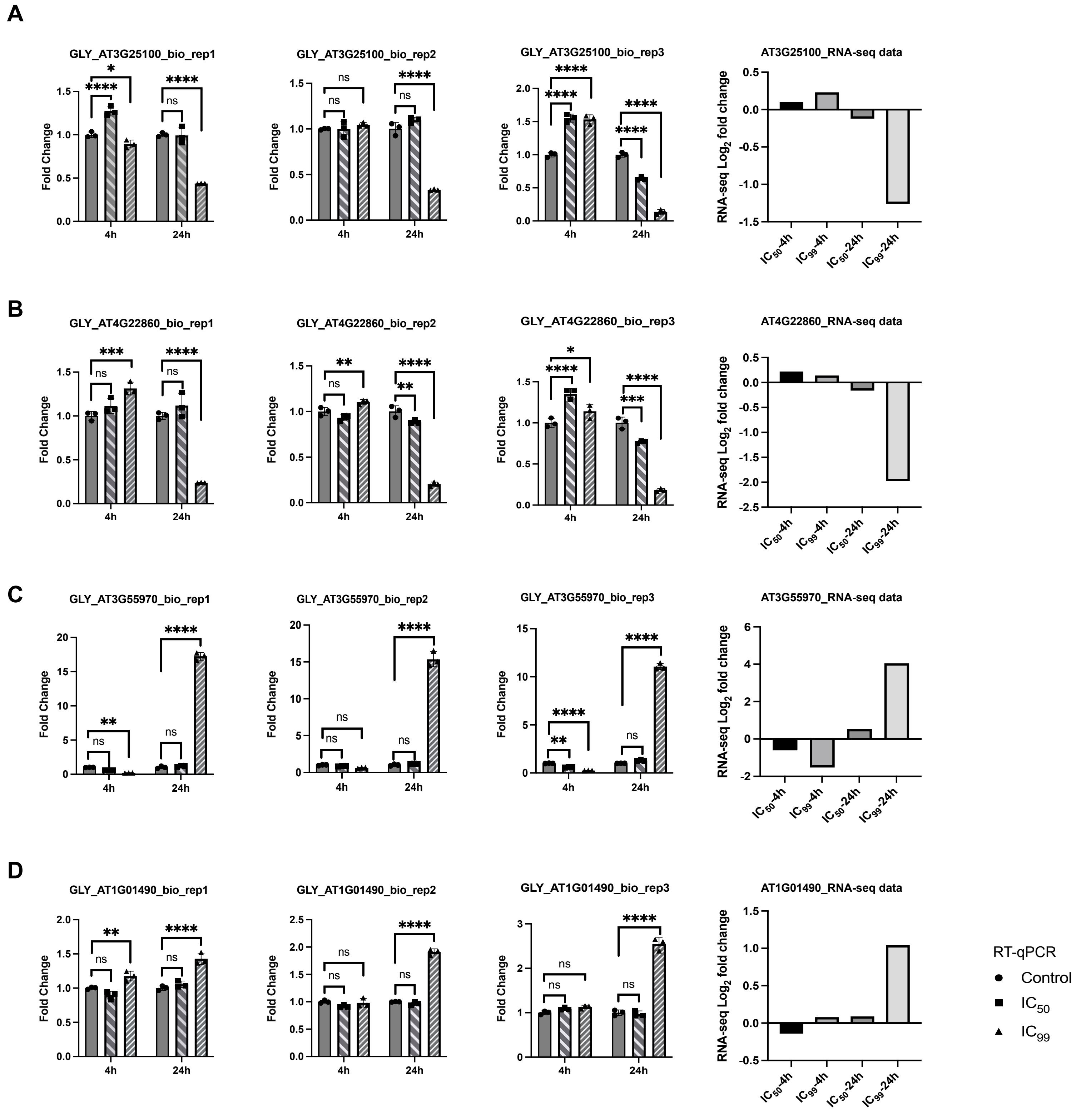

Supplementary Figure S15. RT-qPCR validation across all biological replicates under glyphosate (GLY) treatment. Relative expression levels of four representative genes (A-D) under glyphosate treatment across four conditions are shown for three independent biological replicates. Last column shows RNA-seq $\log_2$ fold change of each representative gene across four conditions. Overall similar expression patterns are observed across biological replicates, and is consistent with the RNA-seq results.

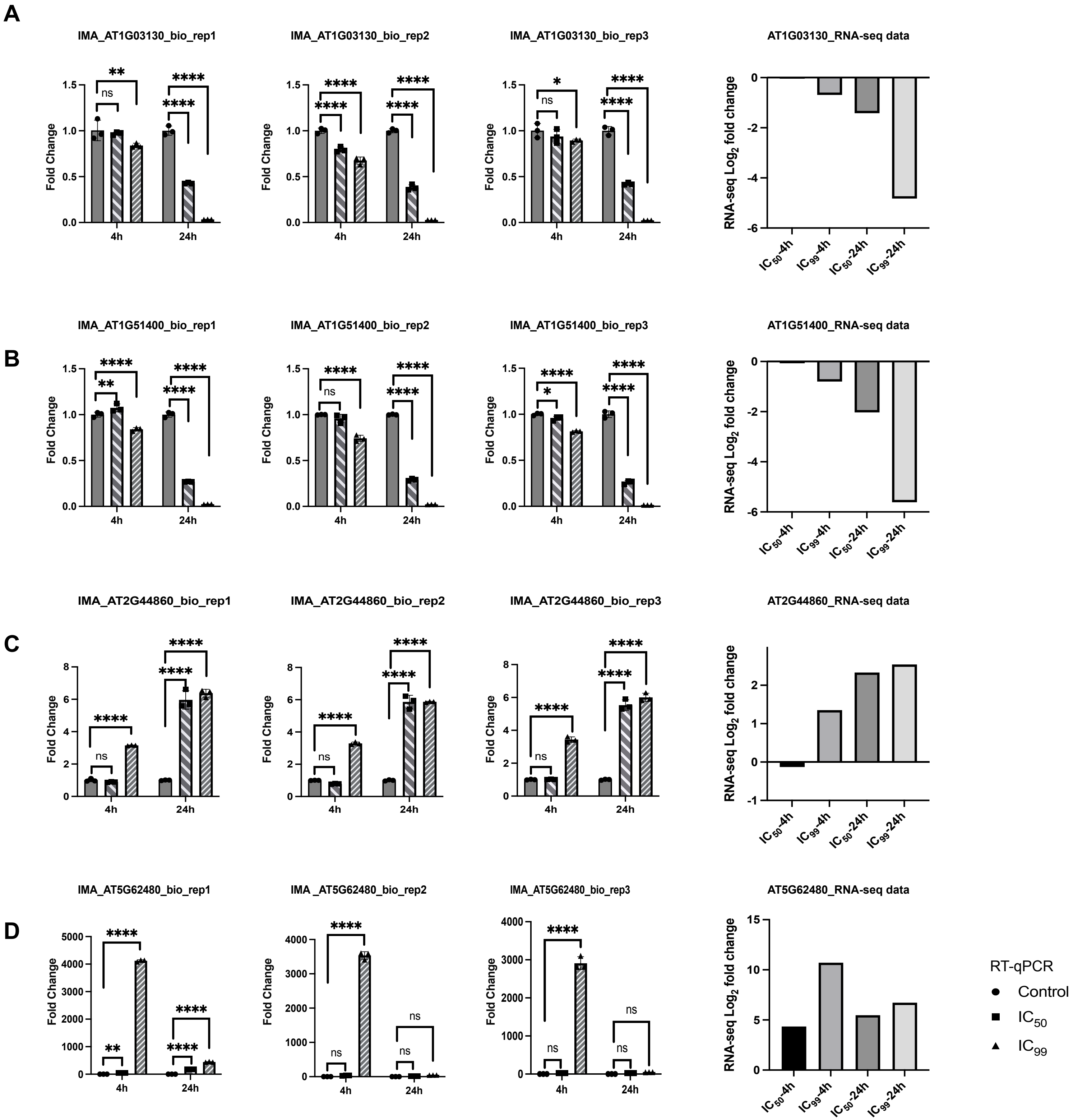

Supplementary Figure S16. RT-qPCR validation across all biological replicates under imazethapyr (IMA) treatment. Relative expression levels of four representative genes (A-D) under imazethapyr treatment across four conditions are shown for three independent biological replicates. Last column shows RNA-seq $\log_2$ fold change of each representative gene across four conditions. Overall similar expression patterns are observed across biological replicates, and is consistent with the RNA-seq results.

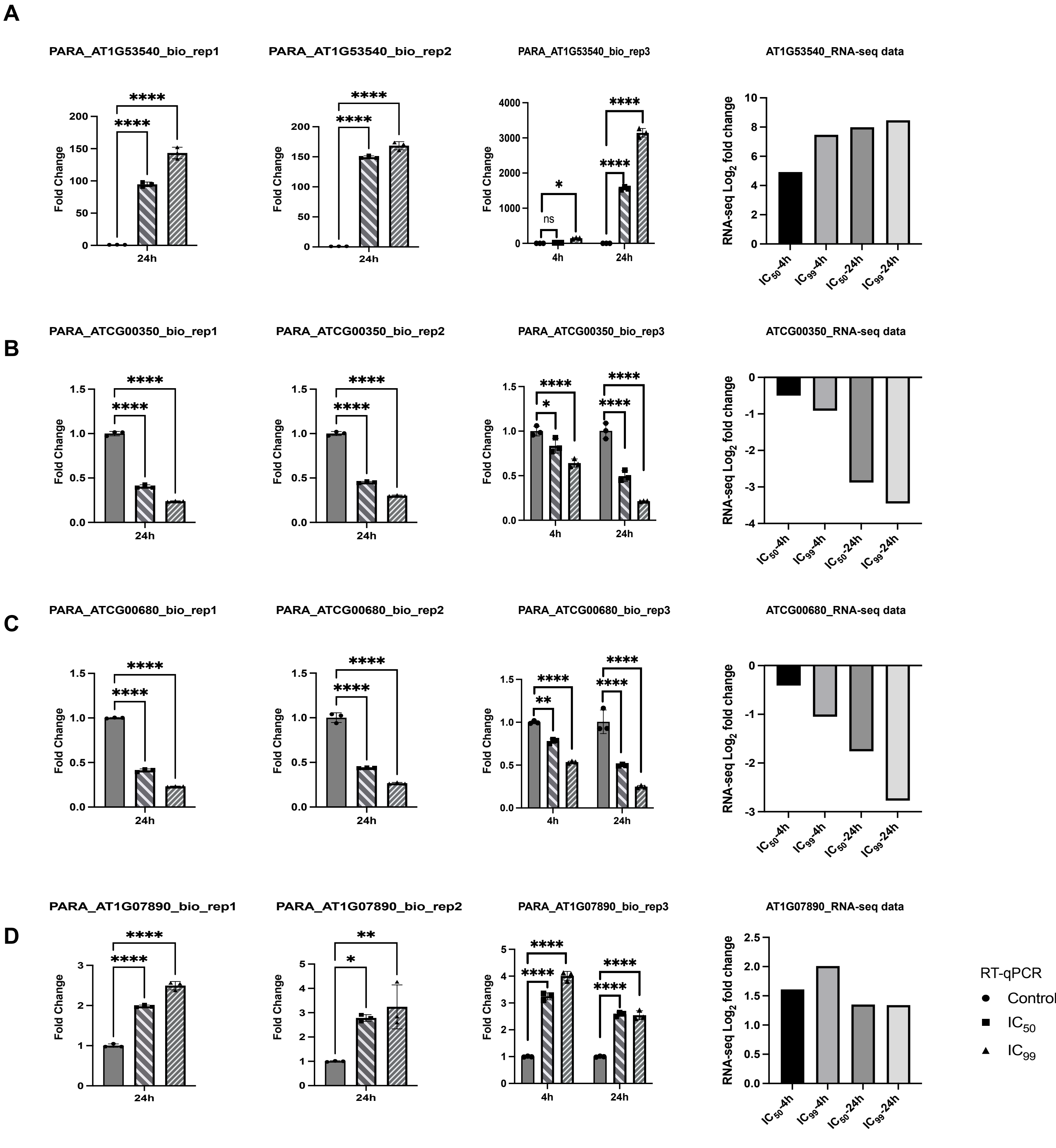

Supplementary Figure S17. RT-qPCR validation across all biological replicates under paraquat (PARA) treatment. Relative expression levels of four representative genes (A-D) under paraquat treatment across four conditions are shown for three independent biological replicates. Due to limited RNA samples availability, only biological replicate 3 includes complete data for 4 h and 24 h treatments at both dosages, whereas biological replicate 1 and 2 include data for 24 h treatment only. The 24 h expression patterns are consistent across all biological replicates. Last column shows RNA-seq $\log_2$ fold change of each representative gene across four conditions.

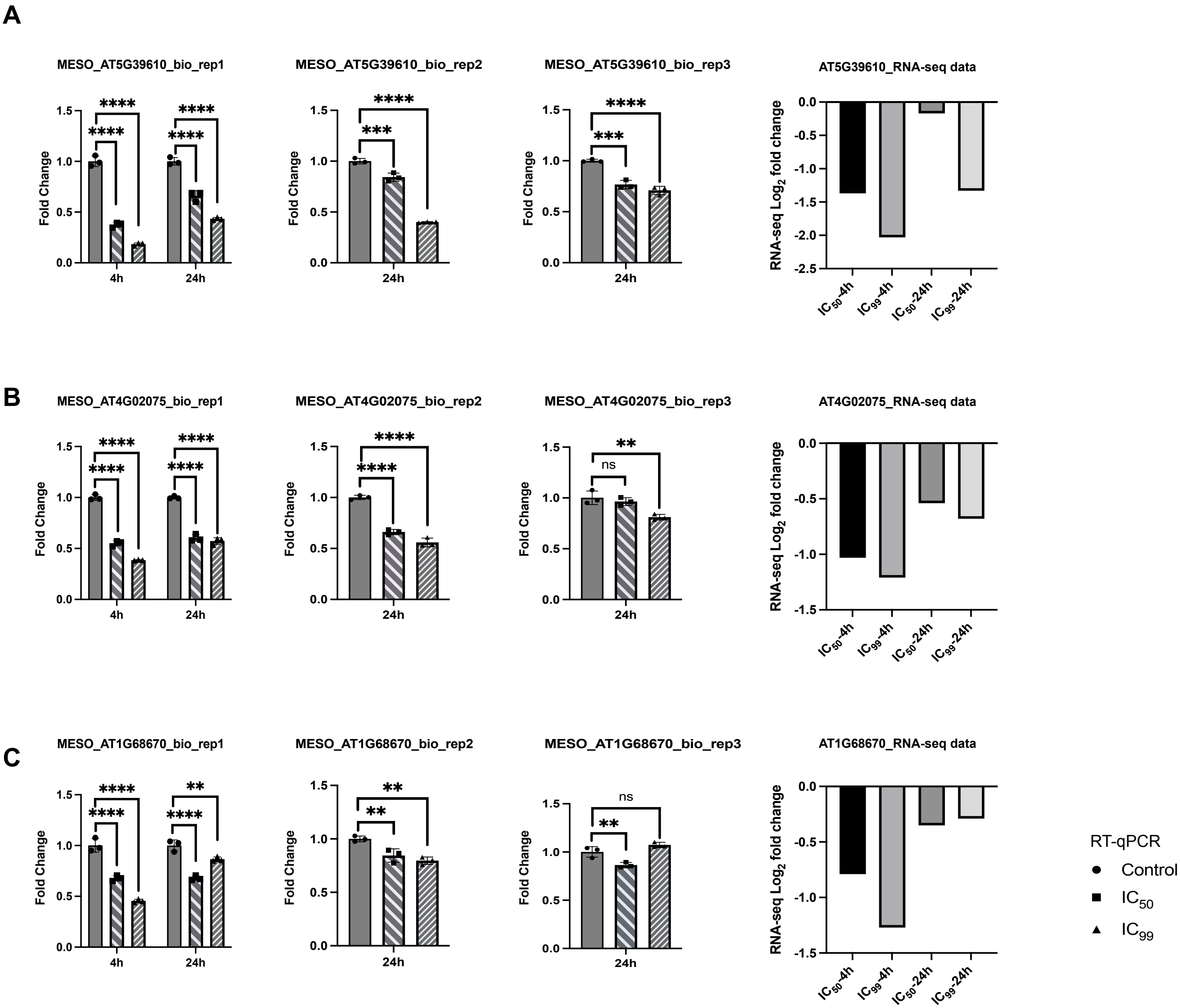

Supplementary Figure S18. RT-qPCR validation across all biological replicates under mesotrione (MESO) treatment. Relative expression levels of three representative genes (A-C) under mesotrione treatment across four conditions are shown for three independent biological replicates. Due to limited RNA samples availability, only biological replicate 1 includes data for 4 h and 24 h treamtns at both dosages, whereas biological replicate 2 and 3 include data for 24 h treatment only. The 24 h expression patterns are consistent across all biological replicates. Last column shows RNA-seq log_2 fold change of each representative gene across four conditions.
