# Supplementary material for: Dose-response-defined physiological framework enables comparative analysis of transcriptional response dynamics across multiple herbicides in Arabidopsis thaliana
Source: Front Plant Sci. 2026 Sep 23;17:1870671. doi: 10.3389/fpls.2026.1870671 (PMC13642330; doi:10.3389/fpls.2026.1870671)
Supplement: Supplementary file 2 [file DataSheet2.zip › Supplementary Tables/Supplementary Table S1.docx]

**Supplementary Table S1**. Herbicide concentrations tested in dose-response experiments.

| **Herbicide** | **Tested Concentrations (μM)** |
| --- | --- |
| **2,4-D** | 0, 5.5, 17, 50, 150, 450 |
| **Acifluorfen** | 0, 0.3, 1.5, 7.5, 37.5, 75, 150, 300 |
| **Atrazine** | 0, 31.25, 62.5, 125, 250, 500 |
| **Glyphosate** | 0, 3.3, 10, 33, 100, 333 |
| **Imazethapyr** | 0, 0.37, 1.1, 3.3, 10, 30 |
| **Mesotrione** | 0, 0.1, 0.5, 2.5, 12.5, 62.5 |
| **Paraquat** | 0, 2.22, 6.67, 20, 60, 180 |
