# Supplementary material for: Dose-response-defined physiological framework enables comparative analysis of transcriptional response dynamics across multiple herbicides in Arabidopsis thaliana
Source: Front Plant Sci. 2026 Sep 23;17:1870671. doi: 10.3389/fpls.2026.1870671 (PMC13642330; doi:10.3389/fpls.2026.1870671)
Supplement: Supplementary file 2 [file DataSheet2.zip › Supplementary Tables/Supplementary Table S12.docx]

**Supplementary Table S12**: Target genes and reference gene with PCR primers used in real-time qPCR assays.

| **Herbicide Treatment** | **Category of Genes to Test** | **Genes to Test** | **PCR primers** |
| --- | --- | --- | --- |
| 2,4-D | MOA-associated & cluster representative (C2) | AT1G29490 | F: CTCATGAAGATGGCCAAGAAATG  R: CGTACTTGATCCGCAGTGTAA |
|  | MOA-associated & cluster representative (C2) | AT2G14960 | F: CTCACTAGCTCTGGAACATCAG  R: GGCATGAGGAGACTGTAAAGAA |
|  | shared function theme | AT3G25620 | F: CACAAGACGATGTTCTCTACCC  R: CATCTCCACCTGCTCTAGTTTC |
|  | shared function theme | AT5G05880 | F: TTTGTGAAGGCGTCCCTATG  R: AATCCGACCCTCGAGATGTA |
| Acifluorfen | MOA-associated | AT2G45770 | F: CTCTTGGAAAGCTGGCTCATA  R: GTCCTCTCTGCCCATATCTCTA |
|  | cluster representative (C2) | AT1G28370 | F: ATTCAGGTTAAGGCTGGGATG  R: CCACACGTCGTCCTTCATATC |
|  | cluster representative (C2) | AT3G49530 | F: CACGCTGTTCTCTCCATTACA  R: GGAAGCTGTTGTTGTTGTTCC |
|  | shared function theme | AT1G07260 | F: GGGTCGAGATTGCAGAGAAG  R: CTCATCAAGACGAGCGAAGTAA |
| Atrazine | MOA-associated & cluster representative (C1) | AT2G36620 | F: CGGTGCTACTTTGGAGGTTAT  R: CTTCTTCTCGTCCTTGGTCTTC |
|  | MOA-associated | AT5G53970 | F: GTTCGCTACGTCGATCTTCTTC  R: CGCAAGGATTACCAGGGTTTAT |
|  | shared function theme | AT1G66540 | F: CCTTGTTAGTCCCACACATCTC  R: GCCCAGACGTTCACCAATAA |
|  | shared function theme | AT3G55710 | F: GAAAGAGCCTGAGGGACTAAAC  R: CTTGTAACTTGCTGCGACAATC |
| Glyphosate | MOA-associated & cluster representative (C1) | AT3G25100 | F: GGCTGGCTTGTGTTTCTTTG  R: TCCCTGAGCTATTGATGTGTTG |
|  | MOA-associated & cluster representative (C1) | AT4G22860 | F: GCACTCCGAAACTACCAGAAT  R: TATACGCATGAGACCGTGTTG |
|  | stress response/shared function theme | AT3G55970 | F: CCATTACCAGCCTTCGTCTTT  R: GTTCTCGCATAGTTTCACCATTTC |
|  | stress response/shared function theme | AT1G01490 | F: AAGGAGAAGCACCGAAGAAG  R: GCGCTTGTGGTTGATCTTTC |
| Imazethapyr | MOA-associated | AT1G03130 | F: TTGAAACTGGCGAGGAAAGA  R: CCGTTAGGGAACACTCTGTAAA |
|  | MOA-associated | AT1G51400 | F: AGATGAGGAGGGATCTTGTCTT  R: CTGTTCCTCTCTTTGGCTCATC |
|  | cluster representative (C2) | AT2G44860 | F: CGACAGGAACGTTACAGAGAAT  R: GCTTGTTTGGCTTCAACCTATT |
|  | shared function theme | AT5G62480 | F: CTACGGGTGGATCAATGCTATAA  R: CTGTCTGAAAGCCCGAAGAA |
| Mesotrione | cluster representative (C2) | AT5G39610 | F: AAGATTGAGCCTTGGGACTTAC  R: CCTTAAACCGGTCGGGTATTT |
|  | cluster representative (C2) | AT4G02075 | F: CTAACATTAAAGGCCATTGGGATAC  R: ATTGATGACGACGACGAAGAG |
|  | cluster representative (C2) | AT1G68670 | F: CAGCTTGGAGGATCTCATGTT  R: GGCCTTCTTGTGTGAAGTCTAT |
| Paraquat | MOA-associated & cluster representative (C1) | AT1G53540 | F: CAGCGTTCACAAACGCTAAAG  R: CTCCACTTTCACCTCTTCCTTC |
|  | MOA-associated | ATCG00350 | F: TCCATTAGGAACGGCAGATTT  R: GCGAGCTACGAGCAAATAGA |
|  | MOA-associated | ATCG00680 | F: CCGTTTAGAAGGGCAGAATCA  R: CACAGTTGCGGGATCACTATAA |
|  | shared function theme | AT1G07890 | F: GGACGATGCCACAAGGATAG  R: TGTCAGAGACAAGCTGAAGAAG |
| Arabidopsis reference gene *PP2A* |  | AT1G13320 | F: TATCGGATGACGATTCTTCGTGCAG  R: GCTTGGTCGACTATCGGAATGAGAG |
