# Supplementary material for: Dose-response-defined physiological framework enables comparative analysis of transcriptional response dynamics across multiple herbicides in Arabidopsis thaliana
Source: Front Plant Sci. 2026 Sep 23;17:1870671. doi: 10.3389/fpls.2026.1870671 (PMC13642330; doi:10.3389/fpls.2026.1870671)
Supplement: Supplementary file 2 [file DataSheet2.zip › Supplementary Tables/Supplementary Table S13.docx]

**Supplementary Table S13a**: Complete DEGs numbers summarized by herbicide under each treatment condition.

| **Herbicide Name** | **Number of complete DEGs** | | | |
| --- | --- | --- | --- | --- |
|  | **IC_50__4 h** | **IC_50__24 h** | **IC_99__4 h** | **IC_99__24 h** |
| **2,4-D** | 405 | 903 | 1116 | 2316 |
| **Acifluorfen** | 324 | 1747 | 1514 | 5977 |
| **Atrazine** | 868 | 1354 | 2362 | 4407 |
| **Glyphosate** | 0 | 2 | 63 | 2137 |
| **Imazethapyr** | 48 | 4262 | 3018 | 7272 |
| **Mesotrione** | 106 | 74 | 393 | 186 |
| **Paraquat** | 304 | 1024 | 1699 | 3950 |

**Supplementary Table S13b**: Unique DEGs numbers (and percentages in complete DEGs numbers) summarized by herbicide under each treatment condition.

| **Herbicide Name** | **Number of Unique DEGs** | | | |
| --- | --- | --- | --- | --- |
|  | **IC_50__4 h** | **IC_50__24 h** | **IC_99__4 h** | **IC_99__24 h** |
| **2,4-D** | 338 (83%) | 245 (27%) | 395 (35%) | 288 (12%) |
| **Acifluorfen** | 109 (34%) | 547 (31%) | 152 (10%) | 880 (15%) |
| **Atrazine** | 795 (92%) | 570 (42%) | 1353 (57%) | 1201 (27%) |
| **Glyphosate** | 0 | 1 (50%) | 5 (8%) | 39 (2%) |
| **Imazethapyr** | 16 (33%) | 2811 (66%) | 1323 (44%) | 2086 (29%) |
| **Mesotrione** | 22 (21%) | 4 (5%) | 15 (4%) | 7 (4%) |
| **Paraquat** | 84 (28%) | 195 (19%) | 189 (11%) | 137 (3%) |
