# Supplementary material for: Dose-response-defined physiological framework enables comparative analysis of transcriptional response dynamics across multiple herbicides in Arabidopsis thaliana
Source: Front Plant Sci. 2026 Sep 23;17:1870671. doi: 10.3389/fpls.2026.1870671 (PMC13642330; doi:10.3389/fpls.2026.1870671)
Supplement: Supplementary file 2 [file DataSheet2.zip › Supplementary Tables/Supplementary Table S14.docx]

**Supplementary Table S14.** Comparison of DEGs identified between mock-treated plants at 24 h vs. 4 h, and between IC_99_-treated vs. mock-treated plants at 24 h, including the number and percentage of overlapping DEGs for each herbicide.

| **Herbicide** | **Mock_24 h vs 4 h DEGs #** | **IC_99__24 h vs Mock_24 h DEGs #** | **Overlapped DEGs # between column 2&3** | **(overlapped DEGs)% in column 3** |
| --- | --- | --- | --- | --- |
| **2,4-D** | 546 | 2316 | 165 | 7.1% |
| **Acifluorfen** | 593 | 5977 | 349 | 5.8% |
| **Atrazine** | 966 | 4407 | 458 | 10.4% |
| **Glyphosate** | 742 | 2137 | 260 | 12.2% |
| **Imazethapyr** | 894 | 7272 | 509 | 7.0% |
| **Mesotrione** | 851 | 186 | 36 | 19.4% |
| **Paraquat** | 938 | 3950 | 457 | 11.6% |
