# Supplementary material for: Dose-response-defined physiological framework enables comparative analysis of transcriptional response dynamics across multiple herbicides in Arabidopsis thaliana
Source: Front Plant Sci. 2026 Sep 23;17:1870671. doi: 10.3389/fpls.2026.1870671 (PMC13642330; doi:10.3389/fpls.2026.1870671)
Supplement: Supplementary file 2 [file DataSheet2.zip › Supplementary Tables/Supplementary Table S15.docx]

**Supplementary Table S15.** Summary of key characteristics and representative genes of each transcriptional cluster identified across herbicides in this study.

NA: no significant terms.

| **Herbicide** | **MoA** | **Cluster** | **Gene Number** | **Responsive Type** | **Dominant GO Terms** | **Biological Effect** | **Representative Genes** |
| --- | --- | --- | --- | --- | --- | --- | --- |
| 2,4-D | Growth regulators (synthetic auxin) | C1 | 256 | Consistent repression | Wounding response, jasmonic acid stimulus response and glucosinolate biosynthetic process | Stress response |  |
|  |  | C2 | 95 | Consistent induction (strong expression) | Auxin responses and hormone responses | Growth regulation | SAUR-like auxin-responsive protein family (AT1G29490, AT1G75590, AT2G18010, AT2G37030, AT4G12410, AT4G13790); Auxin-responsive GH3 family protein (AT2G14960, AT2G23170, AT4G27260, AT4G37390) |
|  |  | C3 | 483 | Consistent induction (moderate or minimal expression) | Auxin responses and hormone responses | Growth regulation | SAUR-like auxin-responsive protein family(AT1G29420, AT1G29460, AT1G56150, AT2G21220, AT3G03830, AT3G03840, AT3G03850, AT4G34770, AT4G36110, AT4G38850, AT5G18010, AT5G18050,  AT5G18060,  AT5G18080,  AT5G20820); Auxin-responsive GH3 family protein (AT1G28130 and AT5G54510) |
| Acifluorfen | PPO inhibitors | C1 | 185 | Time-related induction (strong induction at 24 h) | Responses to different stimulus, including wounding, fungus and jasmonic acid | Stress response | UGT: AT1G07260,  GST: AT5G44990 |
|  |  | C2 | 208 | Dose- and time-related progressive induction | Different stress responses and regulations, including responses to hypoxia, fungus, biotic stimulus, jasmonic acid and salicylic acid | Stress response and regulations | Transcription factors (AT1G18570,  AT1G25560,  AT1G28360,  AT1G28370, AT3G49530,  AT4G31800);Hormone related genes (AT2G44840, AT3G15210, AT5G47220,  AT5G47230,  AT5G61600); HSP (AT1G09080); CYP450s (AT1G64940, AT1G64950, AT4G31950, AT5G67310); UGT (AT2G30140,  AT2G43820); GST (AT1G17180, AT1G69920,  AT1G78340,  AT2G29480, AT2G47730) |
|  |  | C3 | 410 | Late repression at IC_99__24 h | Plastid organization, protein quality control for misfolded/incomplete synthesized proteins (RNA processing and biosynthetic process) | Disruption of chlorophyll and heme biosynthesis and growth inhibition | Chloroplast organization related genes (AT1G20830, AT5G54180, AT2G45770); translation and RNA processing related genes (AT1G76720, AT1G30590, AT1G18440, AT4G38020,  AT1G11870,  AT2G46780,  AT3G46100) |
|  |  | C4 | 706 | Time-related induction (strong induction at 24 h) | Immune response, regulation of defense response and programmed cell death | Stress response, detoxification | PRA1 family genes (AT5G01640, AT5G05987); Rab GTPase family genes (AT1G43890, AT4G29950, AT5G54780); SEC family genes (AT2G01470, AT4G24920); EXO component genes (AT1G47560, AT3G09520, AT3G14090, AT5G58430, AT5G59730); UGTs (AT1G22380, AT2G31750, AT5G03490, AT5G12890); ABC transporters (AT1G59870, AT4G15233, AT5G09930, AT5G24810) |
| Atrazine | Photosystem II inhibitors | C1 | 1296 | Consistent repression with strongest repression at IC_99_  + 24 h | Ribosome biogenesis, translation, DNA replication and chromosome organization | Inhibition of protein synthesis and suppression of cell proliferation | CYP450s (AT1G16400, AT1G16410, AT4G13770,  AT5G07990, AT5G52320); BAHD acyltransferase family genes (AT1G03495,  AT1G03940, AT3G29590); DNA replication related genes (AT1G26840, AT1G67630,  AT2G01120, AT2G31730, AT3G52170,  AT3G56970,  AT3G56980, AT5G22110,  AT5G41880, AT5G61000); chromosome maintenance family genes (AT1G44900, AT2G20980, AT5G44635,  AT5G46280);  176 ribosomal protein family genes (such as AT2G36620, AT4G25890, AT3G06680) |
|  |  | C2 | 1472 | Consistent induction with strongest induction at IC_99_  + 24 h | *L*- and alpha- amino acids catabolism, Responses to absence of light and other stimulus | Stress adaptation and energy management, stress responses | Tyrosine transaminase family protein (AT5G53970); Aldolase (AT4G26530)  Genes response to absence of light (AT1G09570, AT3G17609, AT1G29420, AT3G16770); genes response to different types of stress (CYP450s: AT1G33720,  AT1G66540,  AT3G03470,  AT3G13730,  AT4G37330,  AT5G25140; UGTs: AT1G07250, AT3G55710; TFs: AT1G35516, AT1G70000, AT1G71030, AT4G09460, AT5G07100) |
| Glyphosate | EPSP synthase inhibitors | C1 | 23 | Late repression at IC_99__24 h | Nuclear division, mitotic cell cycle regulation, organelle and chromosome organization | Cell-cycle arrest and inhibition of growth | Cell division/cycle involved genes (AT3G25100, AT4G22860); structural maintenance of chromosome family (AT3G47460) |
|  |  | C2 | 22 | Late induction at IC_99__24 h | NA |  |  |
| Imazethapyr | ALS inhibitors | C1 | 2119 | Time-related repression (strong repression at 24 h) | Photosynthesis, microtubule-based movement and actin cytoskeleton organization | Reduction of photosynthetic capacity and growth inhibition | PSI and PSII components genes (such as PSI D2: AT1G03130, PSII 5kD protein: AT1G51400, PSII light harvesting complex protein: AT2G40100); microtubule-associated proteins (AT4G26760, AT3G50240); actin-binding FH2 proteins (AT3G07540) |
|  |  | C2 | 2290 | Time-related induction (strong induction at 24 h) | Ribosome biogenesis | Stress adaptation  (balance protein homeostasis) | Ribosome protein (AT2G44860, AT3G28500); rRNA processing protein (AT3G22660) |
|  |  | C3 | 160 | Moderate induction at IC_50__4 h and consistent strong induction in the subsequent three treatment conditions | Mitochondrial protein trafficking and ribosome assembly | Adjustment of organellar function and translational capacity |  |
| Mesotrione | HPPD inhibitors | C1 | 5 | Consistent induction (moderate expression) | photosynthetic energy metabolism, redox homeostasis, environmental acclimation, and protein glycosylation | Stress response |  |
|  |  | C2 | 40 | Early responsive, time-related repression (strong repression at 4 h ) | Response to external stimulus and biological regulation | Stress response | NAC TF (AT3G15500 and AT5G39610), RING/FYVE/PHD zinc finger TF (AT4G02075), MYB-like TF (AT1G68670) |
| Paraquat | Photosystem I electron diverter | C1 | 37 | Time-related induction (strong induction at 24 h) | Response to oxidative stress, response to heat, protein folding and maturation | Stress response/adaption | Heat shock proteins (AT1G53540,  AT1G59860,  AT1G74310,  AT2G20560,  AT3G46230,  AT4G25200,  AT5G12030); detoxification genes (ABC-PDR: AT1G15520, GST: AT1G78340, UGTs: AT2G15490,  AT2G36780, CYP450: AT3G26830); stress inducible protein (AT4G12400) |
|  |  | C2 | 184 | Early responsive | Immune response | Stress response | MYB family (AT1G66230,  AT1G71030); MAPK kinase (AT4G08500); photosystem components proteins (ATCG00330,  ATCG00340,  ATCG00350, ATCG00680,  ATCG00720) |
|  |  | C3 | 64 | Consistent induction (moderate expression) | Response to hypoxia, to heat, and to water deprivation, protein folding and autoubiquitination | Stress response/adaption | Detoxification genes (GST: AT1G02920, ascorbate peroxidase: AT1G07890, CYP450s: AT2G29090, AT4G22710, detoxification protein: AT5G03380); stress responsive genes (HSP: AT1G16030, AT2G32120; defense protein related: AT5G48657; calcium-binding proteins: AT1G21550, AT3G29000; TFs: AT1G62300, AT2G46400, AT4G18170, AT3G23250) |
|  |  | C4 | 268 | Time-related induction (strong induction at 24 h) | Response to different biotic and abiotic stress | Stress response |  |
