# Supplementary material for: Dose-response-defined physiological framework enables comparative analysis of transcriptional response dynamics across multiple herbicides in Arabidopsis thaliana
Source: Front Plant Sci. 2026 Sep 23;17:1870671. doi: 10.3389/fpls.2026.1870671 (PMC13642330; doi:10.3389/fpls.2026.1870671)
Supplement: Supplementary file 2 [file DataSheet2.zip › Supplementary Tables/Supplementary Table S2.docx]

**Supplementary Table S2**. Comparison of IC_50_ and IC_99_ values (original Xlfit analysis) used in the RNA-seq experiment with parameter estimates [IC_50_ and IC_99_ values, and 95% Confidence Interval (CI)] obtained by reanalysis of currently available raw dose-response data using the R package *drc* (version 3.0-1).

| **Herbicide Name** | **Xlfit IC_50_** | ***drc* IC_50_ (95% CI)** | **Xlfit IC_99_** | ***drc* IC_99_ (95% CI)** | **Comment** |
| --- | --- | --- | --- | --- | --- |
| **2,4-D** | 50 µM | 66.1 µM (95% CI: 0.5-131.7 µM) | 1500 µM | 6758.1 µM (95% CI: -23297-36813 µM) | upper asymptote not fully captured |
| **Acifluorfen** | 3 µM | 5.0 µM (95% CI: 3.9-6.2 µM) | 114 µM | 62.1 µM (95% CI: -3.2-127.4 µM) |  |
| **Atrazine** | 56 µM | 51.8 µM (95% CI: 38.0-65.5 µM) | 507 µM | 346.1 µM (95% CI: -67.8-759.9 µM) |  |
| **Glyphosate** | 50 µM | 36.0 µM (95% CI: 13.2-58.9 µM) | 1000 µM | 411.2 µM (95% CI: -975.1-1797.6 µM) |  |
| **Imazethapyr** | 2 µM | 1.6 µM (95% CI: 1.2-2.0 µM) | 24 µM | 31.7 µM (95% CI: 0.8-62.7 µM) |  |
| **Mesotrione** | 9 µM | 5.1 µM (95% CI: 0-19.2 µM) | 5000 µM | 7219.6 µM (95% CI: -70717-85156.2 µM) | upper asymptote not fully captured |
| **Paraquat** | 17 µM | 15.5 µM (95% CI: 14.1-17.0 µM) | 45 µM | 49.1 µM (95% CI: 33.7-64.4 µM) |  |
