# Supplementary material for: Dose-response-defined physiological framework enables comparative analysis of transcriptional response dynamics across multiple herbicides in Arabidopsis thaliana
Source: Front Plant Sci. 2026 Sep 23;17:1870671. doi: 10.3389/fpls.2026.1870671 (PMC13642330; doi:10.3389/fpls.2026.1870671)
Supplement: Supplementary file 2 [file DataSheet2.zip › Supplementary Tables/Supplementary Table S3.docx]

**Supplementary Table S3**. Preparation of herbicide treatment solutions used for dose-response and RNA-seq experiments.

| **Herbicide** | **Stock Solution** | **Control Solution** | **IC_99_ Solution** | **IC_50_ Solution** |
| --- | --- | --- | --- | --- |
| **2,4-D** | 1.5 M in 100% ethanol | 1 mL 50% Tween-20 + 0.5 mL 100% ethanol + 498.5 mL water | 100 µL 1.5 M 2,4-D stock + 200 µL 50% Tween-20 + 99.7 mL water, final concentration = 1.5 mM | 3.33 mL IC_99_ solution + 96.67 mL control solution; final concentration ≈ 50 µM |
| **Acifluorfen** | 0.114 M in 100% ethanol | 1 mL 50% Tween-20 + 0.5 mL 100% ethanol + 498.5 mL water | 100 µL 0.114 M Acifluorfen stock + 200 µL 50% Tween-20 + 99.7 mL water; final concentration = 114 µM | 2.63 mL IC_99_ solution + 97.37 mL control solution; final concentration ≈ 3 µM |
| **Atrazine** | 0.5 M in DMSO | 1 mL 50% Tween-20 + 0.5 mL DMSO + 498.5 mL water | 101.4 µL 0.5 M Atrazine stock + 200 µL 50% Tween-20 + 99.7 mL water; final concentration = 507 µM | 11.05 mL IC_99_ solution + 88.95 mL control solution; final concentration ≈ 56 µM |
| **Glyphosate** | 1 M stock solution | 1 mL 50% Tween-20 + 499 mL water | 200 µL 1 M Glyphosate stock + 200 µL 50% Tween-20 + 199.6 mL water; final concentration = 1 mM | 10 mL IC_99_ solution + 190 mL control solution; final concentration = 50 µM |
| **Imazethapyr** | 24 mM in DMSO | 0.4 mL 50% Tween-20 + 0.2 mL DMSO + 199.4 mL water | 100 µL 24 mM Imazethapyr stock + 200 µL 50% Tween-20 + 99.7 mL water; final concentration = 24 µM | 8.33 mL IC_99_ solution + 91.67 mL control solution; final concentration ≈ 2 µM |
| **Mesotrione** |  | 1 mL 50% Tween-20 + 250 mg MES + water to a final volume of 500 mL | 169.66 mg Mesotrione dissolved in MES buffer (prepared as control solution) to prepare 100 mL of a 5 mM solution | 0.18 mL IC_99_ solution + 99.82 mL control solution; final concentration = 9 µM |
| **Paraquat** | 0.18 M in water | 1 mL 50% Tween-20 + 499 mL water | 25 µL 0.18 M Paraquat stock + 200 µL 50% Tween-20 + 99.78 mL water; final concentration = 45 µM | 37.78 mL IC_99_ solution + 62.22 mL control solution; final concentration = 17 µM |
